# Reprogramming systemic and local immune function to empower immunotherapy against glioblastoma

**DOI:** 10.1038/s41467-023-35957-8

**Published:** 2023-01-26

**Authors:** Songlei Zhou, Yukun Huang, Yu Chen, Yipu Liu, Laozhi Xie, Yang You, Shiqiang Tong, Jianpei Xu, Gan Jiang, Qingxiang Song, Ni Mei, Fenfen Ma, Xiaoling Gao, Hongzhuan Chen, Jun Chen

**Affiliations:** 1 Department of Pharmaceutics, School of Pharmacy & Shanghai Pudong Hospital, Fudan University, 201203 Shanghai, China; 2 Key Laboratory of Smart Drug Delivery, Ministry of Education, School of Pharmacy, Fudan University, 201203 Shanghai, China; 3 Department of Pharmacology and Chemical Biology, State Key Laboratory of Oncogenes and Related Genes, Shanghai Universities Collaborative Innovation Center for Translational Medicine, Shanghai Jiao Tong University School of Medicine, 200025 Shanghai, China; 4 Shanghai Center for Drug Evaluation and Inspection, 201210 Shanghai, China; 5 Department of Pharmacy, Shanghai Pudong Hospital, Fudan University, 201399 Shanghai, China; 6 Institute of Interdisciplinary Integrative Medicine Research, Shuguang Hospital, Shanghai University of Traditional Chinese Medicine, 201203 Shanghai, China

**Keywords:** CNS cancer, Immunosuppression, Drug delivery

## Abstract

The limited benefits of immunotherapy against glioblastoma (GBM) is closely related to the paucity of T cells in brain tumor bed. Both systemic and local immunosuppression contribute to the deficiency of tumor-infiltrating T cells. However, the current studies focus heavily on the local immunosuppressive tumor microenvironment but not on the co-existence of systemic immunosuppression. Here, we develop a nanostructure named Nano-reshaper to co-encapsulate lymphopenia alleviating agent cannabidiol and lymphocyte recruiting cytokine LIGHT. The results show that Nano-reshaper increases the number of systemic T cells and improves local T-cell recruitment condition, thus greatly increasing T-cell infiltration. When combined with immune checkpoint inhibitor, this therapeutic modality achieves 83.3% long-term survivors without recurrence in GBM models in male mice. Collectively, this work unveils that simultaneous reprogramming of systemic and local immune function is critical for T-cell based immunotherapy and provides a clinically translatable option for combating brain tumors.

## Abstract (web summary)

Glioblastoma (GBM) is characterized by local and systemic immunosuppression, showing limited responses to immunotherapies. Here the authors describe the design of a nanoplatform composed of the lymphopenia alleviating agent cannabidiol and the lymphocyte recruiting cytokine LIGHT, promoting anti-tumor immune responses in GBM preclinical models.

## Introduction

Glioblastoma (GBM) is the most common and deadliest primary brain malignancy in adults^1,2^. The current standard-of-care for GBM consists of maximum safe surgical resection followed by radiotherapy and temozolomide (TMZ) chemotherapy^2^. Unfortunately, due to the high invasive and heterogeneous nature of GBM, the disease inevitably elapses, and none of the available treatments can effectively prolong survival at recurrence^3–5^. Over the last decades, immunotherapy has revolutionized the treatment of a variety of difficult-to-treat cancers and ushered in a new era. With the ability to eliminate cancer cells and induce immune memory to prevent recurrence, immunotherapy has also garnered considerable interests in the field of GBM therapy^6^. The bulk of current immunotherapies induce or activate effector T cells to exert antitumor activity, whereas GBM is sparsely infiltrated with effector T cells, which largely contributed to the decreased susceptibility to immunotherapy in clinical settings^7–9^. Thus, the development of effective strategies to increase the infiltration of effector T cells is critical for a successful immunotherapy against GBM.

GBM is generally regarded as an immunologically-cold tumor in which the local immunosuppressive tumor microenvironment (TME) is believed to exert resistance to T-cell infiltration^10,11^. The “cold” immune milieu is featured with abundant immunosuppressive cells and factors, including tumor-associated macrophages (TAMs), myeloid-derived suppressor cells (MDSCs), regulatory T cells (Tregs), interleukin-10 (IL-10), and transforming growth factor-β (TGF-β) et al., which are unfavorable for the recruitment and infiltration of effector T cells^6,7,10,12^. In addition, effective antitumor immune response also necessitates a modulated microenvironment^13,14^. Based on these considerations, numerous treatment modalities have been developed to modulate TME to overcome local immune dysfunction and recruit blood-borne T cells. Specially, strategies such as reducing MDSCs^15^, repolarization of M2 phenotype TAMs^16^, delivery of T-cell chemokines^17^, and normalization of tumor vasculature^18^ have been widely applied to relieve immunosuppressive TME, hence improving recruitment condition for effector T cells. Though these techniques reverse local immunosuppression and achieve some success in preclinical studies, the progress made in clinical trials still turns out to be disappointing^6,7^, suggesting regulating local immunosuppression alone is insufficient to strengthen immunotherapy against GBM. The failure of these studies motivates researchers to further explore the probable essential elements impacting immunotherapy in conjunction with clinical pathological characteristics.

Latterly analysis of clinical samples revealed that in addition to local immunosuppression, GBM patients exhibit profound systemic immunosuppression and lymphopenia^19^. In fact, CD4 T cell counts in GBM patients were even close to the lowest levels seen in acquired immune deficiency syndrome individuals^20,21^. Few studies have been conducted on the topic of systemic immunosuppression, and the immunosuppression network involved is still a mystery, with only a handful of studies investigating its underlying mechanisms. Researchers found GBM induced naïve T cells sequestration within bone marrow accompanied by loss of sphingosine-1-phosphate receptor 1, which might be an important factor leading to the reduction of systemic T cells^20^. Systemic immunosuppression was also found to be ascribed to a potent non-steroid factor in serum that inhibited T-cell proliferation^22^, indicating multiple factors were involved in the process. Additionally, the epidemiologic characteristics and clinical treatments of GBM exacerbated systemic immunosuppression. First, the median age diagnosis of GBM is 64 years, and patients are mainly composed of elderly patients with age-related immunosuppression, whose immunity are significantly weaker than that of younger patients, and whose bone marrow and thymus produce significant fewer T cells^23–26^; Second, standard-of-care can cause iatrogenic immunosuppression and lead to systemic lymphopenia, termed treatment-related lymphopenia^7,27,28^; Third, dexamethasone, the most often used to alleviate brain edema in GBM patients, also induces systemic depletion of naïve and memory T cells but increases immunosuppressive myeloid cells^29^. The prevailing immune-modulating therapies mainly focus on remodeling TME, aiming to overcome local immunosuppression to promote effector T cells infiltration and reinforce immune response against tumor^30^. However, the local antitumor immune response relies on peripheral immune cells to drive and sustain^31^, which implies that all of these prospective treatments depend on patients’ intact and functional immune system. Unfortunately, the pathological alterations and clinical treatments induce severe long-lasting systemic immunosuppression in GBM patients, resulting in a reduction in the system’s production of effector T cells. Consequently, the development of therapeutic strategies to overcome systemic immunosuppression might be the key step to further increase the infiltration of effector T cells in the tumor bed and improve the efficacy of immunotherapy against GBM.

**Fig. 1:**
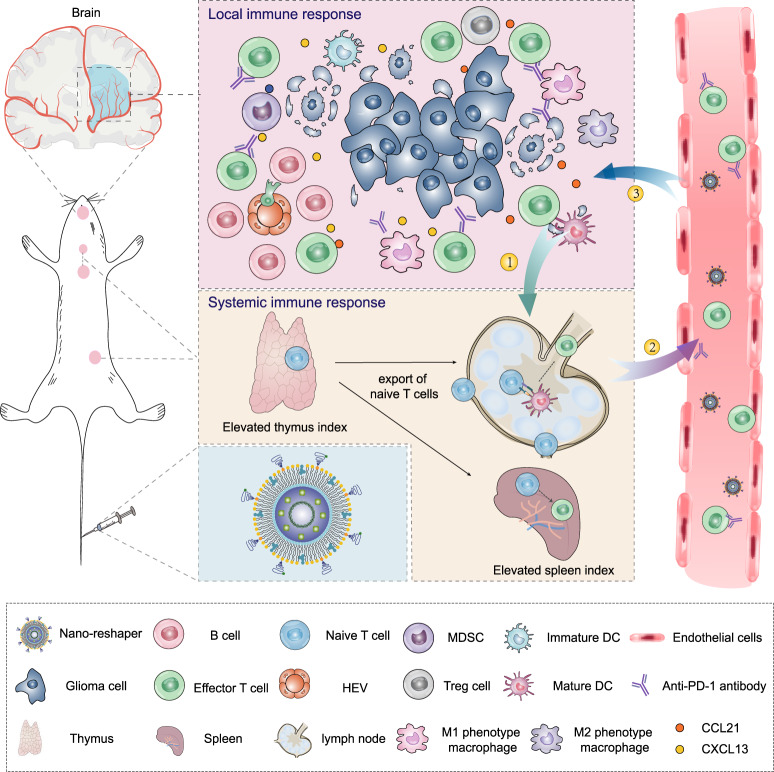
Nano-reshaper reprograms systemic and local immune function to enhance specific antitumor immune response against GBM. Nano-reshaper improves systemic immune function such as enhanced thymus and spleen indexes to provide more systemic effector T cells, and overcomes local immunological dysfunction such as the induction of HEVs and upregulated T-cell chemokines to enable T-cell recruitment and facilitates effector T cells infiltration in tumor site. Finally, the increased effector T cells in GBM enhances the response rate of αPD-1. ①: Tumor antigen uptake and presentation; ②: Effector T cells priming and export; ③: Trafficking and infiltration of effector T cells into tumors.

To empower immunotherapy against GBM, here we propose a nanostructure named Nano-reshaper to simultaneously surmount the systemic and local immunosuppression. Nano-reshaper consists of a small molecule compound cannabidiol (CBD) and a cytokine LIGHT. CBD is a main non-psychoactive cannabinoid component derived from the plant *Cannabis sativa L*.^32^, possessing the biological activities such as anti-oxidant, anti-angiogenic, neuroprotective, and immunomodulatory effects^33,34^. Intriguingly, we find in a preliminary investigation that CBD stimulates the proliferation of T cells in vitro and alleviates systemic lymphopenia in vivo, suggesting that CBD has a systemic immune-enhancing effect. Thus, we intend to utilize CBD to overcome systemic immunosuppression caused by GBM. Tumor necrosis factor superfamily member LIGHT (also named as TNF superfamily member 14, TNFSF14) is an inflammatory cytokine, which binds to lymphotoxin β receptor and herpes virus entry mediator to exert biological activity^35^. LIGHT has been shown to normalize intra-tumoral vasculature and induce high endothelial venules (HEVs) to enable lymphocytes recruitment^36^. However, cytokine-based therapy is restricted by their short half-life, substantial side effects and poor blood–brain barrier (BBB) penetration ability after systemic administration^37^. Nanoparticles possess the advantage of improving aqueous dissolution and bioavailability, mitigating toxicity and enhancing therapeutic efficacy of the encapsulated agent^30,38,39^. Meanwhile, equipped nanoparticles can achieve combinational delivery of therapeutic agents with satisfactory results^40^. To improve the delivery of CBD and LIGHT in vivo, we develop a CBD prodrug (CP) and choose a plasmid encoding LIGHT (pLIGHT), and utilize lipid calcium phosphate (CaP) technology, widely used for efficient delivery of gene and small molecules with phosphate groups while maintaining high safety^41–43^, to encapsulate CP and pLIGHT to obtain pLIGHT@CaCP. This design takes the following factors into consideration: CBD enhances the phagocytic activity of APCs, and the co-delivery can increase the transfection efficiency of pLIGHT in orthotopic GBM; LIGHT can promote APCs maturation and T cells activation^44,45^, which can synergize with CBD to overcome systemic immunosuppression. Apolipoprotein E (ApoE) peptide is modified on pLIGHT@CaCP to obtain ApoE-pLIGHT@CaCP to enhance its BBB crossing ability. Figure 1 depicts the potential therapeutic efficacy of ApoE-pLIGHT@CaCP. Considering the reshaped systemic and local immune function by ApoE-pLIGHT@CaCP, we name it as “Nano-reshaper”. Nano-reshaper is expected to overcome immunosuppression both systemically and locally, and it could be employed as a therapeutic platform for GBM, which indicates a promising way for T-cell based immunotherapy in clinical translation.

## Results

### The preparation and characterization of Nano-reshaper

**Fig. 2:**
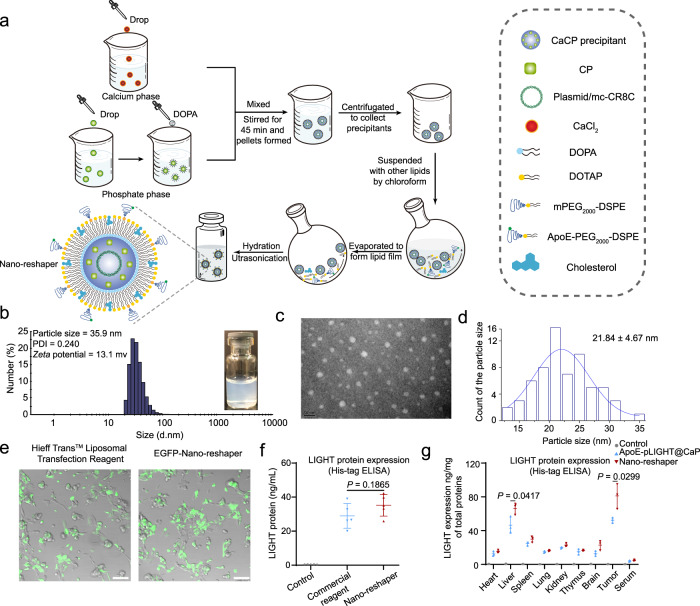
Preparation and characterization of Nano-reshaper. **a** Scheme for the preparation process of Nano-reshaper. **b** Size distribution of Nano-reshaper detected by DLS and visual appearance. **c**, **d** TEM image and particle size distribution. The experiments were repeated three times independently. **e** Representative images of GL261 cells transfected by EGFP coding Nano-reshaper in GL261 cells. Scale bar, 100 μm. The experiments were repeated three times independently. **f** His (6×)-tag ELISA was conducted to evaluate the expression of LIGHT in GL261 cells after incubation with Hieff Trans^TM^ Liposomal Transfection Regent or Nano-reshaper (*n* = 5 samples per group). Ns, not significant. **g** Expression of LIGHT in various organs and serum after two injections of ApoE-pLIGHT@CaP and Nano-reshaper were determined by His (6×)-tag ELISA (*n* = 3 mice). Data were shown as mean ± SD. Error bars represent SD. Significant difference was evaluated in **f** and **g** using two-tailed unpaired Student’s *t* test. Source data are provided as a Source Data file.

The preparation process of Nano-reshaper was shown in Fig. 2a. In brief, a drug-loaded calcium phosphate core was synthesized using the reversed-phase microemulsion method, and the resultant precipitate was then combined with additional lipids in chloroform to form a thin film under high vacuum. After that, 5% glucose (Glu) solution was added to hydrate lipid film to obtain Nano-reshaper. ApoE-CaCP (without pLIGHT) and ApoE-pLIGHT@CaP (without CP) were synthesized using the identical procedure, with the exception of the different components in cores. Since the inner calcium phosphate cores were precipitated via the phosphate group’s interaction with calcium ions^46^, it was challenging to encapsulate and formulate lipophilic CBD for systemic delivery. Thus, we phosphorylated CBD via the hydroxyl groups to maximize loading efficiency (LE) by precipitating with calcium to form an amorphous core (Supplementary Fig. 1a). The obtained CP was confirmed by mass spectrum, ^1^H-NMR and ^13^C-NMR (Supplementary Figs. 1b, c and 2–4). In addition, we found that CP showed reduced cytotoxic effect on GL261 cells compared to CBD (Supplementary Fig. 5), which might be that CP needed to be converted to active compound CBD within the cells.

To enhance GBM targeting ability of Nano-reshaper, we synthetized ApoE-conjugated polyethylene glycol_2000_-1,2-distearoyl-sn-glycero-3-phosphoethanolamine (ApoE-PEG_2000_-DSPE) by utilizing Michael addition reaction (Supplementary Fig. 6a). The maleimide peak (6.99 ppm) of maleimide group in *N*-(maleimide polyethylene glycol_2000_)-1,2-distearoyl-sn-glycero-3-phosphoethanolamine (Mal-PEG_2000_-DSPE) (Supplementary Figs. 6c and 8) was found completely disappeared in ^1^H-NMR spectra of ApoE-PEG_2000_-DSPE (Supplementary Figs. 6d and 9), along with the emergence of characteristic peaks of Mal-PEG_2000_-DSPE and ApoE (Supplementary Figs. 6b–d, 7, and 8), indicating the successful conjugation of ApoE to Mal-PEG_2000_-DSPE. ApoE-PEG_2000_-DSPE was purified to >95% as assessed by the fluorescence intensity of tryptophan on ApoE peptide. Given that ApoE density may affect its capacity to target GBM, we examined the cellular absorption of ApoE-conjugated CaP nanoparticles on GL261 and bEnd.3 cells. The data revealed that no further increase in cellular absorption occurred beyond a 5 mol% ApoE alteration (Supplementary Fig. 10). Thus, in the next studies, a density of 5 mol% was used.

The particle size of the produced Nano-reshaper was about 35 nm or 20 nm as determined by dynamic light scattering detector (DLS) or transmission electron microscope (TEM) and the surface charge was about 13 mv (Fig. 2b–d and Supplementary Table 1), and Nano-reshaper exhibited good stability (Supplementary Fig. 11). The encapsulation efficiency (EE) of plasmid and CP were 51.7 ± 4.8% and 53.4 ± 5.5%, respectively (Supplementary Table 1). The LE of plasmid and CP were 0.21 ± 0.02% and 1.45 ± 0.18%, respectively (Supplementary Table 1). The two drugs released slowly from Nano-reshaper at pH 7.4 (mimicking physiological circumstances) and pH 6.5 (mimicking TME), but the release rate rose dramatically at pH 5.0 (mimicking lysosomes; Supplementary Fig. 12), indicating that the release of the encapsulated drugs was pH-dependent. To further validate the delivery of the two payloads to cells in TME, we labeled the plasmids encapsulated inside Nano-reshaper with YOYO-1 and the carrier with DiD. The colocalization of DiD and YOYO-1 signals (88.6 ± 3.3%) in cells by confocal microscope revealed that Nano-reshaper did not release most of its contents in TME prior to cellular absorption by cells in tumor site (Supplementary Fig. 13). To investigate the intracellular conversion of CP to CBD, we incubated RAW264.7, BV2, DC2.4, and GL261 cells with Nano-reshaper, and successfully detected CBD in these cells as demonstrated by the CBD peak, which suggested that CP can be converted to the active drug CBD in cells (Supplementary Fig. 14). To determine the transfection efficiency of Nano-reshaper, we used plasmid coding LIGHT sequence to construct Nano-reshaper (Supplementary Fig. 15a), and utilized another plasmid which can simultaneously encoding enhanced green fluorescent protein (EGFP) and LIGHT sequence to construct EGFP-Nano-reshaper (Supplementary Fig. 15b). EGFP-Nano-reshaper exhibited comparable transfection efficiency to Hieff Trans^TM^ Liposomal Transfection Reagent in GL261 cells (Fig. 2e and Supplementary Fig. 15c, d). And Nano-reshaper was able to transfect and secrete LIGHT in GL261 cells (Fig. 2f). Immunohistochemistry and enzyme-linked immunosorbent assay (ELISA) were further used to explore the transfecting ability of Nano-reshaper in mice bearing intracranial GL261 GBM. After two injections, higher EGFP signals were observed in EGFP-Nano-reshaper group compared to EGFP-ApoE-pLIGHT@CaP group (Supplementary Fig. 16a, b). Specially, EGFP was expressed by tumor cells, endothelial cells, macrophages and dendritic cells (DCs) (Supplementary Fig. 16c–f). ELISA confirmed that LIGHT was successfully secreted in brain tumors (Fig. 2g), and the expression level in Nano-reshaper group was also higher than in ApoE-pLIGHT@CaP group, suggesting encapsulated CP improved the transfection efficiency. Collectively, these results demonstrated that Nano-reshaper was successfully constructed and was able to achieve in situ expression in GBM.

### ApoE peptide enabled the GBM and peripheral immune cells targeting delivery of Nano-reshaper

**Fig. 3:**
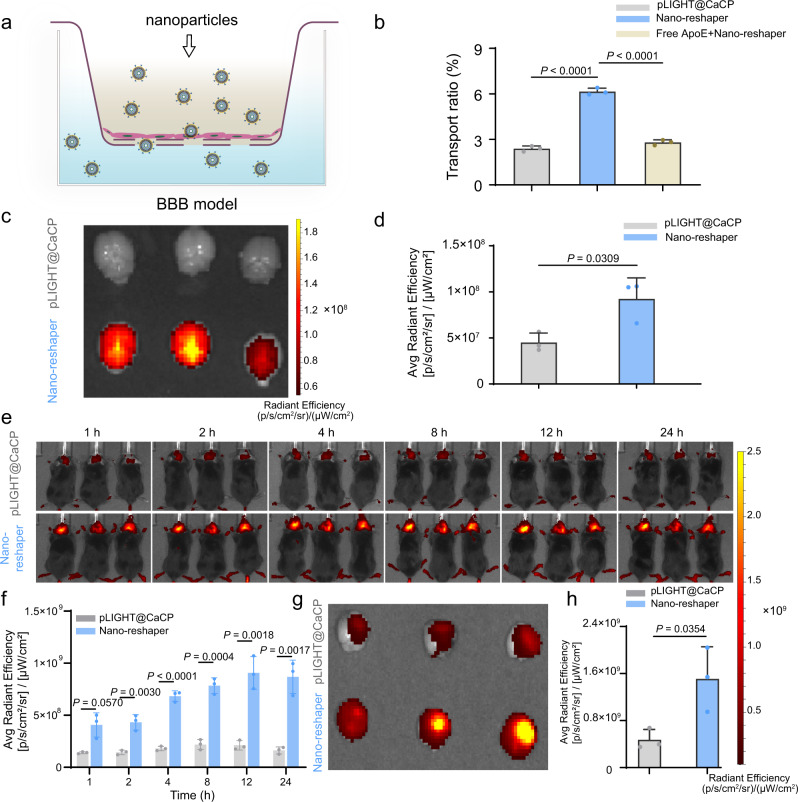
ApoE peptide enabled the brain targeting delivery of Nano-reshaper. **a** Scheme to illustrate DiI-labeled CaP nanoparticles crossing BBB model constructed by bEnd.3 cells. **b** The transport ratio (%) of DiI-labeled pLIGHT@CaCP and Nano-reshaper following 4 h incubation in vitro model. Blockade experiment was performed by pretreating bEnd.3 cells with free ApoE peptide (100 μg/mL) for 1 h before Nano-reshaper incubation (*n* = 3 samples per group). **c** Ex vivo DiR fluorescence imaging of excised brains obtained from healthy mice killed at 24 h post-injection. **d** Semi-quantitative results of the ex vivo brain radiant efficiency shown in **c** (*n* = 3 mice). **e** Real-time whole-body DiR fluorescence imaging of mice bearing intracranial GL261 GBM after *i.v*. injection of DiR-labeled pLIGHT@CaCP and Nano-reshaper. **f** Semi-quantitative results of the in vivo brain radiant efficiency (*n* = 3 mice). **g** Ex vivo DiR fluorescence imaging of excised brains obtained from mice bearing intracranial GL261 GBM killed at 24 h post-injection. **h** Semi-quantitative results of the ex vivo brain radiant efficiency shown in **g** (*n* = 3 mice). Data were shown as mean ± SD. Error bars represent SD. Significant difference was evaluated in **b**, **d**, **f**, and **h** using two-tailed unpaired Student’s *t* test. Source data are provided as a Source Data file.

Although BBB is thought to be compromised in brain tumors, it remains intact in the majority of tumor sites, posing a considerable barrier for therapeutic medication delivery into GBM^47^. Thus, we next investigated the GBM targeting ability of Nano-reshaper. First, the BBB-crossing capacity of Nano-reshaper was assessed by an in vitro BBB model (Fig. 3a). Nano-reshaper demonstrated 1.6-fold more penetration than pLIGHT@CaCP (Fig. 3b). Additionally, pre-treatment with free ApoE peptide greatly decreased the transport ratio of Nano-reshaper (Fig. 3b), suggesting that Nano-reshaper may traverse the BBB via ApoE receptors.

Following that, we examined the brain distribution of Nano-reshaper in tumor-free mice to determine its ability to penetrate the BBB in vivo. CaP nanoparticles were labeled using the near infrared fluorescent probe DiR. In comparison to pLIGHT@CaCP, Nano-reshaper achieved a 1.0-fold increase in DiR signals in the brain (Fig. 3c, d). To further assess their potential to target GBM, DiR-labeled Nano-reshaper and pLIGHT@CaCP were administered to C57BL/6 mice bearing intracranial GL261 GBM, and Nano-reshaper accumulated more in brain tumors than pLIGHT@CaCP from 1 to 24 h after injection (Fig. 3e, f). This finding was confirmed by ex vivo imaging at 24 h post-injection (Fig. 3g, h and Supplementary Fig. 17a, b).

In addition, we analyzed the distribution of Nano-reshaper in specific cells in brain tumors and key immune organs including spleens, lymph nodes and thymuses. The mean fluorescence intensity (MFI) of DiR was higher in Nano-reshaper group than that of pLIGHT@CaCP group in T cells, macrophages, DCs and tumor cells (Supplementary Fig. 17c–f), indicating that the decoration of ApoE peptide enhanced the targeting ability of nanoparticles to these cells. Altogether, these results revealed that ApoE peptide enabled the GBM targeting and increased the accumulation of Nano-reshaper in antigen-presenting cells (APCs) and T cells both in the orthotopic GBM and peripheral immune organs.

### Nano-reshaper improved systemic immune response in GBM-bearing mice

To promote T-cell proliferation and alleviate lymphopenia to strengthen systemic immune response against GBM, we originally explored a series of potential small compounds before identifying CBD. To reveal the effect of CBD on T-cell proliferation, we cultured carboxyfluorescein succinimidyl ester (CFSE) labeled T cells obtained from naïve mice in the presence of anti-CD3/CD28 antibody (αCD3/CD28) and serum isolated from experiment mice, along with different concentration of CBD. CFSE signals demonstrated that αCD3/CD28 stimulation promoted the activation and proliferation of both CD8^+^ and CD4^+^ T cells (Supplementary Fig. 18). In accordance with the previous report^22^, serum of GL261-bearing mice suppressed T-cell proliferation compared to serum obtained from tumor-free mice (Supplementary Fig. 18). Notably, T-cell proliferation was increased dramatically with the elevating CBD concentration. These data indicated that CBD promoted T-cell proliferation under the systemic immunosuppressive factors of GBM-bearing mice.

The effect of CBD on T-cell proliferation drew our attention for its value in alleviating lymphopenia. To investigate the immunoregulatory effect of CBD on GBM in vivo, we synthesized CP and constructed ApoE-CaCP. The in vivo experiment showed that no significant difference of tumor growth was observed among various dose of ApoE-CaCP treated mice (Supplementary Fig. 19a–c). As previously reported, spleen and thymus involution was observed in patients with GBM^20^, as evidenced by the reductions of both organ weight and total cell counts after the implantation of GL261-luc cells in brain (Supplementary Fig. 19d–i), as well as decreases in the frequency and total counts of CD8^+^ and CD4^+^ T cells in blood in 5% Glu group compared to sham group (Supplementary Figs. 19m and 20a, b). Notably, the spleen and thymus indexes were elevated in ApoE-CaCP-treated mice (Supplementary Fig. 19e, h), particularly in the 10 mg/kg group, indicating that immune function was strengthened following ApoE-CaCP therapy. Thus, we analyzed the cell counts of thymuses and spleens and discovered that T-cell counts in thymuses were increased up to 2.0-fold, 6.6-fold, and 5.7-fold in 5 mg/kg, 10 mg/kg, and 20 mg/kg groups, respectively, when compared to 5% Glu group (Supplementary Fig. 19j); total cell counts, CD8^+^ and CD4^+^ T-cell counts in spleens in 10 mg/kg group were even close to those in sham group (Supplementary Fig. 19f, k). In addition, the frequency and total counts of CD8^+^ and CD4^+^ T cells in blood were both upregulated in mice receiving ApoE-CaCP, especially in the 10 mg/kg group (Supplementary Figs. 19m and 20a, b). Reductions of naïve CD8^+^ and CD4^+^ T-cell counts in spleens and blood were also observed in mice bearing orthotopic GBM (Supplementary Fig. 19l, n), which might be related to the thymus involution (Supplementary Fig. 19g). And we found that naïve CD8^+^ and CD4^+^ T-cell counts were significantly increased in spleens and blood of mice receiving 10 mg/kg ApoE-CaCP treatment along with the increased thymus index (Supplementary Fig. 19l, n). Notably, the T-cell counts in peripheral blood in 10 mg/kg group were comparable to those in sham group, suggesting that lymphopenia was remarkably alleviated after the treatment. To exclude the contribution of carrier particle on the immune activation, we also investigated the effect of ApoE-CaP on antitumor effect and immune cells. However, no significant difference was observed in antitumor activity, immune organ indexes and T cells in peripheral blood and brain tumors (Supplementary Fig. 21). As ApoE-CaCP efficiently increased CD8^+^ and CD4^+^ T cells in blood circulation (Supplementary Fig. 20a, b), we continued to analyze T-cell infiltration in brain tumors. However, no significant difference of CD8^+^ and CD4^+^ T cells were observed in brain tumors between various dose of ApoE-CaCP group compared with those in 5% Glu group (Supplementary Fig. 20c, d). Collectively, these results suggested ApoE-CaCP alleviated lymphopenia and the optimum dose of CP in vivo application was 10 mg/kg, but ApoE-CaCP still failed to increase T-cell infiltration in brain tumors.

**Fig. 4:**
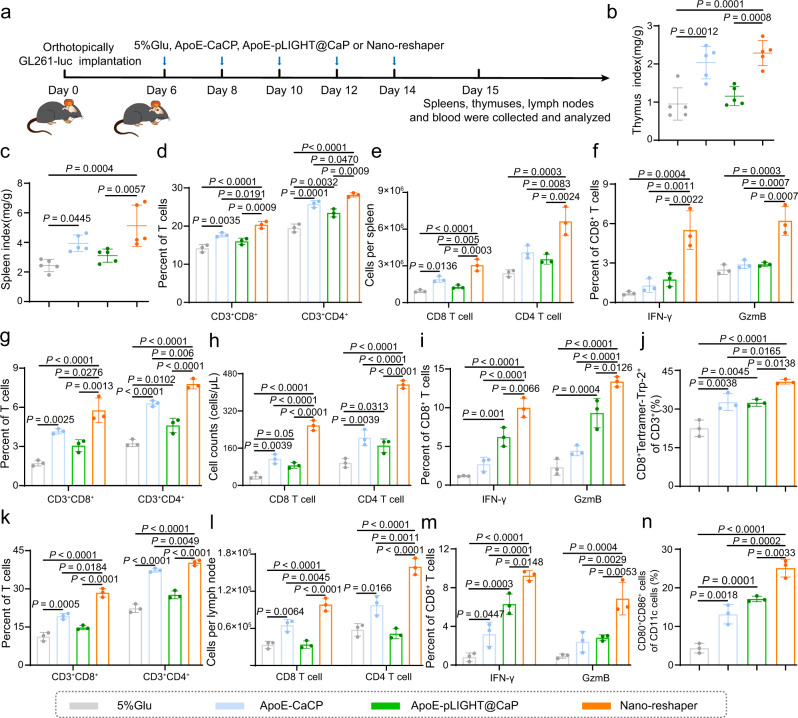
Nano-reshaper improved systemic immune response in GBM-bearing mice. **a** Orthotopic tumor implantation and treatment scheme for 5% Glu, ApoE-CaCP, ApoE-pLIGHT@CaP and Nano-reshaper therapy. Thymus index (**b**) and spleen index (**c**) of intracranial GL261 GBM-bearing mice receiving various formulations on day 15 (*n* = 5 mice). CD8^+^ and CD4^+^ T cells in spleens (**d**), peripheral blood (**g**), and DLNs (**k**) collected from GL261-bearing mice receiving various formulations on day 15 (*n* = 3 mice). CD8 and CD4 T cell counts in spleens (**e**), peripheral blood (**h**), and DLNs (**l**) obtained from intracranial GL261 GBM-bearing mice receiving various formulations on day 15 (*n* = 3 mice). Activation status of CD8^+^ T cells in spleens (**f**), peripheral blood (**i**), and DLNs (**m**) was analyzed by staining for IFN-γ and GzmB after receiving various formulations on day 15 (*n* = 3 mice). Trp-2 specific CD8^+^ T cells in blood (**j**) and CD80^+^CD86^+^ DCs in DLNs (**n**) obtained from intracranial GL261 GBM-bearing mice receiving various formulations on day 15 (*n* = 3 mice). Data were shown as mean ± SD. Error bars represent SD. Significant differences were evaluated in **b**–**n** using one-way ANOVA with Tukey multiple comparisons post-test. Source data are provided as a Source Data file.

Then we further determined the effect of Nano-reshaper on systemic immune response based on the optimum dose of CP (Fig. 4a). The spleen and thymus indexes of GBM-bearing mice treated with ApoE-CaCP or Nano-reshaper were considerably increased after five injections (Fig. 4b, c), indicating the improved systemic immunity. Thus, we examined CD8^+^ and CD4^+^ T cells in the spleens, peripheral blood, and draining lymph nodes (DLNs) of mice treated with Nano-reshaper and discovered that both the percentage and number of CD8^+^ and CD4^+^ T cells, as well as interferon-γ (IFN-γ)^+^ and granzyme B (GzmB)^+^ of CD8^+^ T cells, the activation status of CD8^+^ T cells, were significantly increased (Fig. 4d–m and Supplementary Fig. 22). The upregulated CD80^+^CD86^+^ DCs in DLNs also supported the enhanced systemic immune response (Fig. 4n and Supplementary Fig. 22).

To further demonstrate the improved specific immune response against tumor, we employed H-2K^b^ Trp-2 tetramers antibody to evaluate tumor-antigen specific CD8^+^ T cells, since Trp-2 antigen was a significantly expressed tumor-associated antigen on GL261 cells^48^. As expected, mice treated with Nano-reshaper experienced an increase in tumor-specific CD8^+^ T cells in peripheral blood (Fig. 4j and Supplementary Fig. 22c). Notably, the systemic immune response observed in Nano-reshaper group was greater than that observed in ApoE-CaCP and ApoE-pLIGHT@CaP group (Fig. 4d–n), proving the synergistic effect of CBD and LIGHT. Altogether, these results demonstrated that Nano-reshaper enhanced systemic immune response against tumor in GBM-bearing mice.

### Nano-reshaper promoted the activation of APCs

The phenomenon of an increased tumor-specific immune response prompted us to explore the activation effect of Nano-reshaper on APCs, which play a critical role in the initiation of antitumor T cell immunity. Importantly, we have discovered the effect of CBD on antigen uptake and cross-presentation by APCs. All the investigated concentrations were all below the lethal dose (Supplementary Fig. 23a–d). Coumarin 6 (cou6) labeled methoxy poly (ethylene glycol)_3000_-poly (lactic acid)_34000_ (mPEG-PLA) nanoparticles was used to assess the phagocytosis of APCs. Cou6 fluorescence intensity in RAW264.7, BV2, and DC2.4 cells were much higher in CBD-treated group compared to those in control group, which meant more cou6-labeled mPEG-PLA nanoparticles were engulfed by RAW264.7, BV2, and DC2.4 cells (Supplementary Figs. 23e and 24a–c). This indicated that the phagocytic capacity of these cells was enhanced by CBD. Cellular inhibition studies revealed that amiloride and sodium azide (NaN_3_) + deoxyglucose (DOG) greatly inhibited the enhanced absorption of cou6-labeled mPEG-PLA nanoparticles in RAW264.7, BV2, and DC2.4 cells, suggesting that CBD triggered a macropinocytosis-mediated endocytic process (Supplementary Fig. 25). CBD is a transient receptor potential vanilloid 2 (TRPV2) agonist, and TRPV2 enhances Ca^2+^ penetration into cells and increases particle binding and phagocytosis of macrophages^49,50^. Here, we found intracellular calcium concentration was significantly upregulated in DC2.4 cells after treatment with CBD (Supplementary Fig. 26), suggesting that calcium may be involved in the enhanced macropinocytosis-mediated endocytic process^51^.

Given that DCs are essential for initiating and directing adaptive immune responses and that Ca^2+^ plays a critical role in DC maturation^52^, we studied the effect of CBD on DC maturation and discovered that CBD efficiently elevated CD86 expression and promoted maturity in DC2.4 cells (Supplementary Fig. 24f). To evaluate the effect of CBD on antigen cross-presentation, ovalbumin (OVA) was chosen as the model antigen and incubated with DC2.4 cells after pre-treatment with CBD. Flow cytometry and confocal microscopy analysis found that the cross-presentation ability of DC2.4 cells was improved (Supplementary Fig. 24d, e, g). These results indicated that CBD increased intracellular Ca^2+^ concentration, enhanced macropinocytosis-mediated endocytic process of APCs to engulf exogenous antigen, facilitated DCs maturation and improved antigen cross-presentation ability.

**Fig. 5:**
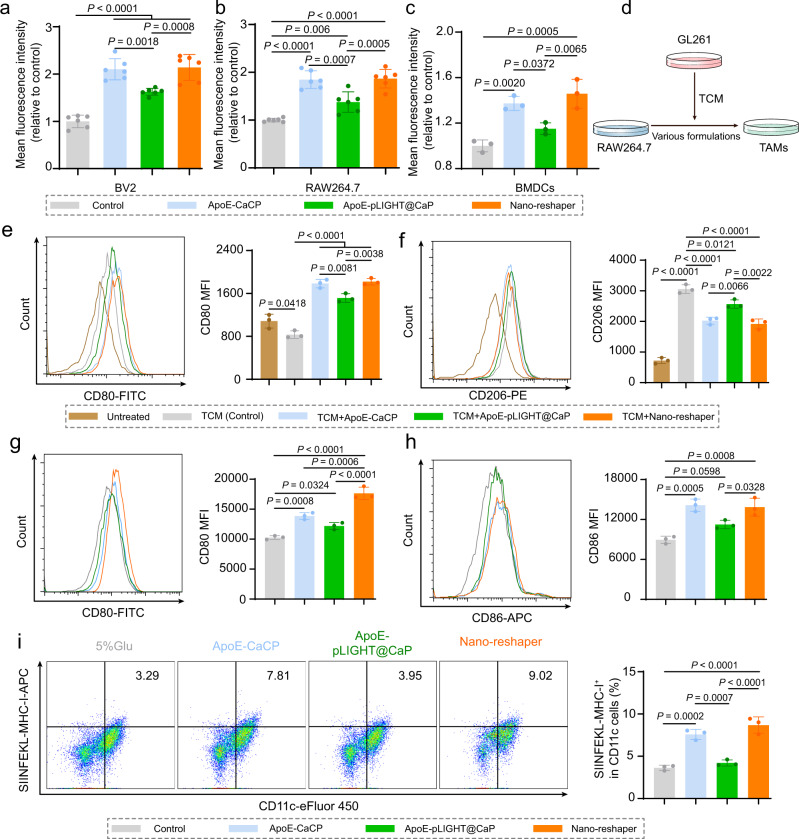
Nano-reshaper promoted the activation of APCs. Quantitative cellular uptake of cou6-labeled mPEG-PLA nanoparticles by **a** BV2 cells (*n* = 6), **b** RAW264.7 cells (*n* = 6 samples per group), and **c** BMDCs (*n* = 3 samples per group). **d** A scheme for illustration of TAMs polarization investigation. MFI of CD80 (**e**) and CD206 (**f**) expression in TAMs by flow cytometry analysis (*n* = 3 samples per group). MFI of CD80 (**g**) and CD86 (**h**) expression in BMDCs by flow cytometry analysis (*n* = 3 samples per group). **i** Flow cytometry analysis of SIINFEKL-MHC-I positive cells in BMDCs (*n* = 3 samples per group). Data were shown as mean ± SD. Error bars represent SD. Significant differences were evaluated in **a**–**c** and **e**–i using one-way ANOVA with Tukey multiple comparisons post-test. Source data are provided as a Source Data file.

Then we explored the effect of ApoE-CaCP and Nano-reshaper on the activation of APCs. ApoE-CaCP and Nano-reshaper groups exhibited considerably higher cou6 fluorescence intensity than control group, indicating that encapsulated CP greatly boosted the phagocytic potential of RAW264.7, BV2, and BMDCs (Fig. 5a–c and Supplementary Fig. 27). TAMs are the dominant immune cells in GBM and tend to serve as M2 phenotype, playing an immunosuppressive role in TME^6^. Thus, we further investigated whether Nano-reshaper could reduce M2 phenotype TAMs. Tumor-conditioned medium (TCM) collected from GL261 cells were used to induce M2 phenotype TAMs (Fig. 5d). Reduced CD206 expression but increased CD80 expression were observed on TAMs following treatment with ApoE-CaCP, ApoE-pLIGHT@CaP, and Nano-reshaper (Fig. 5e, f), indicating that these formulations inhibited the polarization of M2 phenotype and facilitated M1 phenotype. Nano-reshaper also significantly increased the expression of CD80, CD86 and SIINFEKL-MHC-I in BMDCs (Fig. 5g–i), indicating that the degree of maturation and antigen cross-presentation of BMDCs were improved. These findings indicated that Nano-reshaper activated and enhanced the immunological activity of APCs through the effect of CBD, which may be beneficial to initiate the specific antitumor immune response.

### Nano-reshaper reprogrammed TME to strengthen local immune response against GBM

**Fig. 6:**
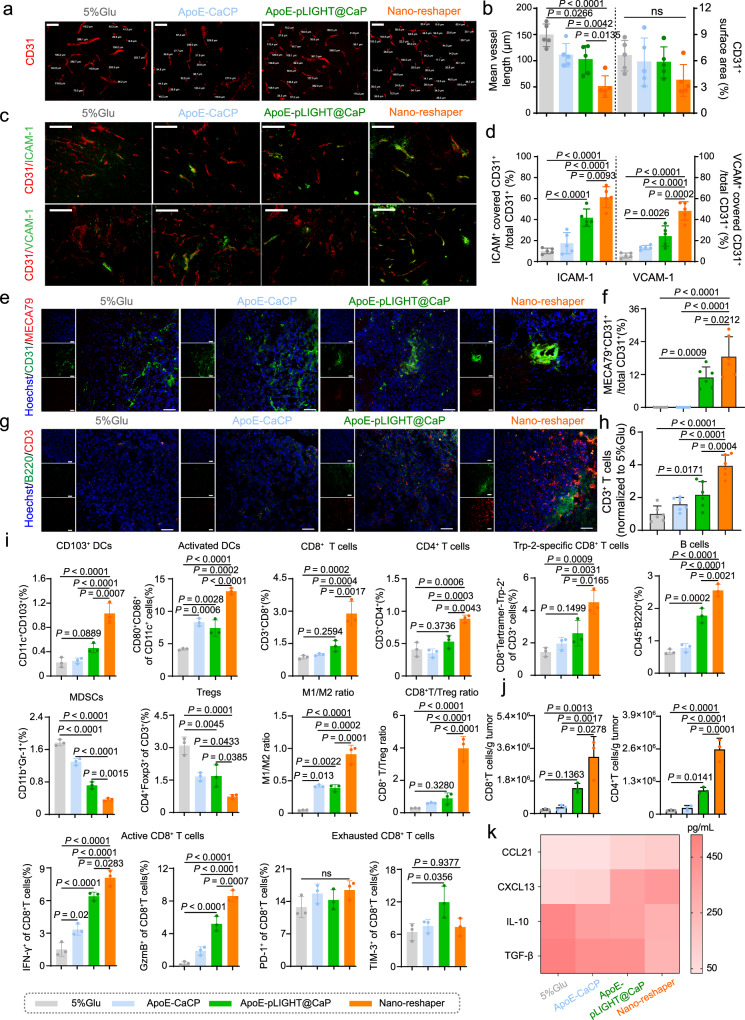
Nano-reshaper reprogrammed TME to strength local immune response again GBM. **a**, **b** Immunohistochemical analysis of tumor vascularity (CD31^+^ staining) in mice treated with various formulations (*n* = 5 samples per group). Scale bar, 100 µm. The experiments were repeated three times independently. **c**, **d** Immunohistochemical analysis of cell adhesion molecules on vessel (CD31, ICAM-1, and VCAM-1 staining) (*n* = 5 samples per group). Scale bar, 100 µm. The experiments were repeated three times independently. Data in **b** and **d** were analyzed by Image J software and shown as mean ± SD. **e**, **f** Tumor sections were stained with anti-CD31 (green) and anti-MECA79 (red) antibody and analyzed by Image J software (*n* = 6 samples per group). Scale bar = 50 μm. The experiments were repeated three times independently. **g**, **h** Tumor sections were stained with anti-B220 (green) and anti-CD3 (red) antibody and analyzed by Image J software (*n* = 6 samples per group). Scale bar = 50 μm. The experiments were repeated three times independently. **i** CD103^+^ DCs, activated DCs, CD8^+^ T cells, CD4^+^ T cells, Trp-2 specific CD8^+^ T cells, B cells, MDSCs, Tregs, M1/M2 ratio, CD8^+^ T/Treg ratio, active and exhausted CD8^+^ T cells in tumors after receiving various formulations on day 15, analyzed by flow cytometry (*n* = 3 mice). **j** Flow cytometric analysis of absolute number of CD8^+^ and CD4^+^ T cells in tumors (*n* = 3 mice). **k** Chemokines related with lymphocyte recruitment and cytokines involved in immunosuppression in brain tumors were regulated in local TME (*n* = 6 samples per group). Data were shown as mean ± SD. Error bars represent SD. Significant differences were evaluated in **b**, **d**, **f**, and **h**–**k** using one-way ANOVA with Tukey multiple comparisons post-test. Source data are provided as a Source Data file.

After determining the impact of Nano-reshaper on systemic immune response against GBM, we continued to examine its impact on the local immune response. Blood vessels are functionally abnormal in GBM, leading to the resistance of T-cell infiltration^18,53^. The effect of Nano-reshaper on the structure of tumor blood vessels was determined using CD31, ICAM-1, and VCAM-1 as the markers. There was no significant change in CD31 positive surface area following treatment with various formulations (Fig. 6a, b), however tumor blood vessels became more homogeneous in structure and decreased length following Nano-reshaper therapy (Fig. 6a, b). Additionally, the expression of ICAM-1 and VCAM-1, two major adhesion factors involved in T cell transendothelial migration from the bloodstream^12^, was considerably elevated in Nano-reshaper group compared to 5% Glu group (Fig. 6c, d). These findings demonstrated that Nano-reshaper aided in the normalization of blood vessels in GBM.

Along with controlling blood vessels, HEVs marked by MECA79 were detected in tumors of mice treated with ApoE-pLIGHT@CaP and Nano-reshaper, but not tumors of mice treated with 5% Glu and ApoE-CaCP (Fig. 6e, f). In general, medically induced HEVs increased immune cells infiltration and anticancer immunity in malignancies^36,54^. ApoE-pLIGHT@CaP and Nano-reshaper were found to boost the expression of critical chemokines, CXCL13 and CCL21, for T and B cells recruitment (Fig. 6k), resulting in increased T and B cells infiltration (Fig. 6g, h). Additionally, flow cytometry study revealed that Nano-reshaper dramatically boosted CD11c^+^CD103^+^ cells, the cross-presenting DCs, in brain tumors (Fig. 6i). Meanwhile, the mature DCs, CD80^+^CD86^+^ cells, were considerably enhanced in tumors following Nano-reshaper therapy (Fig. 6i and Supplementary Figs. 28 and 29), enabling the development of a specific antitumor immune response. This was in accordance with the increased percentage and number of CD8^+^ and CD4^+^ T cells in tumors (Fig. 6i, j and Supplementary Figs. 28–30). Nano-reshaper also increased activation status of CD8^+^ T cells as measured by the expression of IFN-γ^+^ and GzmB^+^, but with no significant impact on immune checkpoints PD-1 and TIM-3 (Fig. 6i). Specifically, Nano-reshaper induced more Trp-2-specific CD8^+^ T cells in tumors than 5% Glu, ApoE-CaCP and ApoE-pLIGHT@CaP (Fig. 6i and Supplementary Fig. 28), demonstrating the enhanced anti-GBM specific immune response.

The effect of Nano-reshaper on the immunosuppressive factors in TME was investigated, and Nano-reshaper drastically decreased Treg, M2 phenotype TAMs, and MDSCs, the primary immunosuppressive cells in TME, while increasing M1 phenotype TAMs (Fig. 6i). TGF-β and IL-10, two main immunosuppressive cytokines in GBM^7^, were dramatically reduced following Nano-reshaper therapy (Fig. 6k). In addition, the increased CD8^+^/Treg index (Fig. 6i), positive with prolonged survival^55^, were also observed in Nano-reshaper group. Taken together, our findings indicated that Nano-reshaper remodeled immunosuppressive TME and strengthened the local immune response against GBM.

### Nano-reshaper inhibited GBM growth and improved animal survival

**Fig. 7:**
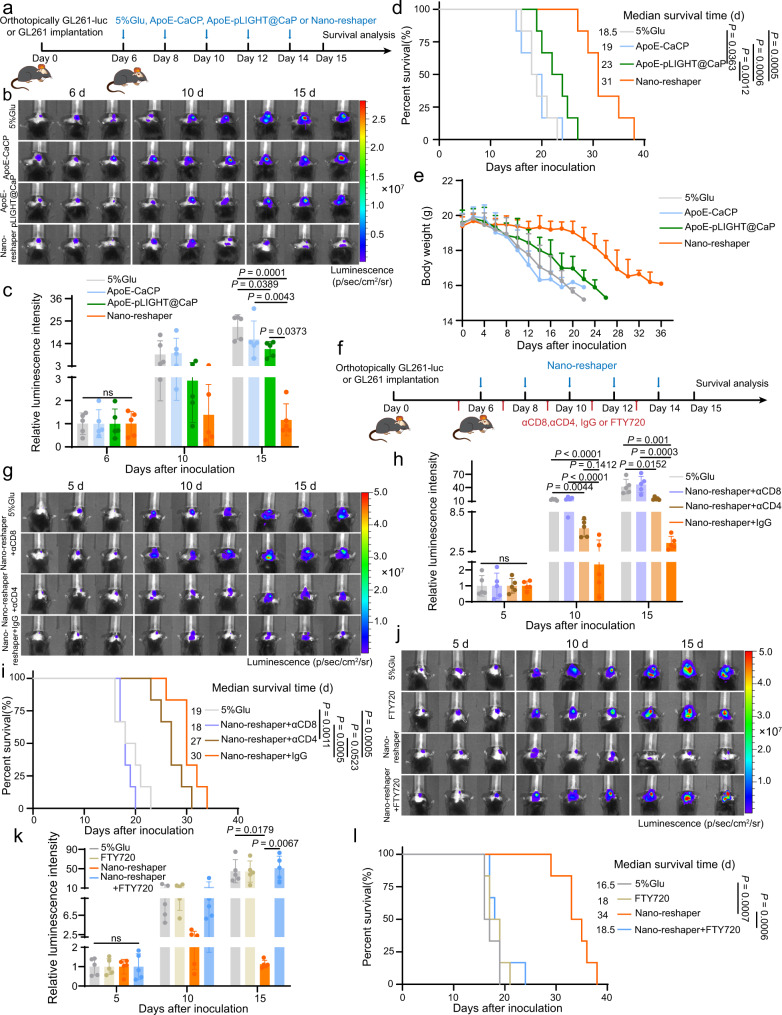
Nano-reshaper inhibited tumor growth and prolonged the survival of mice bearing intracranial GL261 GBM. **a** Orthotopic tumor implantation and treatment scheme for ApoE-CaCP, ApoE-pLIGHT@CaP and Nano-reshaper therapy. **b** Representative in vivo bioluminescence imaging of mice bearing intracranial GL261-luc GBM after treatment with ApoE-CaCP, ApoE-pLIGHT@CaP, or Nano-reshaper on days 6, 10, and 15 post tumor inoculation. **c** Semi-quantitative results of tumor burden by bioluminescence intensity shown in **b** (*n* = 5 mice). **d** Kaplan–Meier survival curve of intracranial GL261 GBM-bearing mice after various treatment (*n* = 6 mice). **e** Changes in the body weight of mice bearing intracranial GL261 GBM during the therapeutic period (*n* = 6 mice). **f** Scheme for CD4^+^ or CD8^+^ T-cell depletion and FTY720 administration. Mice were *i.p*. injected with αCD4 (10 mg/kg), αCD8 (10 mg/kg) or FTY720 (1.5 mg/kg) on days 5, 7, 9, 11, and 13 post tumor inoculation. **g** Representative in vivo bioluminescence imaging of mice bearing intracranial GL261-luc GBM after depletion of CD4^+^ or CD8^+^ T-cell for Nano-reshaper treatment. **h** Semi-quantitative results of tumor burden by bioluminescence intensity shown in **g** (*n* = 5 mice). **i** Kaplan–Meier survival curve of intracranial GL261 GBM-bearing mice in CD4^+^ or CD8^+^ T-cell depletion study (*n* = 6 mice). **j** Representative in vivo bioluminescence imaging of mice bearing intracranial GL261-luc GBM after reducing peripheral T cells by FTY720 for Nano-reshaper treatment. **k** Semi-quantitative results of tumor burden by bioluminescence intensity shown in **j** (*n* = 5 mice). **l** Kaplan–Meier survival curve of intracranial GL261 GBM-bearing mice in FTY720 administration study (*n* = 6 mice). Data were shown as mean ± SD. Error bars represent SD. Significant differences were evaluated in **c**, **h**, **k** using one-way ANOVA with Tukey multiple comparisons post-test, in **d**, **i**, **l** using Kaplan–Meier analysis with log-rank test. Source data are provided as a Source Data file.

Nano-reshaper efficiently reprogrammed systemic and local immune function in vivo, we subsequently evaluated its anti-GBM efficacy in an orthotopic GL261-luc model (Fig. 7a). Nano-reshaper impressively inhibited rapid tumor growth compared to 5% Glu group, as proved by the lower tumorous luciferase intensity, while either ApoE-CaCP or ApoE-pLIGHT@CaP showed a partial inhibitory effect (Fig. 7b, c). Nano-reshaper-treated mice survived much longer (median of 31 days) than ApoE-CaCP-treated mice (19 days) or ApoE-pLIGHT@CaP-treated mice (23 days) (Fig. 7d). Meanwhile, no significant body weight loss was observed in Nano-reshaper group during treatment, which also indicated the effective antitumor ability (Fig. 7e). The involvement of specific type of T cells for the Nano-reshaper mediated tumor control was further confirmed through the depletion study, in which anti-CD8 antibody (αCD8) significantly compromised the therapeutic efficacy of Nano-reshaper compared to the IgG control, but little effect was observed on anti-CD4 antibody (αCD4) (Fig. 7f–i). This proved that CD8^+^ T cells were essential for the Nano-reshaper mediated tumor control. To validate whether the anti-tumor effect of Nano-reshaper relied on peripheral T cells, we utilized fingolimod (FTY720) to block peripheral T cells migration into tumors^56^. As anticipated, the majority of T cells in peripheral blood disappeared after FTY720 treatment (Supplementary Fig. 31). The therapeutic efficacy of Nano-reshaper was greatly abrogated by the simultaneous treatment with FTY720 (Fig. 7j–l). This demonstrated that persistent peripheral T-cell infiltration contributed to the tumor control of Nano-reshaper. Overall, these data suggested the capacity of Nano-reshaper to inhibit GBM growth and CD8^+^ T cells were the key immune cell subpopulation that primarily drove the antitumor activity, and peripheral T-cell infiltration was essential for this therapeutic strategy to maintain the tumor control.

### Nano-reshaper sensitized GBM to αPD-1 therapy and protected survivors against GBM rechallenge

**Fig. 8:**
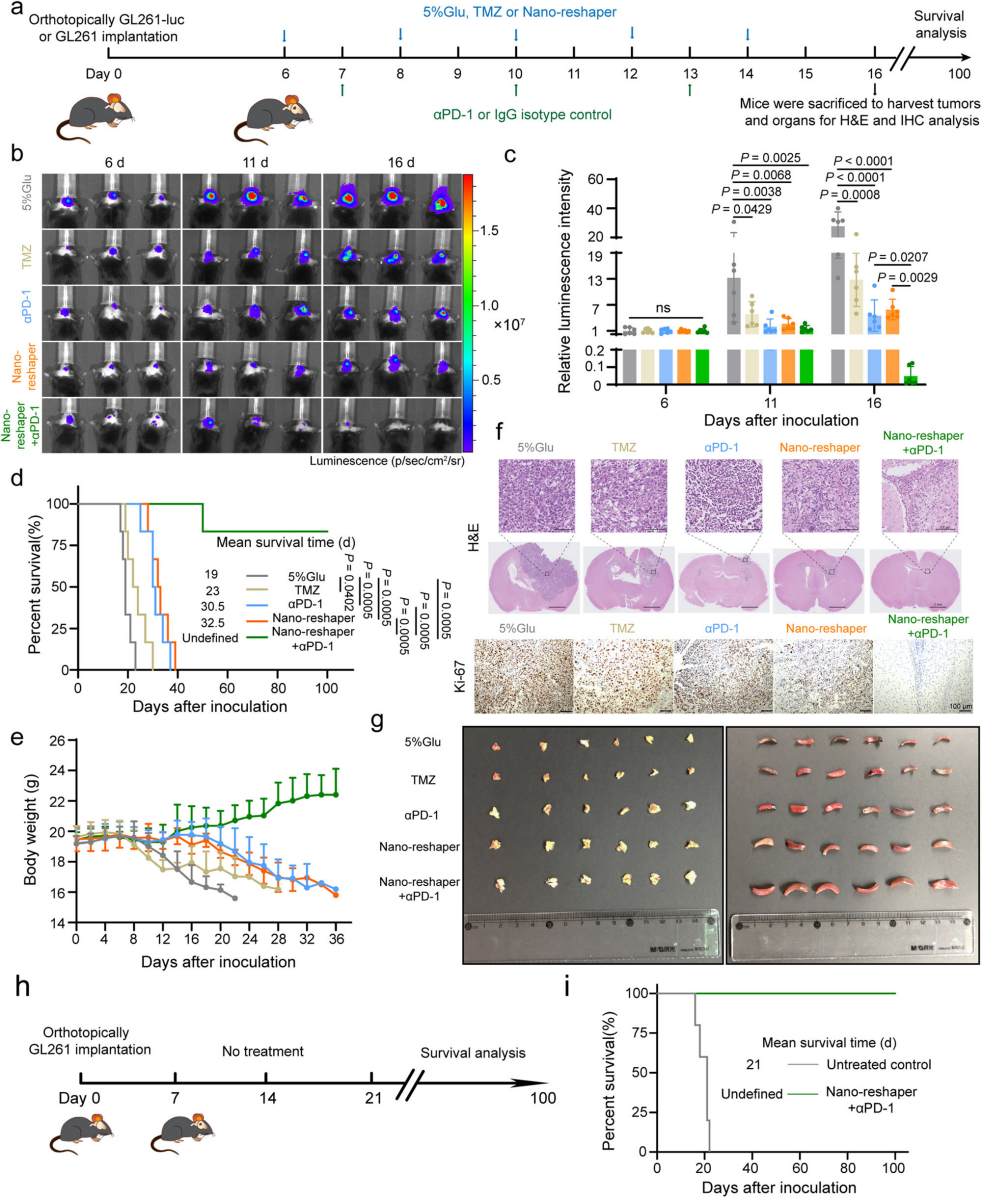
Nano-reshaper sensitized GBM to αPD-1 therapy and protected survivors against GBM rechallenge. **a** A scheme to illustrate construction of intracranial GL261-luc or GL261 GBM and administration schedule of various formulations. **b** Representative in vivo bioluminescent images of mice bearing intracranial GL261-luc GBM-receiving various treatments on days 6, 11, and 16 post tumor inoculation. **c** Tumor burden quantification 6, 11, and 16 days after tumor inoculation (*n* = 6 mice). **d** Kaplan–Meier survival curve of intracranial GL261 GBM-bearing mice receiving various treatments (*n* = 6 mice). **e** Changes in the body weight of mice bearing intracranial GL261 GBM during the therapeutic period (*n* = 6 mice). **f** H&E and Ki-67 staining of the intracranial GL261 GBM sections on day 16 post tumor inoculation. The experiments were repeated three times independently. **g** Images of the dissected thymuses (left side) and spleens (right side) after treatment with various formulations. **h** Schedule and timeline of the long-term survivors for re-challenging survival study. No additional treatment was provided after the tumor implantation. **i** Kaplan–Meier survival curve indicates all re-challenged survivors achieved a second long-term survival benefit without any therapeutic interventions (*n* = 5 mice). Data were shown as mean ± SD. Error bars represent SD. Significant differences were evaluated in c using one-way ANOVA with Tukey multiple comparisons post-test, in **d** and **i** using Kaplan–Meier analysis with log-rank test. Ns, not significant. Source data are provided as a Source Data file.

As Nano-reshaper increased the number of tumor-specific T cells in tumor bed, we further explored the therapeutic effects of Nano-reshaper when combined with αPD-1 using GL261 orthotopic model (Fig. 8a). Tumor development was considerably slowed following treatment with TMZ, αPD-1, Nano-reshaper and Nano-reshaper + αPD-1, as evidenced by lower tumorous luciferase intensity (Fig. 8b, c), H&E staining and decreased Ki-67 expression (Fig. 8f and Supplementary Fig. 32). Compared to the other groups, Nano-reshaper + αPD-1 group achieved a superior survival benefit (Fig. 8d). Five mice survived for a lengthy period of time following treatment with Nano-reshaper + αPD-1. No substantial weight reduction was found in Nano-reshaper + αPD-1 group (Fig. 8e). Similar therapeutic efficacy was also verified on G422 orthotopic model, proving the synergistic action of Nano-reshaper and αPD-1 (Supplementary Fig. 33). Additionally, the thymus and spleen indexes were considerably elevated in the Nano-reshaper + αPD-1 group (Fig. 8g, Supplementary Fig. 34a, b), showing that systemic immune function was strengthened. H&E staining demonstrated that the thymic medulla and spleen white pulp regions were both enlarged in mice receiving Nano-reshaper + αPD-1, indicating lymphocyte proliferation in thymuses and spleens (Supplementary Fig. 34c).

Due to the aggressive and infiltrative characteristics of GBM, the current standard-of-care methods fail to prevent recurrence. To investigate whether these long-term survivors after treatment of Nano-reshaper + αPD-1 induced immunological memory to prevent GBM recurrence, we continued to implant GL261 cells into the contralateral hemisphere of mice, naïve mice implanted with GL261 cells were established as the control group, and animals in both groups received no further treatment (Fig. 8h). Notably, all re-challenged mice in Nano-reshaper + αPD-1 group demonstrated a second long-term survival, whereas mice in control group perished rapidly and had a median survival duration of 21 days (Fig. 8i). Meanwhile, on day 16 following tumor cell implantation, we examined the T cell memory response in blood through flow cytometry analysis, finding that mice treated with Nano-reshaper + αPD-1 exhibited an increased percentage of effector memory T cells (T_EM_) and central memory T cells (T_CM_) (Supplementary Fig. 34d–f), accounting for the resistance of tumor cell rechallenge.

### Nano-reshaper exhibited satisfactory safety in vivo

Along with anticancer effectiveness, safety is critical. On healthy C57BL/6 mice, the potential toxicity of various therapies was evaluated using the same delivery schedule as described above. White blood cells, including lymphocytes, monocytes, and neutrophils, increased to a certain level following treatment with αPD-1, Nano-reshaper, or Nano-reshaper + αPD-1 (Supplementary Fig. 35b), but all other hematological and serum biochemical parameters remained within the normal range (Supplementary Fig. 35a). Additionally, no discernible structure changes in the hearts, livers, spleens, lungs, kidneys, brains, or thymuses were found (Supplementary Fig. 36), confirming the safety of the Nano-reshaper + αPD-1 therapy.

## Discussion

Despite massive research efforts devoted to GBM, it remains incurable with the current conventional treatments due to the presence of BBB, infiltrative, heterogeneous and hypoxic pathophysiology^4^. With the advancement of immunotherapy, there is growing interest in leveraging the immune system against GBM. At present, immunotherapeutic approaches developed for GBM include immune checkpoint inhibitors, chimeric antigen receptor T cell (CAR-T) therapy, vaccines, oncolytic virus and cytokines therapy, which mainly rely on T cells to exert anti-GBM activity. However, the multiple and unique mechanisms of immunosuppression in GBM disrupt immune function and impede the infiltration of sufficient effector T cells into tumor bed. One major characteristic feature is systemic immunosuppression, constituting one of the most fundamental challenge in the immunotherapy, which might be the primary cause why conventional T-cell based immunotherapies have shown little efficacy to date^20,22^. Unfortunately, the exact underlying mechanisms behind this immunosuppression remain largely unknown^22^. Local immunosuppressive TME is another hurdle, whose components and functions are well understood up to now, and it mainly acts as a vital facilitator of immune escape and limits the efficacy of immunotherapy^6,57^. Overall, GBM establish immunosuppression both systemically and locally, impairing the efficacy of immunotherapy. Therefore, multiple disparate modes of immune-oriented treatment are requisite to overcome systemic and local immunosuppression to get rid of GBM.

In this work, we show that reprogrammed systemic and local immune function empower immunotherapy against poorly immunogenic GBM based on its pathological features. In the preliminary study, we discovered that CBD facilitated T-cell proliferation in the presence of GBM-associated systemic immunosuppressive factors (Supplementary Fig. 18) and promoted APCs activation (Supplementary Fig. 24), guiding us to explore its potential for alleviating systemic immunosuppression in GBM. LIGHT, which could regulate TME to facilitate T-cell infiltration through vascular normalization, induction of lymphoid chemokine and HEVs, was applied to overcome local immune dysfunction^35,36^. To simultaneously deliver LIGHT and CBD, we constructed Nano-reshaper. As expected, Nano-reshaper altered both the systemic and local immune responses in GL261-bearing mice (Figs. 4–6). Additionally, in vivo experiments demonstrated that Nano-reshaper substantially suppressed GBM development and increased the survival duration of tumor-bearing mice, which primarily relied on T cells to drive the antitumor efficacy (Fig. 7). Recent research on PD-1 checkpoint-based immunotherapy has highlighted the critical need of T-cell infiltration in tumor bed for optimal antitumor effectiveness^35,58^. As a result, we examined the synergistic impact of Nano-reshaper and αPD-1 in the treatment of GBM. The results indicated that when Nano-reshaper was combined with αPD-1 therapy, 83.3% of tumor-bearing mice gained long-term survival benefit, and immunological memory was efficiently established to prevent recurrence without producing significant adverse effects (Fig. 8 and Supplementary Figs. 34–36).

To date, several strategies can be applied to rectify systemic functional deficits caused by immunosuppression. The first is passive therapy, in which patients are infused with CAR-T (NCT03726515; NCT03347097) to exert therapeutic T-cell role by directly increasing the number of systemic T cells rather than substantially boosting systemic immune function. The major problem of CAR-T therapy is that unlike leukemia and lymphoma the bulk of immunologically-cold tumors, such as GBM, are highly heterogeneous, implying that the infused CAR-T may not eliminate all tumor cells^6^. The second one is the application of T-cell immunomodulators such as IL-2, IL-7, and IL-12 cytokines, which can regulate T-cell expansion, survival and function and possess strong ability to enhance systemic immune function^59^. Regrettably, the lack of drug-like properties and extensive systemic effects can result in substantial toxicity, limiting its clinical efficacy^37^. Thus, it is critical to select suitable medicines to ameliorate immunological aberrations to enable legitimate anticancer immune responses against GBM.

Notably, we used CBD in our developed Nano-reshaper to restore thymus function, increase naive T cell export, and boost systemic immunity in GBM-bearing mice (Supplementary Figs. 18–20). CBD is a well-tolerated and clinically safe drug that has previously been approved by FDA for human use under the brand name Epidiolex®. We found that though systemic immune function was enhanced to increase circulated T cells after the treatment of ApoE-CaCP (Supplementary Figs. 19 and 20a, b), effector T cells were not significantly increased in brain tumors (Supplementary Fig. 20c, d), indicating the existence of disrupted local immune function. Successful immunotherapy requires sufficient effector T cells in tumor parenchyma, but immunosuppressive TME retards T-cell infiltration, which also weakens antitumor efficacy^10,60^. This might explain why ApoE-CaCP showed negligible antitumor efficacy in orthotopic GL261-luc model (Supplementary Fig. 19a–c), implying that enhancing systemic immune function merely was insufficient to achieve effective treatment. In accordance with earlier studies, intracerebral or intraventricular administration of CAR-T cells results in prolonged survival, whereas peripheral administration had no effect^7,61,62^. It is worth noting that the increased effectiveness has been reported in studies in which modified T cells were attracted to infiltrate solid tumors by the overexpressed chemokines in TME^60,63^. In light of this, we incorporated a plasmid encoding LIGHT into ApoE-CaCP to create Nano-reshaper, with the goal of overcoming local immunosuppression and promoting effector T cells recruitment. The vast majority of immune cells in the TME of GBM are myeloid-lineage cells that act as APCs, namely macrophages, microglia, and DCs^7,64^. However, these myeloid cells contribute to immunosuppression rather than playing the role of APCs in initiating an immune response^65^. Intriguingly, our results showed that CBD activated macropinocytosis pathway of APCs to capture antigens and improved antigen cross-presentation (Supplementary Figs. 24–26). Thus, on the one hand, the design of the Nano-reshaper takes use of the effect of CBD on the phagocytosis ability of APCs in order to boost the efficacy of plasmid transfection in GBM (Fig. 2g and Supplementary Fig. 16). On the other hand, administration of CBD to tumors can work synergistically with LIGHT to enhance local immune activity, as demonstrated by the findings that Nano-reshaper optimally increased APC activation and reprogrammed local immune function (Figs. 5 and 6i).

Rarely has research emphasized the importance of systemic immunosuppression in the treatment of GBM. Indeed, modulating systemic immunosuppression to ameliorate lymphopenia is critical for optimizing the response to T-cell activating or mobilizing immunotherapies. Here we found that CBD increased the number of systemic T cells in an orthotopic GBM model, indicating that CBD may also have the capacity to reduce lymphopenia in clinical GBM patients that is aggravated by standard-of-care and steroid therapy. While identifying the GBM Achilles’ heel is a difficult endeavor, the current study demonstrating the value of systemic immunosuppression in GBM immunotherapy and providing possible therapeutic agents.

To realize clinical translation, several limitations of the study should be noted. First, while the response rate to T-cell immunotherapy was enhanced in the current GL261 and G422 models, the effects must be confirmed in the clinic since GBM has a low mutation rate compared to preclinical models^7,10^. Second, Nano-reshaper increased the number of lymphocytes in the blood of tumor-free mice, showing that it can strengthen systemic immune function regardless of tumor load. While the mechanism by which CBD exerted its immune-modulatory impact in vitro and in vivo remains largely unknown, the current research demonstrated the relevance of CBD in the field of immunotherapy. Third, while we concentrated on improving T cells, it was possible that other immune cells were also impacted by Nano-reshaper therapy.

In summary, considering the unique pathological features and current treatment status of GBM in clinical settings, we developed a nanostructure named Nano-reshaper for co-delivering CBD and LIGHT to reprogram systemic and local immune function to empower immunotherapy against GBM. Nano-reshaper improved anti-GBM immune response by surmounting both systemic and local immunosuppression, increased the number of systemic T cells and promoted the infiltration of effector T cells in GBM. The synergistic effect of Nano-reshaper with αPD-1 proves its potential in clinical application and the value of this strategy to empower the other T cell-based immunotherapies such as vaccines and oncolytic virus against GBM. Furthermore, this platform technology that reprograms immune function both systemically and locally may be applicable for a wide range of immunologically-cold tumors with similar clinical immunosuppressive characteristics.

## Methods

### Ethical regulations

All the animal experiments were performed in accordance with the guidelines evaluated and approved by Institutional Animal Care and Use Committee (IACUC), Fudan University School of Pharmacy (Ethical approval number: 2018-03-YJ-CJ-01).

### Materials

1,2-dioleoyl-sn-glycero-3-phosphate (DOPA) and (2,3-dioleoyloxy-propyl)-trimethylammonium (DOTAP) were purchased from Avanti Polar Lipids (Alabaster, AL, USA). N-(methoxy polyethylene glycol_2000_)-1,2-distearoyl-sn-glycero-3-phosphoethanolamine (mPEG_2000_-DSPE) and Mal-PEG_2000_-DSPE and were obtained from Xi’an Ruixi biotech (Xi’an, China). mPEG-PLA was kindly provided by East China University of Science and Technology. Apolipoprotein E peptide (141–150)_2_ with a tryptophan to monitor fluorescence and a cysteine to couple Mal-PEG_2000_-DSPE (ApoE, sequence: (CWG-(LRKLRKRLLR)_2_-NH_2_, 95%) and octaarginine peptide (mc-CR8C) were purchased from GL Biochem Ltd. (Shanghai, China). Cholesterol (CH) was provided by Shanghai Advanced Vehicle Technology Pharmaceutical, Ltd. (Shanghai, China). 1,1′-dioctadecyl-3,3,3’,3’-tetramethylindotricarbocyanine iodide (DiR), 1,1′-dioctadecyl-3,3,3′,3′-tetramethylindocarbocyanine perchlorate (DiI), 1,1′-dioctadecyl-3,3,3′,3′-tetramethylindodicarbocyanine perchlorate (DiD), 5-(and 6)-Carboxyfluorescein diacetate, succinimidyl ester (CFDA SE) and agar (70101ES76) were purchased from Shanghai yeasen biotech (Shanghai, China). IGEPAL® CO-520, Hoechst 33258, 3-[4,5-dimethylthiazol-2-yl]-2,5-diphenyl tetrazolium bromide (MTT) and cou6 were purchased from Sigma-Aldrich (St. Louis, MO, USA). YOYO-1 was obtained from Invitrogen (California, USA). d-luciferin potassium was bought from PerkinElmer Inc. (Waltham, MA, USA). CBD was purchased from Biopurify Co., Ltd (Chengdu, China). Yeast extract (LP0021) and tryptone (LP0042) were obtained from OXOID (Hants, UK). TMZ, OVA and FTY720 were obtained from Aladdin Co., Ltd (Shanghai, China). αPD-1 (clone: RMP1-14), IgG isotype (2A3, BP0089), αCD8 (clone: 53-6.72), and αCD4 (clone: GK1.5) were acquired from BioXcell (New Hampshire, USA). All the other chemical reagents and solvents were purchased from Sinopharm Chemical Reagent Co., Ltd (Shanghai, China) unless specified.

### Cell lines and animals

The original GL261 cells were kindly provided by Dr. Jianhai Jiang from Fudan University School of Medicine (Shanghai, China). Murine GBM G422 cells were kindly provided by Dr. Changyou Zhan from Fudan University School of Medicine (Shanghai, China). Murine GBM GL261 cells stably expressing firefly luciferase (luc) or green fluorescent protein (GFP) were obtained through transducing GL261 cells with lentivirus vectors (Hanyinbt, Shanghai) having luc or GFP gene along with puromycin resistance gene. Transfected GL261-luc and GL261-GFP cells were selected with puromycin (1 μg/mL). RAW264.7, BV2, DC2.4, and bEnd.3 cells were purchased from Chinese Academy of Science Cell Bank (Shanghai, China). GL261, GL261-luc, GL261-GFP, G422, RAW264.7, BV2 cells and bEnd.3 were cultivated in Dulbecco’s modified Eagle’s medium (DMEM, high glucose, Hyclone) supplemented with 10% fetal bovine serum, 1% penicillin-streptomycin solution. DC2.4 cells were cultivated in RPMI-1640 (Hyclone) supplemented with 10% fetal bovine serum, 1% penicillin-streptomycin solution. The culture condition is 37 °C and 5% CO_2_ in a humidified atmosphere. Bone marrow derived dendritic cells (BMDCs) were obtained by a classical method and cultured using RPMI-1640 medium containing GM-CSF (20 ng/mL)^66^. Male C57BL/6 mice (6–10 weeks old, 18–22 g) or Kunming mice (3–4 weeks old, 18–22 g) were purchased from SLAC Animal Ltd. (Shanghai, China) and raised in a pathogen-free facility with a 12 h light and dark cycle at 18–23 °C and 40–60% humidity and had free access to food and water. In terms of animal experiment studies, male mice were chosen. Male mice are less likely to die during the establishment of orthotopic GBM models based on our historical experience^67,68^. This can reduce accidental death and help to ensure the objectivity of the studies.

### Synthesis and characterization of CP

In brief, 600 μM CBD was dissolved in 10.0 mL anhydrous tetrahydrofuran (THF) along with 500 μL triethylamine (TEA). Then 180 μL phosphorus oxychloride (POCl_3_) in 10 mL anhydrous THF was added dropwise under fast stirring at 0 °C over 30 min. The reaction was kept for another 6 h under nitrogen at room temperature with the formation of a precipitate. Then, distilled water was added to the reaction until the precipitate disappeared. After removing THF by evaporation, 5% NaOH solution was added until pH reached 10. The unreacted CBD in solution was extracted with ethyl acetate. At last, CP was precipitated by adding 6 M HCl to the solution. Then anhydrous ethanol was added to the obtained CP mixture, mixed thoroughly and centrifugated to remove the precipitate NaCl. The anhydrous ethanol was removed by evaporation at 40 °C, the residues were dissolved in tert-Butyl alcohol and lyophilized to obtain purified CP.

### Release study

The drug release was performed in PBS (pH 7.4, 6.5 and 5.0) at 37 °C on a rotating shaker. Nano-reshaper was added into dialysis bags and immersed in different pH medium. Samples were withdrawn at 0.5, 1, 2, 4, 6, 8, 12, 24, 36, and 48 h and an equal volume of fresh media was added at the same time. The amount of released CP was determined by high performance liquid chromatography (HPLC) at the detector wavelength of 224 nm. To assess the release profile of plasmid, we used YOYO-1 to label the plasmids and the amount of released plasmids was determined via fluorescence spectrometry (*λ*_ex_ = 491 nm, *λ*_em_ = 509 nm).

### CP conversion to CBD assay

The conversion of CP was validated in GL261, RAW264.7, BV2, and DC2.4 cells. Nano-reshaper containing 50 μg CP was added to GL261, RAW264.7, BV2, and DC2.4 cells. After 4 h incubation, the cells and medium were collected, subjected to ultrasonication under ice bath and lyophilization. Then 500 μL methanol was added, and the CBD was detected by HPLC at the detector wavelength of 220 nm.

### Synthesis and characterization of ApoE-PEG_2000_-DSPE

The GBM targeting material ApoE-PEG_2000_-DSPE was synthesized by conjugating ApoE to mal-PEG_2000_-DSPE via Michael addition. In brief, Mal-PEG_2000_-DSPE (9.7 mg, 3.3 μM) was dissolved in 2.0 mL DMSO and ApoE (12 mg, 3.96 μM) was dropped to this solution under magnetic stirring with the protection of nitrogen. The reaction was proceeded for 12 h at room temperature. After that, the solution was dialyzed (MWCO: 3500) against DMSO for 24 h to remove the unreacted ApoE and Mal-PEG_2000_-DSPE and then distilled water for 24 h to remove DMSO. The final product was obtained by lyophilizing the retentate. The purity of the obtained product was calculated through the fluorescence intensity of ApoE peptide (*λ*_ex_ = 280 nm, λ_em_ = 350 nm) measured by a fluorescence spectrophotometer. The product ApoE-PEG_2000_-DSPE was analyzed by ^1^H-NMR (Bruker 600-MHz).

### Preparation and characterization of Nano-reshaper

The inner cores of Nano-reshaper were prepared by water-in-oil microemulsions method as described previously with some modification^41^. In brief, two separate microemulsions (20 mL each) made of cyclohexane/Igepal® CO-520 (7:3, *v*/*v*) were prepared with constant stirring. A pDNA (180 μg, 2.0 mg/mL) solution was prepared and mixed with 300 μL 2.5 M CaCl_2_ solution. To this solution, 25 μL of 8 mg/mL mc-CR8C was added and dropped to the microemulsion to form the calcium part. A CP solution (200 μL, 12.5 mM) and Na_2_HPO_4_ (100 μL, 12.5 mM) was also prepared and added to the other microemulsion. This microemulsion containing CP was mixed with the other microemulsion containing DNA/mc-CR8C/CaCl_2_. The mixed microemulsion was allowed to stir for 5 min before adding 300 μL DOPA (20 mM) in chloroform. After the addition of DOPA, the microemulsion was left to stir for another 45 min. An equal volume of 100% ethanol (40 mL) was added and the mixture was centrifuged at 12,500×*g* for 20 min at 4 °C. The supernatant was discarded and the precipitates were washed twice with 100% ethanol to remove the cyclohexane and Igepal® CO-520. The precipitates were suspended in chloroform and store at −20 °C for further use.

To obtain the final Nano-reshaper, 10 mg cores and 350 μL 20 mM CH, 350 μL 20 mM DOTAP, 250 μL 20 mM mPEG_2000_-DSPE and 50 μL 20 mM ApoE-PEG_2000_-DSPE dissolved in chloroform were mixed and evaporated in vacuum to obtain a thin lipids film. 5% Glu solution was used to rehydrate the lipids film followed by sonication to obtain the final formulation. The DiI, DiD, and DiR-labeled CaP nanoparticles were prepared with the same procedure by adding 1% DiI, DiD, and DiR to the lipids.

The particle size and *zeta* potential of Nano-reshaper were detected by a Malvern ZetaSizer Nano series (Westborough, MA). TEM images of Nano-reshaper were obtained using a TEM (TEM-1400 Plus Electron Microscope, Leica, Germany). The LE and EE of CP were determined by HPLC at the detector wavelength of 224 nm. The LE and EE of plasmid were determined according to the previous report^69^. The cores were collected and lysed by pH 4.0 acetic acid buffer, and the peptide/DNA complex was dissociated in protease K solution at 37 °C for 1 h. After that Hoechst 33258 nucleic acid stain was added and measured by fluorescence spectrometry. DNA encapsulation efficiency was calculated by using a standard curve obtained via blank cores mixed with known concentrations of peptide/pDNA complexes.

### Stability of Nano-reshaper

The long-term storage stability of Nano-reshaper was evaluated over a period of three months at 4 ± 2 °C. The dilution stability of Nano-reshaper was assessed in 5% Glu and 0.9% NaCl for one week. In brief, 0.1 mL Nano-reshaper suspensions were diluted in 4.9 mL 5% Glu and 0.9% NaCl, and stored at 4 ± 2 °C. The particle size was measured by DLS analysis.

### Preparation of cou6-labeled mPEG-PLA nanoparticles

To evaluate the phagocytic capacity of APCs, cou6-labeled mPEG-PLA nanoparticles were used as the fluorescent marker. Cou6-labeled mPEG-PLA nanoparticles was prepared by emulsion/solvent evaporation as described previously^38,70^. In brief, 0.1 mg cou6 and 10 mg mPEG-PLA were dissolved in 1.0 mL dichloromethane (DCM). 2.0 mL 1% sodium cholate solution was slowly added to the surface of the DCM solution and subjected to ultrasonication under ice bath. The obtained emulsion was dispersed in 8.0 mL 0.5% sodium cholate solution. After removing DCM by evaporation, the nanoparticle suspension was centrifuged at 20,000×*g* for 1.0 h and resuspended in 5% Glu solution. At last, a 1.5 × 20 cm sepharose CL-4B column was used to remove the free cou6.

### Investigation of phagocytic capacity on RAW264.7, BV2 and DC2.4 cells

RAW264.7, BV2, and DC2.4 cells (5000 cells/well) were planted in 96-well plates and cultured 24 h for attachment. Then the medium was replaced with DMEM containing different concentrations of free CBD. The medium was discarded after incubation for 24 h, then cou6-labeled mPEG-PLA nanoparticles (at the cou6 concentration of 50 ng/mL) dispersed in DMEM was added and incubated for another 2 h at 37 °C. After that, RAW264.7 and BV2 cells were washed with PBS, fixed with 4% paraformaldehyde and stained with 2 μg/mL Hoechst 33258, and detected by a KineticScan® HCS Reader (Version 3.1, Cellomics Inc., Pittsburgh, PA, USA). For DC2.4 cells, the cells were collected, washed with PBS and detected by flow cytometry (Beckman, coulter).

Endocytosis inhibition experiments were performed on RAW264.7, BV2 and DC2.4 cells to explore the mechanism of the enhanced uptake of cou6-labeled mPEG-PLA nanoparticles after treatment with CBD. RAW264.7, BV2 and DC2.4 cells (5000 cells/well) were planted in 96-well plates and cultured 24 h for attachment. Then the medium was replaced with DMEM containing 8 μM CBD and incubated for 24 h. After that, the medium was replaced with different endocytic inhibitors including 200 μM genistein, 10 mM filipin, 5 mM amiloride, 20 μM nocodazole, 10 μM chlorpromazine, 3 μM cytochalasin d, 200 nM monensin, 5 mM NaN_3_ + DOG, 18 μM brefeldin A, 10 μM colchicine, for 1 h at 37 °C. Then, cou6-labeled mPEG-PLA nanoparticles were added (at the cou6 concentration of 50 ng/mL) and incubated for another 2 h. Then the cells were washed with PBS, fixed with 4% paraformaldehyde, stained with Hoechst 33258 and detected by a KineticScan® HCS Reader (version 3.1, Cellomics Inc., Pittsburgh, PA, USA).

### Fluo-3 AM Ca^2+^ imaging

DC2.4 cells (2 × 10^4^) were seeded in 24-well plates and incubated for 24 h. Then the medium was replaced with DMEM containing 8 μM free CBD and incubated for another 24 h. After that, the cells were washes with PBS and incubated with 2.5 μM Fluo-3 AM (Macklin, Biochemical Co., Ltd, China) for 30 min in the dark. The cells were then washed with PBS again and observed under fluorescence microscope (Leica DMI 4000B, Germany). The quantitation of Ca^2+^ imaging signals was determined using Image J software.

### T-cell proliferation assay in vitro

Spleen cells were obtained from naïve unmanipulated male C57BL/6 mice (6–10 weeks old, 18–22 g) and suspended in HBSS buffer (Beyotime Biotechnology Co., Ltd, Nantong, China) at 1 × 10^7^ cells/mL. CFDA SE (5 mM) was added at a final concentration of 2.5 μM followed by a 5 min incubation at 37 °C. Then the cells were washed with complete RPMI-1640 medium and suspended in complete RPMI-1640 medium. The cells were implanted at 2 × 10^6^ cell/well in a 24-well plate. αCD3/CD28 (Biolegend) were added at 500 ng/mL in each well. To these cultures, we added 50 μL serum obtained from experimental mice, along with various concentrations of CBD. The final volume of each well in 24-well plates was 1 mL. The cells were cultured for 72 h. Then the cells were washed with PBS, incubated with anti-CD8α and anti-CD4 antibody (Supplementary Table 2) for 45 min at 4 °C. After that, the cells were washed with PBS and the CFSE dilution was determined using flow cytometry (Beckman, coulter) by gating on CD8^+^ or CD4^+^ cells.

### Cytotoxicity assay

MTT assay was used to assess the cytotoxicity of CBD in RAW264.7, BV2 and DC2.4 cells. Briefly, RAW264.7, BV2 and DC2.4 cells (5000 cells/well) were planted in 96-well plates and cultured 24 h for attachment. Then the medium was replaced with DMEM containing different concentrations of free CBD. Free DMEM without CBD was used as the control group. After incubation for 24 h, MTT solution (5 mg/mL) was added and incubated with cells for another 4 h at 37 °C, then 200 μL DMSO was added to dissolve the produced formazan crystals and the cells were detected at 490 nm using the microplate reader (Thermos Multiskan MK3, USA).

### In vitro cell uptake study

GL261 and bEnd.3 (1 × 10^4^) were seeded in 96-well plates and cultured 24 h for attachment. Different ApoE densities of DiI-labeled Nano-reshaper were added to each dish and incubated for 0.5, 1, 2, and 4 h, respectively. After that, the cells were washed with PBS and fixed by 4% paraformaldehyde. Then the nuclei were stained with 2 μg/mL Hoechst 33258 for 10 min and detected by a KineticScan® HCS Reader (version 3.1, Cellomics Inc., Pittsburgh, PA, USA).

### In vitro transfection assay

To examine the transfection efficiency, flow cytometry and confocal assay were performed. GL261 cells were cultured until 80–90% confluent in 6-well plates. Then EGFP-Nano-reshaper loaded with plasmid (2 µg) were added in the presence of Opti-MEM. The medium was replaced with DMEM 6 h after the transfection and incubated for another 48 h. The non-EGFP coding vector plasmid-loaded nanoparticle (ApoE-pVector@CaCP) was set as the negative control (NC), and the commercial reagent, Hieff Trans^TM^ Liposomal Transfection Reagent was set as a positive control following the manufacturer’s protocol. After the imaging, the expression of EGFP was measured via flow cytometry and observed by confocal laser scanning microscopy (CLSM, LSM710, Leica, Germany).

### In vivo gene expression tracking assay

In total two doses of Nano-reshaper (1.5 mg/kg plasmid per mouse, *n* = 3 for each group) were *i.v*. injected into mice bearing intracranial GL261 GBM every two days. Mice were killed one day after the second injection. The brains were collected and performed with frozen sections, and the slices were stained with fluorescently labeled anti-mouse CD11c and F4/80 antibody, or primary rabbit anti-mouse Trp-2 and CD31 antibodies followed by secondary Alexa Flour 647-labeled goat anti-rabbit IgG antibodies (Supplementary Table 2). Then the sections were stained with Hoechst 33258 and analyzed for cellular positive expression by confocal laser scanning microscopy (CLSM, LSM710, Leica, Germany). Quantification of EGFP signals was analyzed using Image J software. Quantification of LIGHT expression in major organs and serums were performed using a mouse His-tag ELISA kit (AKR-130, Cell Biolabs).

### In vitro BBB transcytosis assay

The BBB transcytosis assay of Nano-reshaper was first evaluated by an in vitro BBB model as previously reported. In general, bEnd.3 cells (2 × 10^4^) were seeded on polycarbonate 24-well transwell chambers (FALCON cell culture insert, Becton Dickinson Labware, USA). The transendothelial electrical resistance (TEER) of bEnd.3 was determined by an epithelial voltmeter (Millicell-RES, Millipore, USA). The TEER of this model over 200 Ω·cm^2^ was qualified for the transcytosis assay. DiI-labeled Nano-reshaper and pLIGHT@CaCP (at the DiI concentration of 1 μg/mL) were added to the apical chambers. The bEnd.3 cells in apical chambers was treated with free ApoE (100 μg/mL) for 1.0 h before adding DiI-labeled Nano-reshaper for the competition experiment. After 4 h incubation, the medium was taken from the bottom chambers and the concentration of DiI-labeled nanoparticles was determined by a fluorescence spectrometer (Cary Eclipse, Agilent, USA). Transport ratio (%) of CaP nanoparticles was calculated as the amount of DiI-labeled CaP nanoparticle across the monolayer to that of original amount.

### In vivo brain distribution assay

To investigate the brain distribution of Nano-reshaper in vivo, DiR-labeled Nano-reshaper were *i.v*. injected into three male C57BL/6 mice (6–10 weeks old, 18–22 g). Mice were euthanized at 24 h after the injection and hearts were perfused with 0.9% saline and 4% paraformaldehyde sequentially. Then the brains were collected, and the images were obtained and semi-quantitatively measured by an IVIS imaging system (Caliper Perkin Elmer, USA).

### GBM targeting of Nano-reshaper

The biodistribution of Nano-reshaper were further evaluated on mice bearing intracranial GL261 GBM. In brief, GL261 cells (2 × 10^5^) suspended in 5 μL PBS were gently injected into the right striatum of male C57BL/6 mice by using a stereotaxic apparatus. On day 10 after the tumor implantation, mice were randomly divided into two groups and *i.v*. injected DiR-labeled Nano-reshaper or pLIGHT@CaCP. The mice were killed 24 h after the injection. The brains and major organs were separated and evaluated by an IVIS imaging system (Caliper Perkin Elmer, USA).

### Distribution of Nano-reshaper in different cells in brain tumors and immune organs

To investigate the distribution of Nano-reshaper in specific cells in brain tumors and key immune organs, DiR-labeled Nano-reshaper were *i.v.* injected into mice bearing intracranial GL261-GFP GBM. Mice were euthanized at 8 h after the injection and brain tumors, spleens, thymuses, and DLNs were collected. The tissues were filtrated through 70-μm single cell strainers. For spleen samples, the obtained cells were incubated with red blood cell lysis buffer to remove red blood cells, then the cells were washed with PBS and stained with the addition of fluorescently labeled antibodies (Supplementary Table 2). After staining, the cells were washed with PBS, detected via flow cytometry (Beckman, coulter) and analyzed by FlowJo software (X10, Tree star, USA). In addition, we used YOYO-1 to label plasmids encapsulated inside Nano-reshaper and used DiD to label the carrier. The colocalization of DiD and YOYO-1 signals was determined by Image J software.

### In vitro BMDC activation and antigen cross-presentation

The obtained BMDCs (2 × 10^5^) were seeded in 24-well plates, then treated with ApoE-CaCP, ApoE-pLIGHT@CaP or Nano-reshaper (an equivalent CP amount of 4.0 μg/mL and pLIGHT amount of 0.58 μg/mL) for 24 h. Then BMDCs were collected, washed with PBS, and incubated with fluorescently labeled antibodies against CD11c, CD80, and CD86 for 45 min at 4 °C. Then BMDCs were washed with PBS and analyzed by flow cytometry (Beckman, coulter).

To investigate the effect of various formulations on antigen cross-presentation, BMDCs (2 × 10^5^) were pretreated with ApoE-CaCP, ApoE-pLIGHT@CaP or Nano-reshaper (an equivalent CP amount of 4.0 μg/mL and pLIGHT amount of 0.58 μg/mL) for 24 h and pulsed with 100 μg/mL OVA for another 12 h. Then the cells were harvested, washed with PBS and incubated with anti-mouse SIINFEKL/H-2K^b^ monoclonal antibody 25-D1.16 and CD11c antibody for 45 min at 4 °C. Finally, the cells were washed with PBS and analyzed by flow cytometry (Beckman, coulter).

### In vitro macrophage polarization assay

GL261 cells (5 × 10^5^) were cultured in 6-well plates for 24 h, then the medium was centrifuged at 500 g for 10 min and the supernatant was obtained as TCM. To investigate the macrophage polarization in vitro, RAW264.7 cells were planted in 6-well plates, stimulated with the obtained TCM for 24 h followed by adding 5% Glu (control group), ApoE-CaCP, ApoE-pLIGHT@CaP or Nano-reshaper (an equivalent CP amount of 4.0 μg/mL and pLIGHT amount of 0.58 μg/mL) and incubated for 24 h. Then, the cells were collected and washed with PBS. Anti-mouse CD206 and CD80 antibody were added and incubated for 45 min at 4 °C, respectively. Then the cells were washed with PBS and detected by flow cytometry (Beckman, coulter).

### Immune organ index

The tumor-bearing mice were weighed, the spleens and thymus were collected and weighed one day after the last administration. The organ indexes were calculated using the following formula:

Organ index (mg/g) = Weight of organ∕Body weight

### Immunohistochemistry (IF) chemistry analysis

IF staining was performed on frozen sections of brain tumors. Brains were collected and fixed with 4% paraformaldehyde for 48 h at 4 °C, then the brains were gradient dehydrating with 15 and 30% sucrose solution, and were finally performed on tissue optimum cutting temperature (OCT)-freeze medium (Sakura, Torrance, CA, USA) slices. The frozen sections were permeabilized and blocking in Immunol Staining Blocking Buffer (Beyotime Biotechnology Co., Ltd, Nantong, China) at room temperature for 1 h. Then the sections were incubated with fluorescently labeled antibodies or primary antibodies followed by secondary Alexa Flour 488 or Alexa Flour 647-labeled goat anti-rabbit IgG antibodies (Supplementary Table 2). Finally, the sections were stained with Hoechst 33258 and observed using fluorescence microscope (Leica DMI 4000B, Germany). The antibodies used for immunofluorescence staining are listed in Supplementary Table 2. The analysis of positive signals in images were performed by Image J software. Histology staining of main organs and brain tumors were performed on paraffin-embedded sections. All paraffin-embedded tissues were collected, rinsed in PBS and fixed with 4% paraformaldehyde for 48 h. Then tissues were washed with PBS and placed in 70% ethanol solution until paraffin embedded. Then the sections were observed on a microscope (Nikon Corp, Japan) to obtain histological images.

### Flow cytometry assay

Flow cytometry analysis was used to evaluate immune cells in blood, tumors, spleen, thymus and DLNs. In brief, peripheral blood, brain tumors, spleens, thymuses and DLNs were collected from mice one day after the last administration. The tissues were filtrated through 70-μm single cell strainers. For blood and spleen samples, the obtained cells were incubated with red blood cell lysis buffer to remove blood cells, then the cells were washed with PBS and stained with the addition of fluorescently labeled antibodies (Supplementary Table 2). After staining, the cells were washed with PBS, fixed with 4% paraformaldehyde, detected via flow cytometry (Beckman, coulter) and analyzed by FlowJo software (X10, Tree star, USA). Fluorescence conjugated antibodies used in these studies are listed in Supplementary Table 2.

### Establishment of orthotopic GBM models

The orthotopic GBM models using GL261 and G422 were established as previously reported with some modification^16,71^. In brief, 2 × 10^5^ GL261, GL261-luc, GL261-GFP, or G422 cells in 5 μL PBS were injected into right corpus striatum of C57BL/6 or Kunming mice by using a stereotaxic apparatus.

### Therapeutic study in orthotopic GBM models

Mice bearing intracranial GL261, GL261-GFP, GL261-luc, or G422 GBM were randomized blindly into different treatment groups. To investigate the optimum dose of CP in vivo, the treatment regimen was set as follows. Group 1: 5% Glu (*i.v.* injection, 5% Glu = 10 mL/kg), Group 2: ApoE-CaCP (*i.v.* injection, CP = 5 mg/kg), Group 3: ApoE-CaCP (*i.v.* injection, CP = 10 mg/kg), and Group 4: ApoE-CaCP (*i.v.* injection, CP = 20 mg/kg). To explore the therapeutic efficacy of Nano-reshaper on orthotopic GBM, same protocol was applied across the rest experiment groups. 5% Glu (*i.v.* injection, 5% Glu = 10 mL/kg), ApoE-CaCP (*i.v.* injection, CP = 10 mg/kg), ApoE-pLIGHT@CaP (*i.v.* injection, pLIGHT = 1.5 mg/kg), Nano-reshaper (*i.v.* injection, CP = 10 mg/kg; pLIGHT = 1.5 mg/kg), TMZ (*i.p*. injection, 20 mg/kg), αPD-1 (*i.p.* injection, 5 mg/kg), IgG (*i.p*. injection, 5 mg/kg), αCD8 (*i.p.* injection, 10 mg/kg), αCD4 (*i.p.* injection, 10 mg/kg), or FTY720 (*i.p.* injection, 1.5 mg/kg) were given at respective schedules. In vivo orthotopic GBM growth was monitored with the help of IVIS system (Caliper Perkin Elmer, USA) by intraperitoneal injection of luciferin substrate (150 mg/kg, D-Luciferin Potassium Salt, PerkinElmer, USA). For in vivo survival analysis, orthotopic GL261-bearing mice or G422-bearing mice were randomized blindly into different treatment groups and the treatment regimen was the same as mentioned above. During the in vivo experiment mice were observed daily and euthanized when they displayed signs of neurological deficits or 20% weight loss. In some cases, this limit has been exceeded the last day of measurement and the mice were immediately euthanized. The maximum diameter of the mouse tumor volume did not exceed 15 mm, which was approved by the animal care committee of Fudan University School of Pharmacy.

### Tumor rechallege study

Mice survived from in vivo survival analysis were rechallenged with GL261 cells to evaluate immunological memory response. In brief, 2 × 10^5^ GL261 cells in 5 μL PBS were injected into left corpus striatum of five long-term survivors obtained from survival analysis study and five naïve C57BL/6 mice by using a stereotaxic apparatus. Peripheral blood samples were obtained from the mice on day 16 after the incubation of tumor cells and processed to analyze CD3^+^CD8^+^CD44^+^CD62L^-^ effector memory T cell (T_EM_) and CD3^+^CD8^+^CD44^+^CD62L^+^ central memory T cell (T_CM_) by flow cytometry. Mice were observed daily and euthanized when they displayed signs of neurological deficits or 20% weight loss.

### ELISA assay

To determine the chemoattractants responding for the lymphocyte infiltration and immunosuppressive cytokines in tumor microenvironment, ELISA assay was performed to determine CCL21, CXCL13, TGF-β and IL-10 in tumors. Brain tumors were harvested 24 h after the last administration. All specimens were processed and determined according to the manufacturer’s protocols.

### Biosafety evaluation

To evaluate the toxicity of various formulations during the treatment, 15 healthy male C57BL/6 mice (6~10 weeks old, 18~22 g) were randomly divided into five groups and treated with 5% Glu, TMZ, αPD-1, Nano-reshaper, or Nano-reshaper + αPD-1 as described above. After the last administration, the blood and serum were collected and subjected to blood biochemistry and hematology analysis, then the mice were killed and the major organs including heart, liver, spleen, lung, kidney, thymus, and brain were harvested, fixed in 4% paraformaldehyde, embedded in paraffin, sectioned and served for hematoxylin and eosin staining (H&E) for histological analysis.

### Statistical analysis

All statistical analysis was performed by GraphPad Prism 8.0 software. All data were shown as mean ± standard deviation (SD). Comparisons for multiple-group was performed by one-way ANOVA with Tukey tests and two-group comparisons was performed by unpaired Student’s *t* test.

### Reporting summary

Further information on research design is available in the Nature Portfolio Reporting Summary linked to this article.

## Supplementary information

Supplementary Information

Reporting Summary

## Data Availability

The Source Data underlying Figs. 2d, f, g, 3b, d, f, h, 4b–n, 5a–c, e–i, 6b, d, f, h–k, 7c–e, h, i, k, l, 8c–e, i, Supplementary Figs. 5a, b, 10a, b, 11a–c, 13, 12a, b, 15d, 16b, 17b–f, 18b, c, 19c, e, f, h–n, 20a–d, 21c, f–i, 23a–c, 24a–e, 24f, g, 25a–c, 26b, 30b, 31b, 32, 33b, c, 34a, b, e, f, 35a, b, Supplementary Table 1 are provided as a Source Data file. The remaining data are available within the Article, Supplementary Information or Source Data file. Source data are provided with this paper.

## Source data

Source Data
