# Supplementary material for: Reprogramming systemic and local immune function to empower immunotherapy against glioblastoma
Source: Nat Commun. 2023 Jan 26;14:435. doi: 10.1038/s41467-023-35957-8 (PMC9880004; doi:10.1038/s41467-023-35957-8)
Supplement: Supplementary file 1 — Supplementary Information [file 41467_2023_35957_MOESM1_ESM.pdf]

Reprogramming systemic and local immune function to empower immunotherapy against glioblastoma

Songlei Zhou[1,2], Yukun Huang[1,2,3], Yu Chen[1,2], Yipu Liu[1,2], Laozhi Xie[1,2], Yang You[1,2], Shiqiang Tong[1,2], Jianpei Xu[1,2], Gan Jiang[3], Qingxiang Song[3], Ni Mei[4], Fenfen Ma[1,2,5], Xiaoling Gao[3*], Hongzhuan Chen[6*], Jun Chen[1,2*]

[1]Department of Pharmaceutics, School of Pharmacy & Shanghai Pudong Hospital, Fudan University; Shanghai, 201203, China.

[2]Key Laboratory of Smart Drug Delivery, Ministry of Education, School of Pharmacy, Fudan University; Shanghai, 201203, China.

[3]Department of Pharmacology and Chemical Biology, State Key Laboratory of Oncogenes and Related Genes, Shanghai Universities Collaborative Innovation Center for Translational Medicine, Shanghai Jiao Tong University School of Medicine; Shanghai, 200025, China.

[4]Shanghai Center for Drug Evaluation and Inspection; Shanghai, 201210, China.

[5]Department of Pharmacy, Shanghai Pudong Hospital, Fudan University; Shanghai, 201399, China.

[6]Institute of Interdisciplinary Integrative Medicine Research, Shuguang Hospital, Shanghai University of Traditional Chinese Medicine, Shanghai, 201203, China.

*Corresponding author. Email: chenjun@fudan.edu.cn (J.C.); yaoli@shsmu.edu.cn (H.-Z.C); shellygao1@sjtu.edu.cn (X.-L.G.)

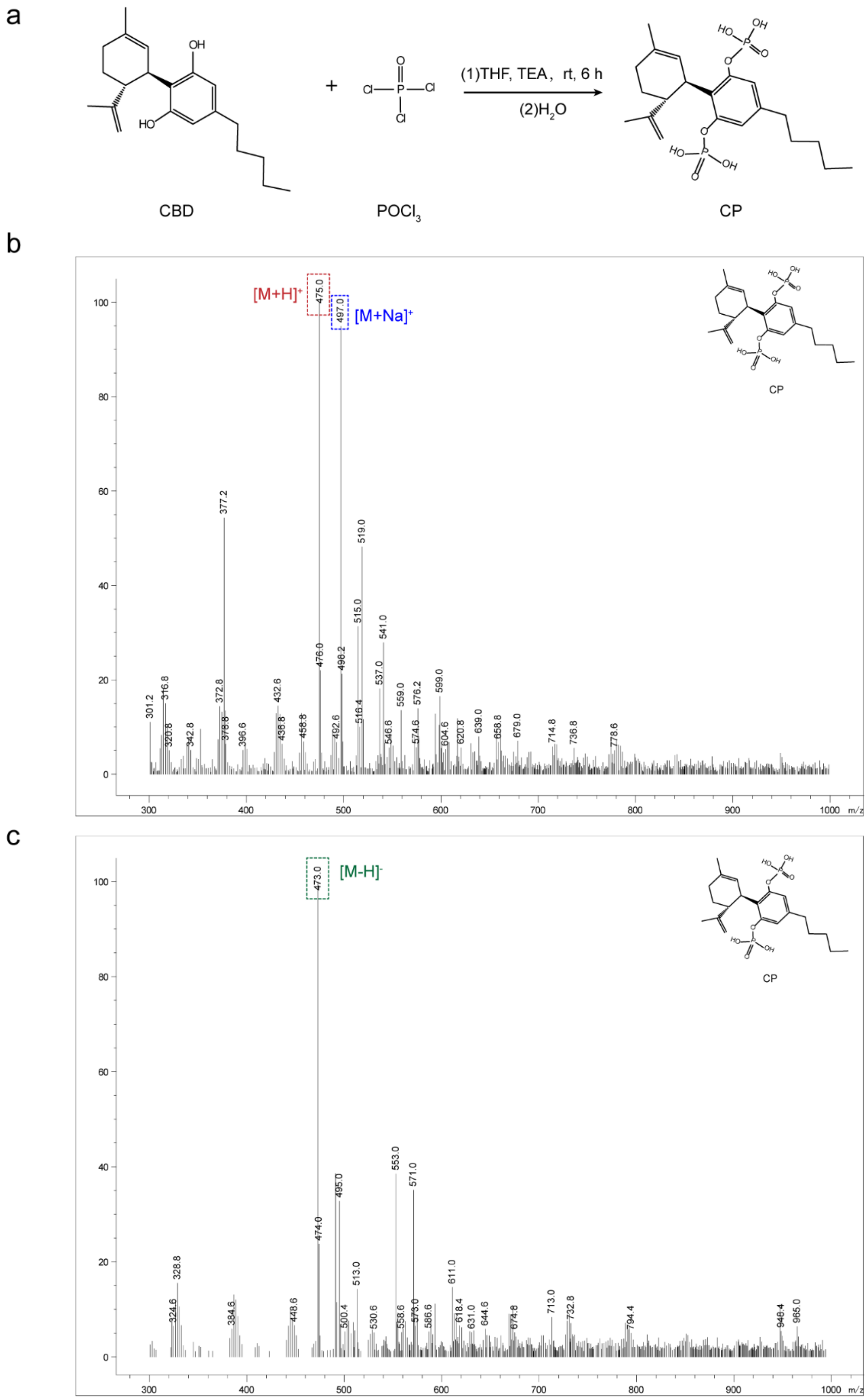

Supplementary Figure 1 The synthesis and characterization of CP. (a) The synthetic route of CP. The mass spectrum of CP for positive-ion mode (b) and negative-ion mode (c). CP, cannabidiol phosphate.

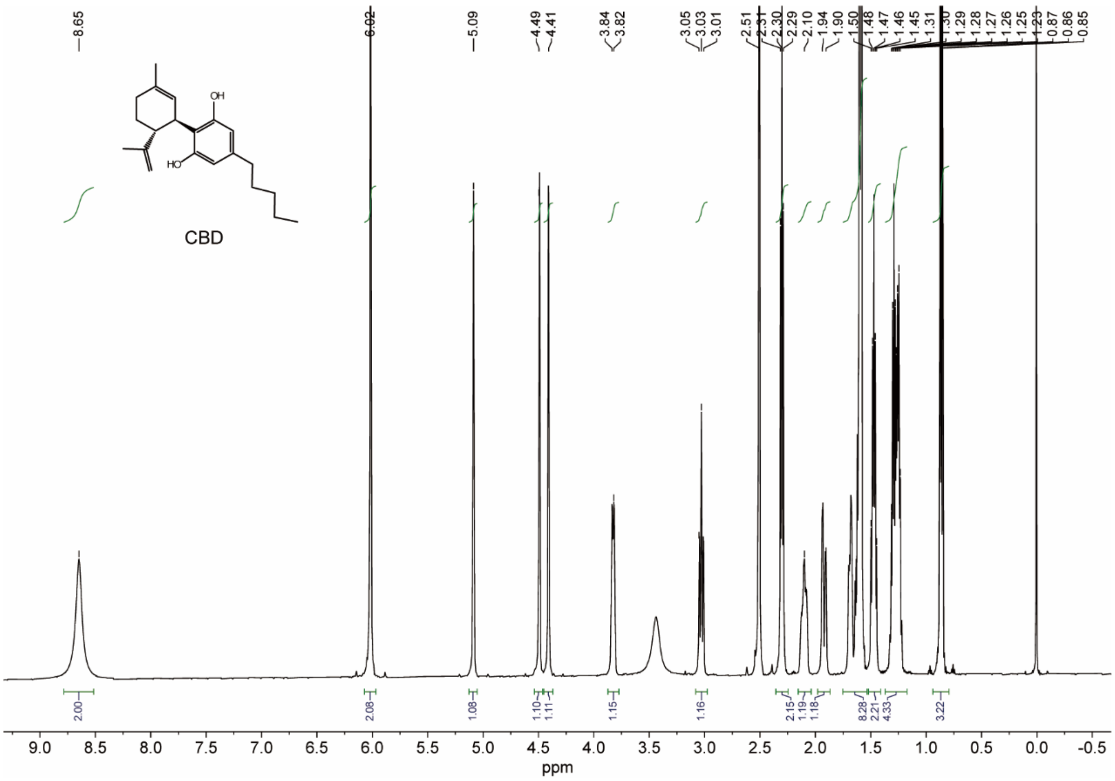

Supplementary Figure 2 The ^{1}H-NMR spectrum (600 MHz, DMSO-d_6) of CBD: δ 8.65 (s, 2H), 6.02 (s, 2H), 5.09 (s, 1H), 4.49 (s, 1H), 4.41 (s, 1H), 3.83 (d, J = 10.6 Hz, 1H), 3.03 (t, J = 12.9 Hz, 1H), 2.30 (t, J = 7.7 Hz, 2H), 2.10 (s, 1H), 1.92 (d, J = 20.8 Hz, 1H), 1.75 - 1.54 (m, 8H), 1.47 (p, J = 7.5 Hz, 2H), 1.27 (dq, J = 27.8, 8.1, 7.3 Hz, 4H), 0.86 (t, J = 7.1 Hz, 3H).

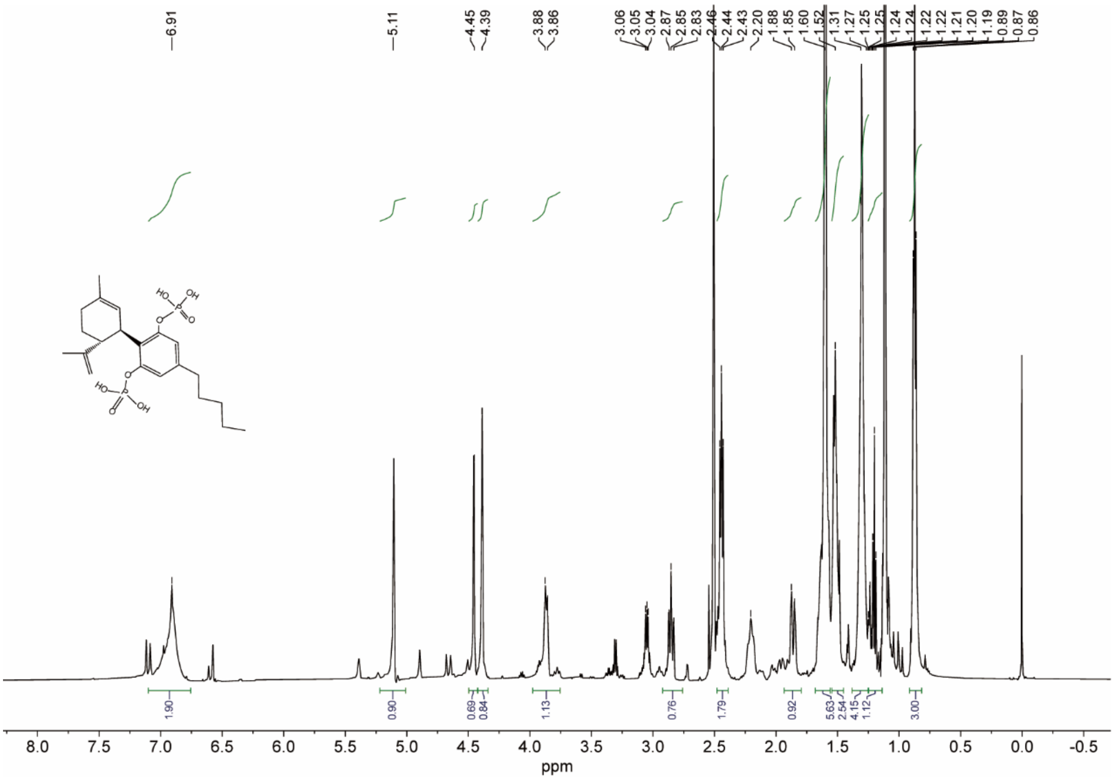

Supplementary Figure 3 The ^{1}H-NMR spectrum (600 MHz, DMSO-d_6) of CP: δ

6.91 (s, 2H), 5.11 (s, 1H), 4.45 (s, 1H), 4.39 (s, 1H), 3.87 (d, J = 12.1 Hz, 2H), 2.91 - 2.82 (m, 1H), 2.48 - 2.40 (t, 2H), 2.20 (s, 1H), 1.60 (s, 6H), 1.52 (s, 2H), 1.31 (s, 4H), 1.26 - 1.18 (m, 2H), 0.87 (t, J = 6.9 Hz, 3H).

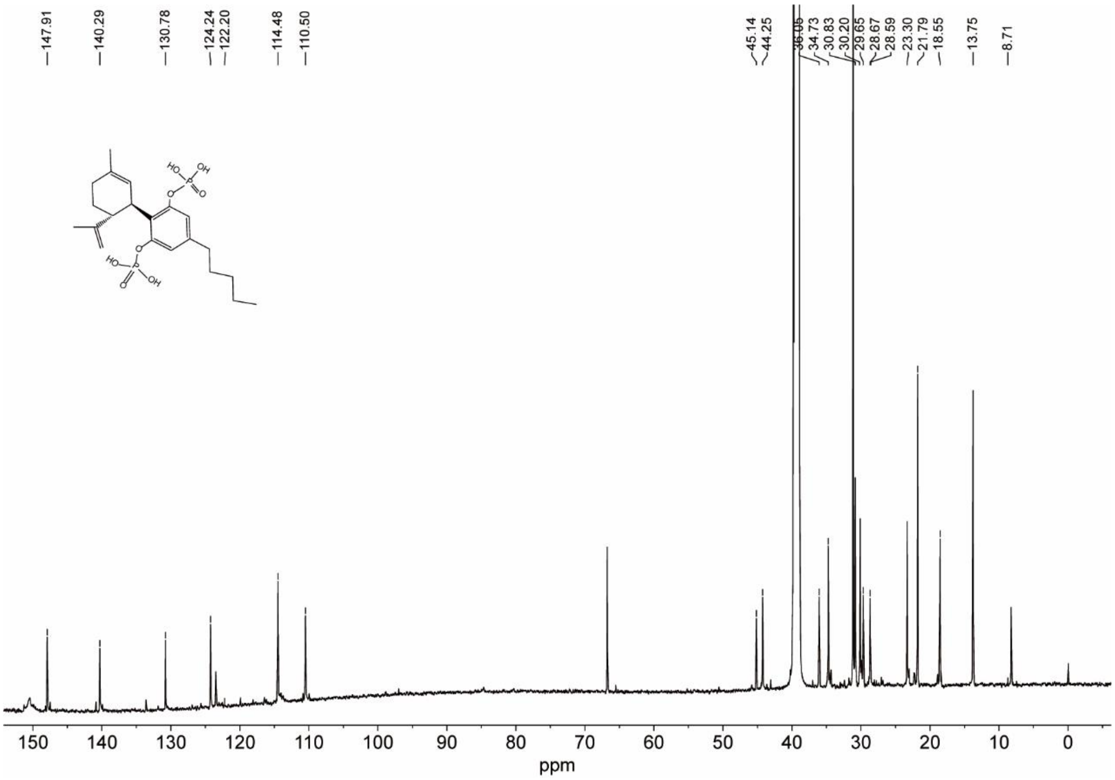

Supplementary Figure 4 ^{13}C-NMR spectra (150 MHz, DMSO-d_6) of CP: δ = 147.91, 140.29, 130.78, 124.24, 122.20, 114.48, 110.50, 45.14, 44.25, 36.05, 34.73, 30.83, 30.20, 29.65, 28.67, 28.59, 23.30, 21.79, 18.55, 13.75, 8.71.

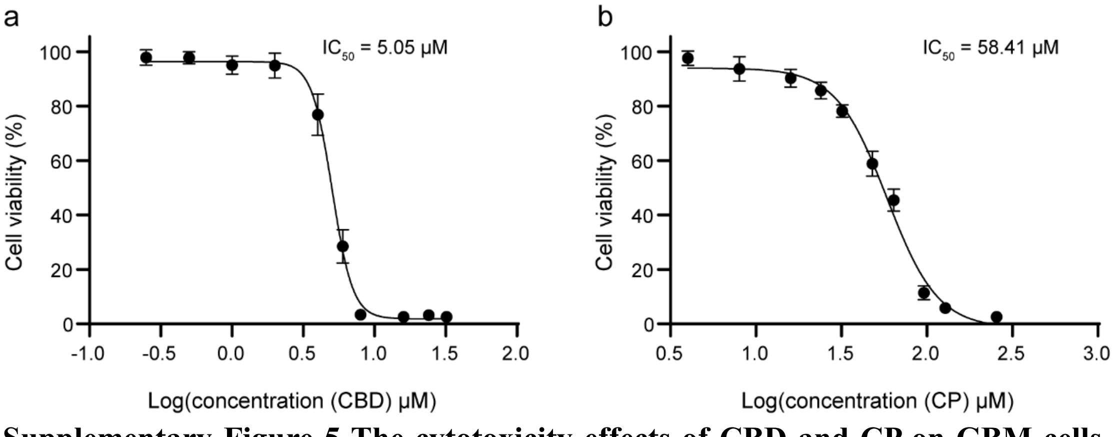

Supplementary Figure 5 The cytotoxicity effects of CBD and CP on GBM cells. MTT assays of GL261 cells following 24 h incubation with (a) CBD or (b) CP (n = 6 samples per group). Data were shown as mean ± SD. Error bars represent SD. Source data are provided as a Source Data file.

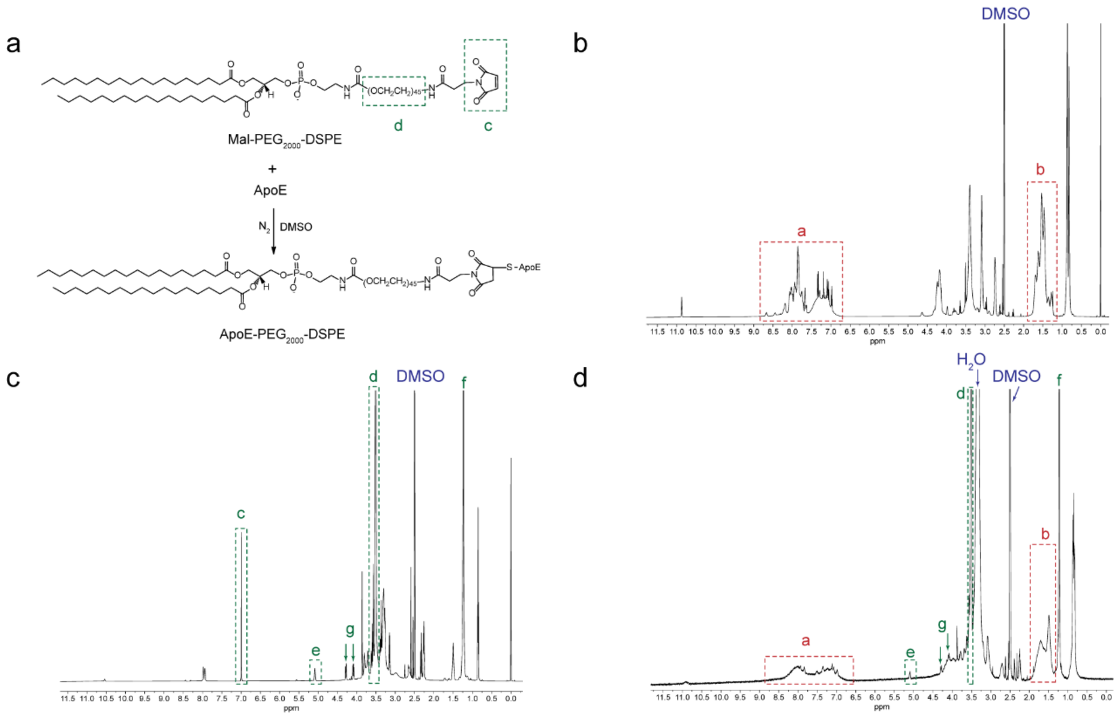

Supplementary Figure 6 Synthesis and characterization of ApoE-PEG$_{2000}$-DSPE. (a) Synthetic route of ApoE-PEG$_{2000}$-DSPE. ^{1}H-NMR spectrum of (b) ApoE, (c) Mal-PEG$_{2000}$-DSPE and (d) ApoE-PEG$_{2000}$-DSPE in DMSO-d_6.

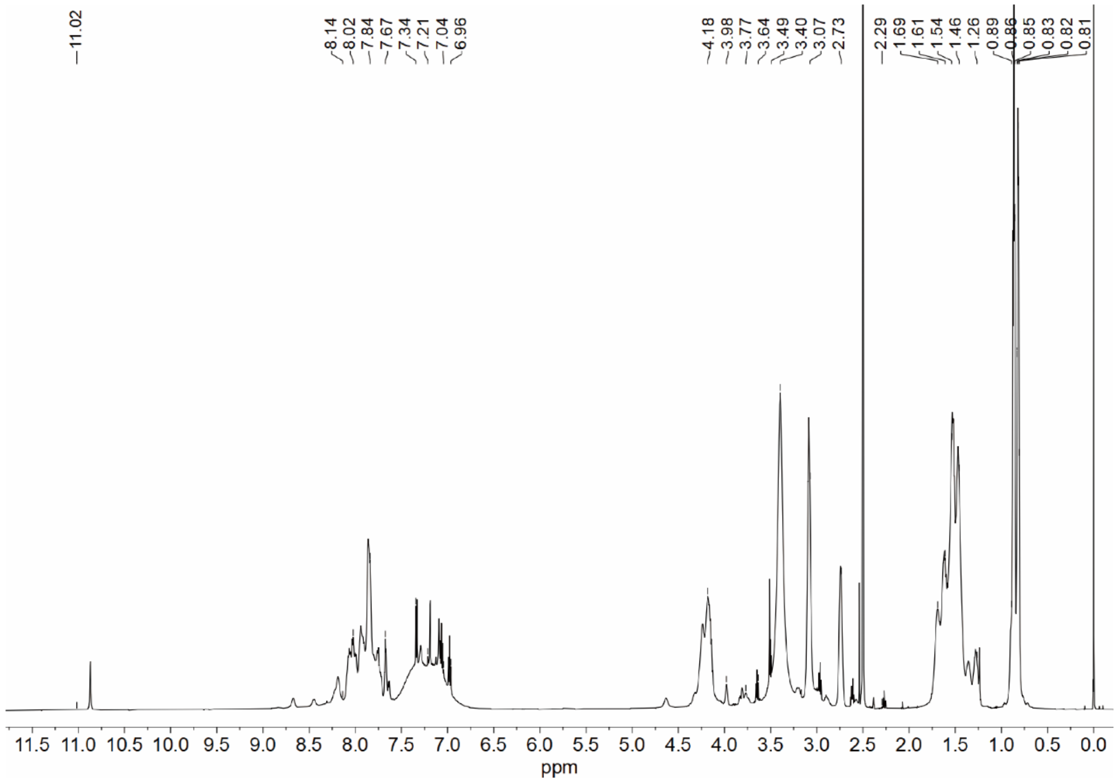

Supplementary Figure 7 The ^{1}H-NMR spectrum (600 MHz, DMSO-d_6) of ApoE.

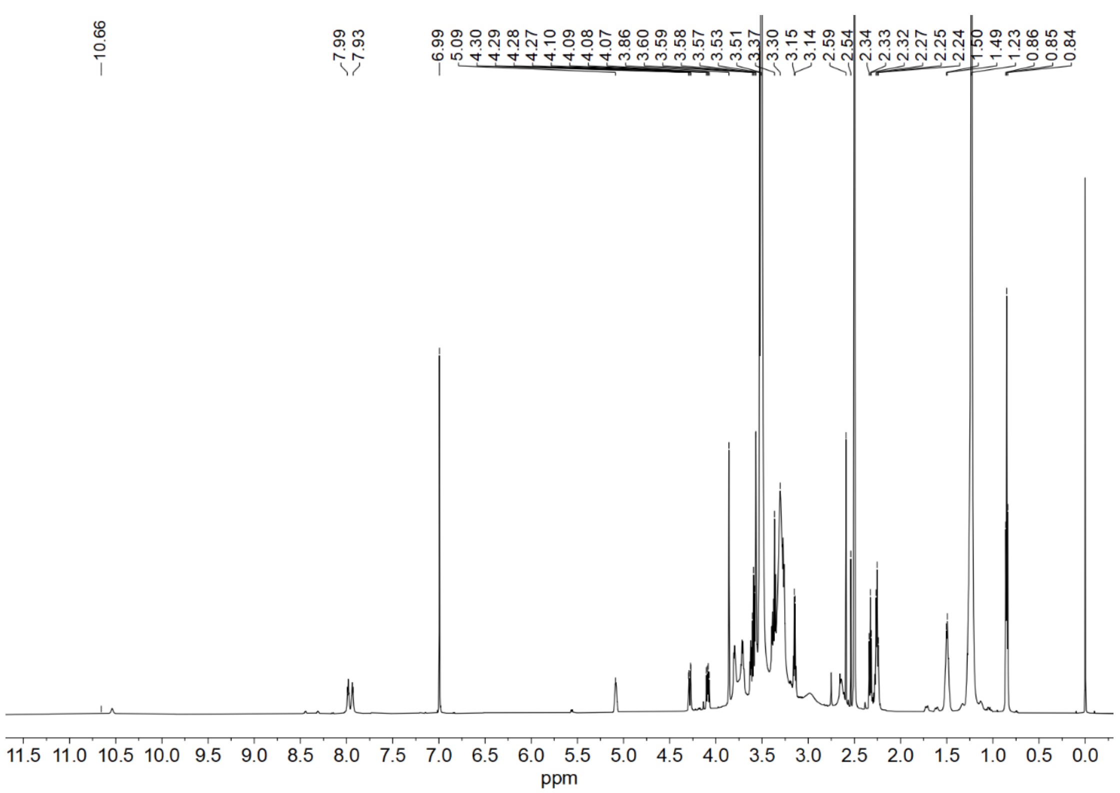

Supplementary Figure 8 The ^{1}H-NMR spectrum (600 MHz, DMSO-*d₆*) of Mal-PEG2000-DSPE.

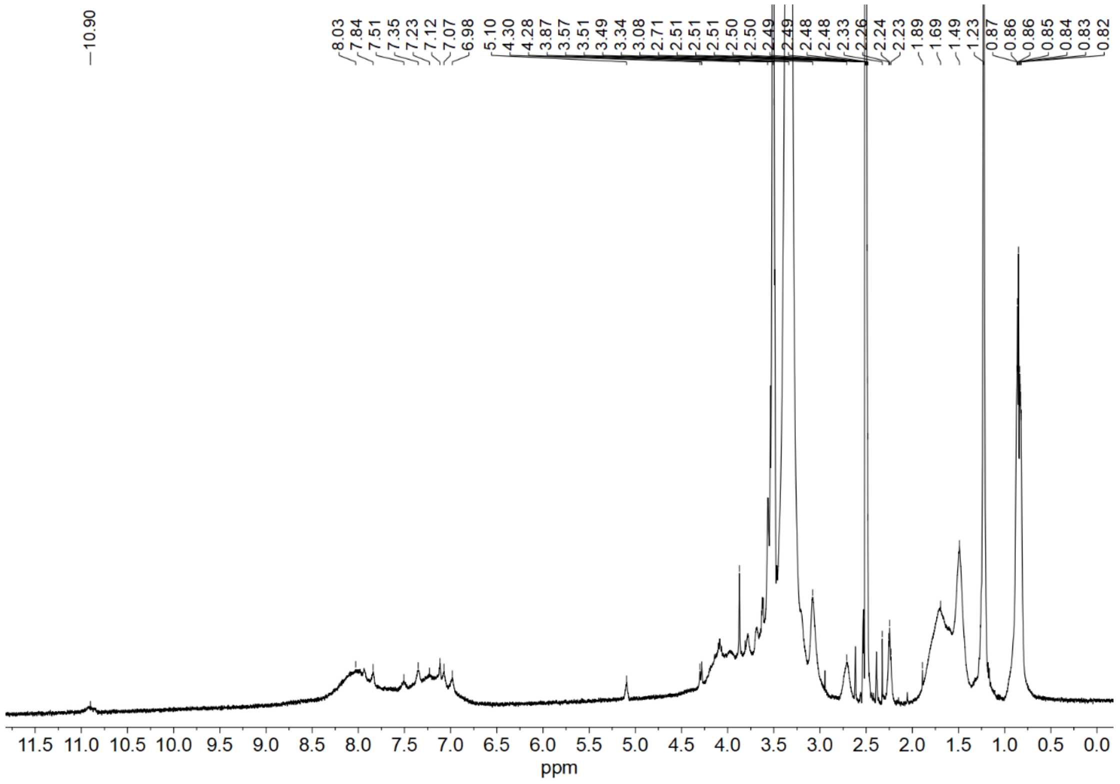

Supplementary Figure 9 The ^{1}H-NMR spectrum (600 MHz, DMSO-d_6) of ApoE-PEG$_{2000}$-DSPE.

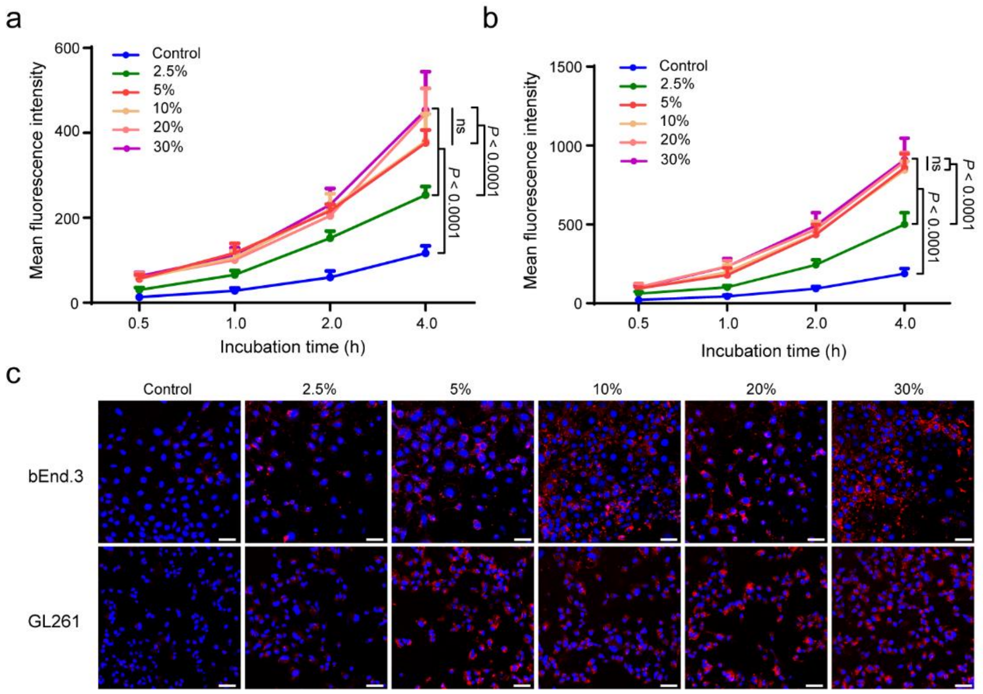

Supplementary Figure 10 Cellular uptake of different ApoE density decorated CaP nanoparticles by bEnd.3 and GL261 cells. DiI labeled CaP nanoparticles were set as the control group and the others were different ApoE density decorated CaP nanoparticles. (a) Quantitative results of cellular uptake of DiI-labeled nanoparticles by bEnd.3 cells for 0.5, 1, 2 and 4 h incubation at 37°C (n = 6 samples per group). (b) Quantitative cellular uptake of DiI-labeled nanoparticles by GL261 cells for 0.5, 1, 2 and 4 h incubation at 37°C (n = 6 samples per group). (c) Representative images of bEnd.3 and GL261 cells incubated with different ApoE density decorated DiI-labeled nanoparticles for 4 h at 37°C. Scale bar, 50 μm. Data were shown as mean ± SD. Error bars represent SD. Significant differences were evaluated in (a) and (b) using two-way ANOVA with multiple comparisons. Ns, not significant. Source data are provided as a Source Data file. The experiments were repeated three times independently.

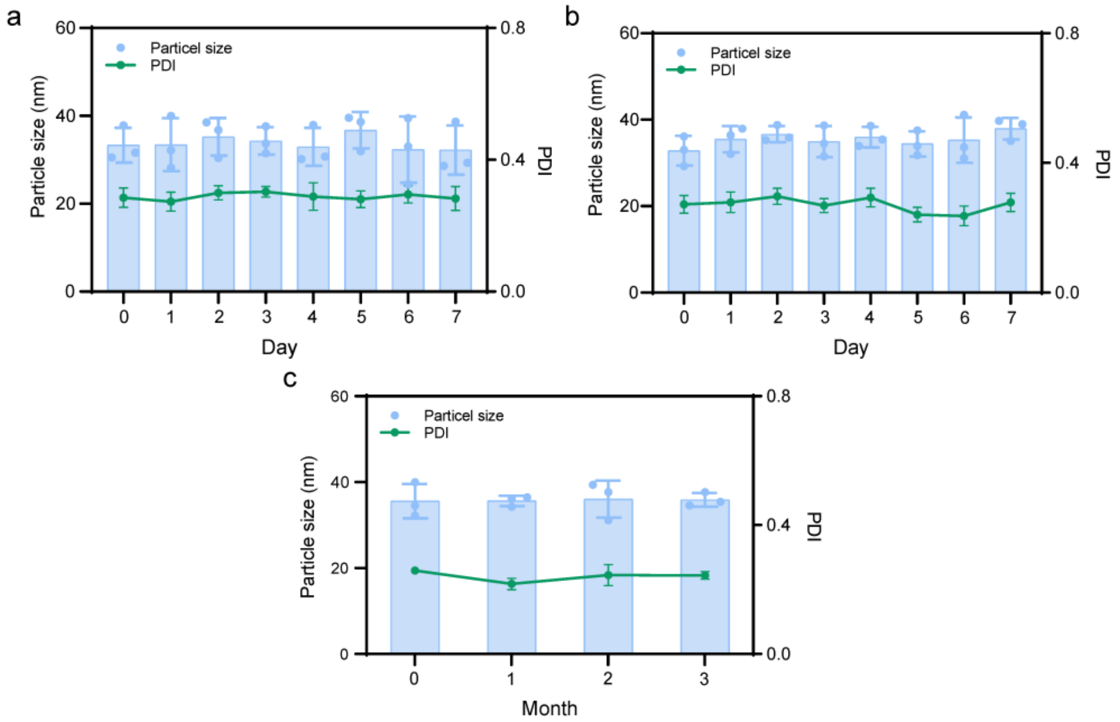

Supplementary Figure 11 *In vitro* stability of Nano-reshaper. Stability of Nano-reshaper after dilution with 5% Glu (a) and 0.9% NaCl (b) (*n* = 3 samples per group). (c) Stability of Nano-reshaper after storage for 3 months (*n* = 3 samples per group). Data were shown as mean ± SD. Error bars represent SD. Source data are provided as a Source Data file.

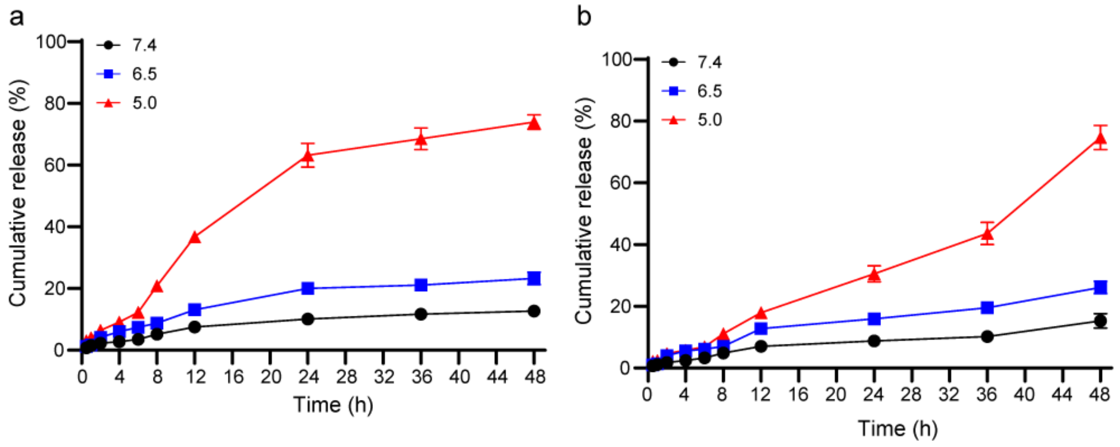

Supplementary Figure 12 *In vitro* release investigation of Nano-reshaper. The release profile of CP (a) and pLGIHT (b) in PBS (pH 5.0, pH 6.5 and pH 7.4) at 37°C (*n* = 3 samples per group). Data were shown as mean ± SD. Error bars represent SD. Source data are provided as a Source Data file.

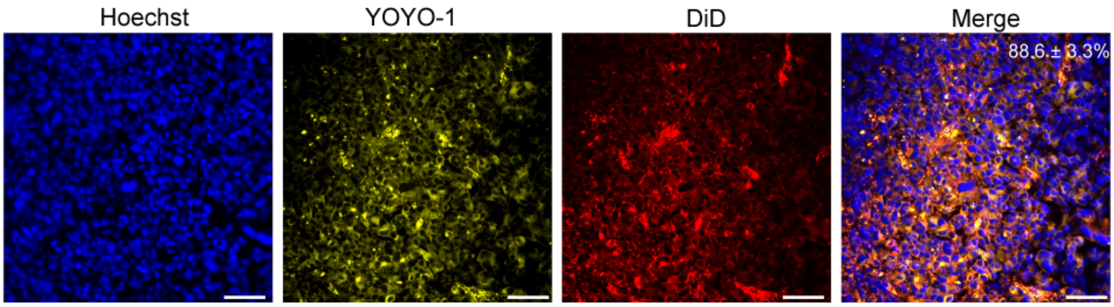

Supplementary Figure 13 *In vivo* stability assessment of Nano-reshaper. Tumor slices obtained from GL261-bearing mice after injection of YOYO-1 and DiD labeled Nano-reshaper (*n* = 5 samples per group). Colocalization coefficient was assessed through Image J software and marked in the top right-hand corner of the figure. Scale bar = 50 μm. Data were shown as mean ± SD. Error bars represent SD. Source data are provided as a Source Data file. The experiments were repeated three times independently.

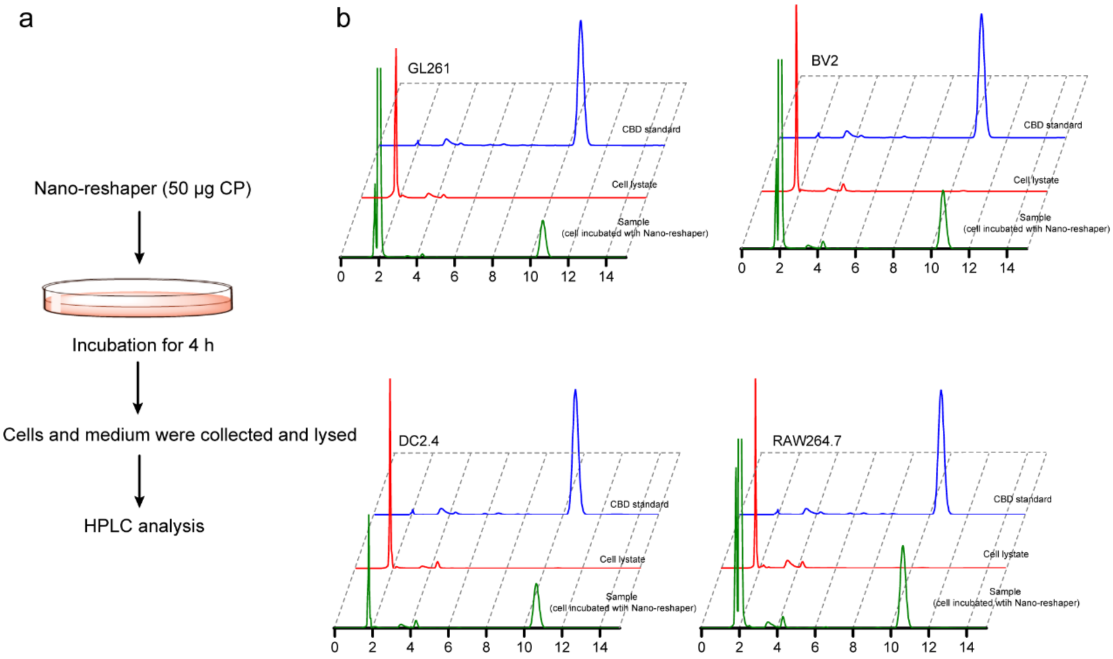

Supplementary Figure 14 The degradation of Nano-reshaper in different cells. (a) Scheme for degradation study. (b) HPLC analysis of Nano-reshaper after incubating with GL261, BV2, DC2.4 and RAW264.7 cells.

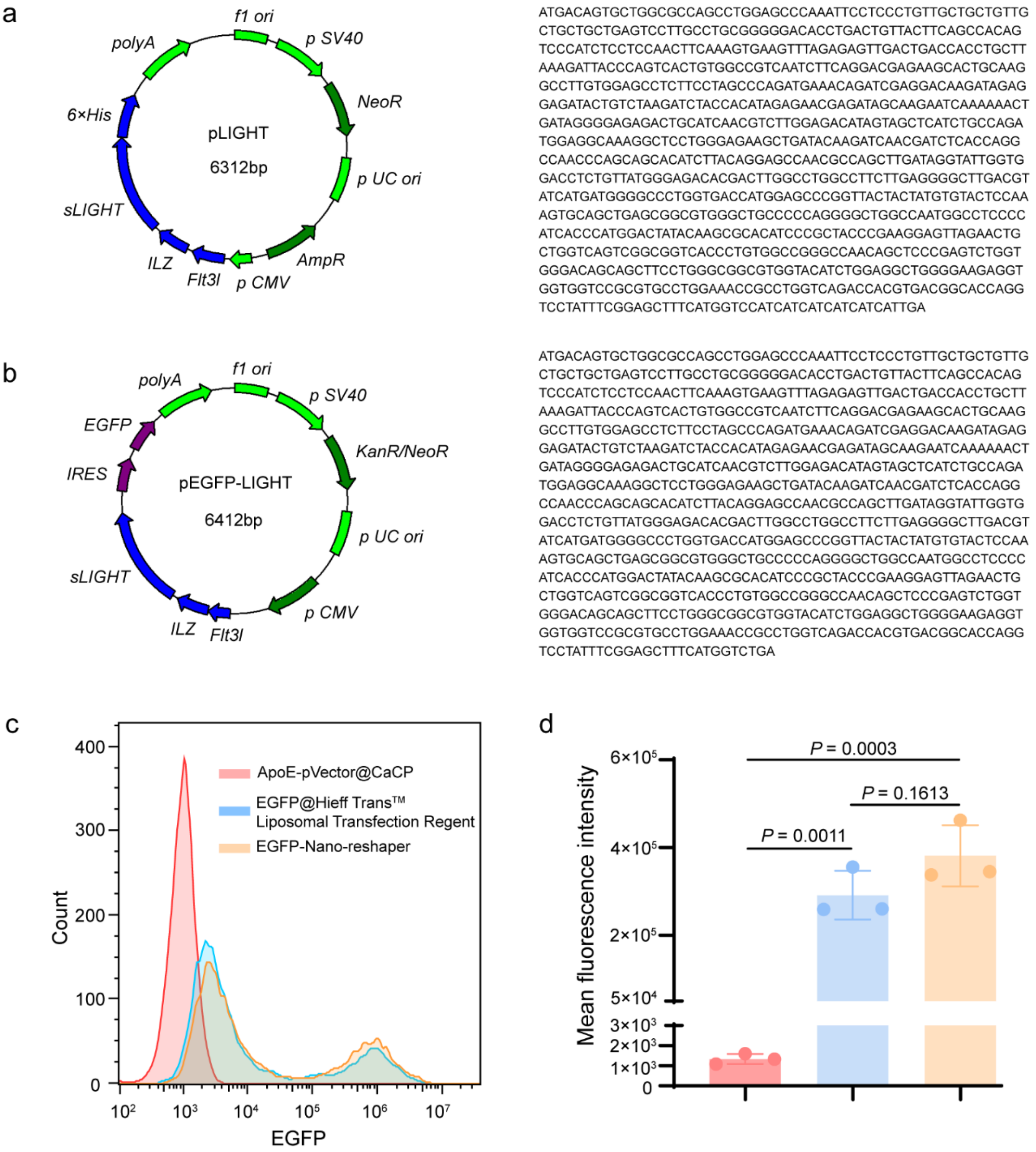

Supplementary Figure 15 Representative schemes of plasmid and EGFP expression with various treatments. (a) Vector map and the sequence of plasmid encoding LIGHT and 6× His. (b) Vector map and the sequence of plasmid simultaneously encoding EGFP and LIGHT. (c) EGFP transfection efficiency was analyzed by flow cytometry. The non-EGFP coding vector plasmids were encapsulated in CaP nanoparticles (ApoE-pVector@CaCP). (d) Quantitative results of EGFP fluorescence intensity after treatment with various formulations (n = 3 samples per group). Data were shown as mean ± SD. Error bars represent SD. Significant difference was evaluated in (d) using one-way ANOVA with Tukey multiple comparisons post-test. Source data are provided as a Source Data file.

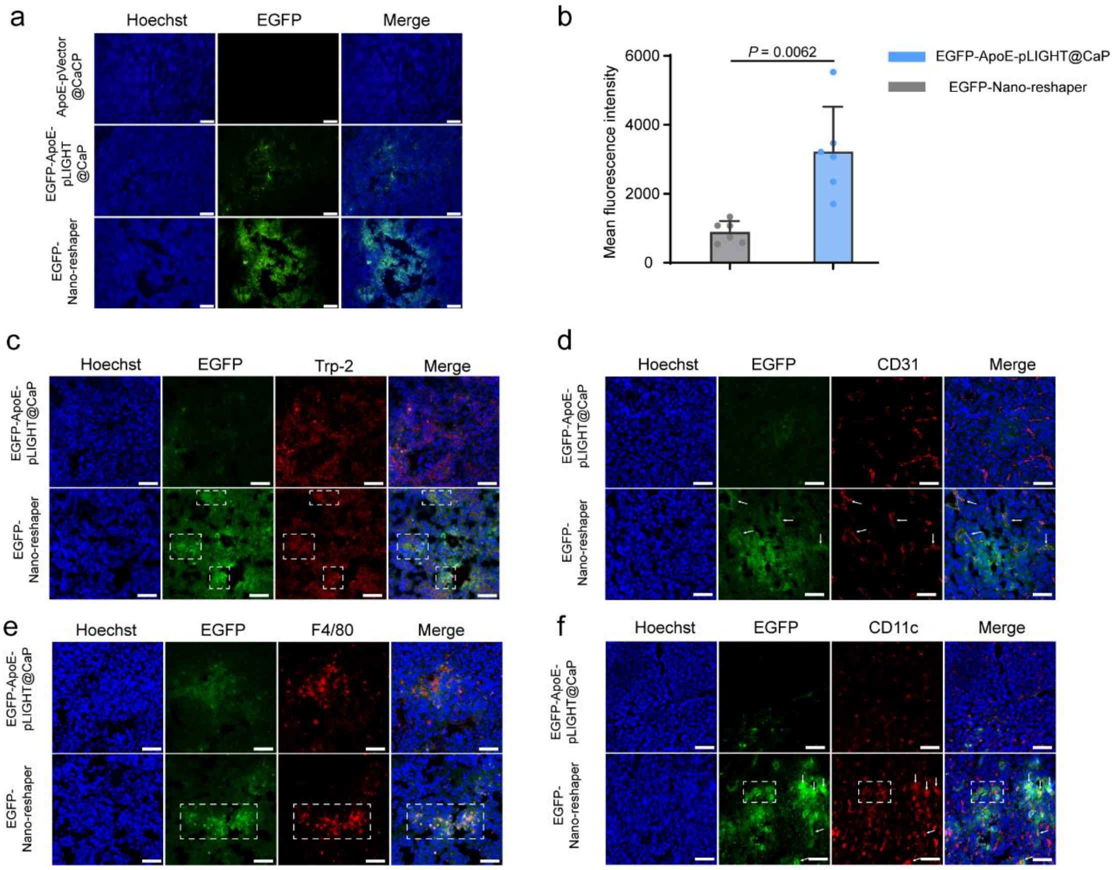

Supplementary Figure 16 EGFP expression in brain tumors. (a) Microscopy analysis of EGFP signals in brain tumors after two injections of ApoE-pVector@CaCP, EGFP-ApoE-pLIGHT@CaP and EGFP-Nano-reshaper. (b) Semi-quantitative results of GFP signals (n = 6 samples per group). Scale bar, 100 µm. EGFP signals were observed in Trp-2 (c), CD31 (d), F4/80 (e) and CD11c (f) positive cells. Scale bar, 100 µm. Data were shown as mean ± SD. Error bars represent SD. Significant difference was evaluated in (b) using two-tailed unpaired Student's t test. Source data are provided as a Source Data file. All the imaging experiments were repeated three times independently.

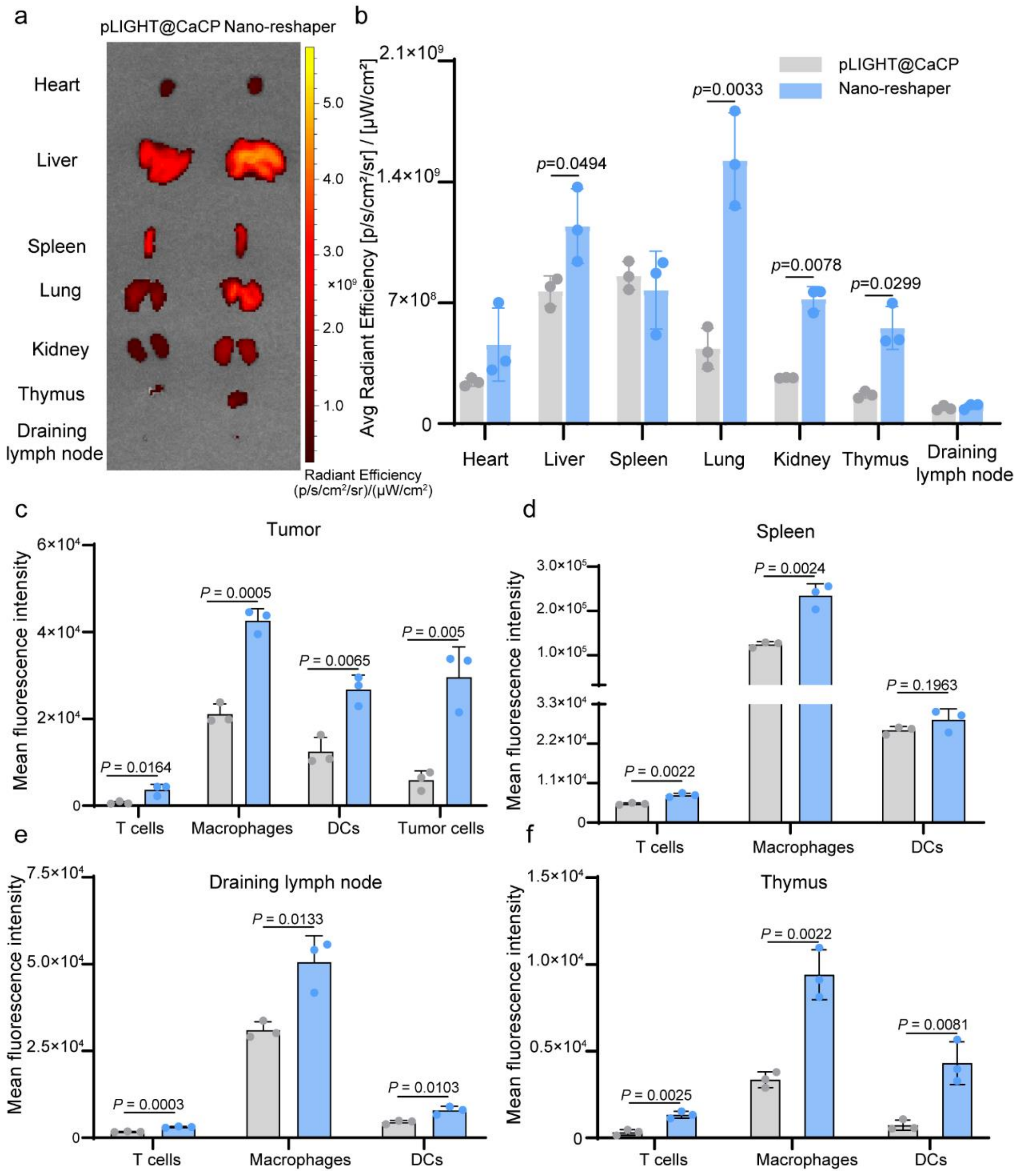

Supplementary Figure 17 The effect of ApoE peptide on the biodistribution of CaP nanoparticles in GL261-bearing mice. (a) Representative DiR fluorescence imaging of major organs obtained from mice bearing intracranial GL261 glioma after *i.v.* injection of DiR-labeled pLIGHT@CaCP and Nano-reshaper. (b) Semi-quantitative analysis based on fluorescence signal intensity (n = 3 mice). Mean DiR fluorescence intensity of T cells, macrophages, DCs and tumor cells in brain tumors (c), spleens (d), draining lymph nodes (e) and thymuses (f) obtained from mice bearing intracranial GL261 glioma after *i.v.* injection of DiR-labeled pLIGHT@CaCP and Nano-reshaper. Data were shown as mean ± SD. Error bars represent SD. Significant difference was evaluated in (b-f) using two-tailed unpaired Student's t test. Source data are provided as a Source Data file.

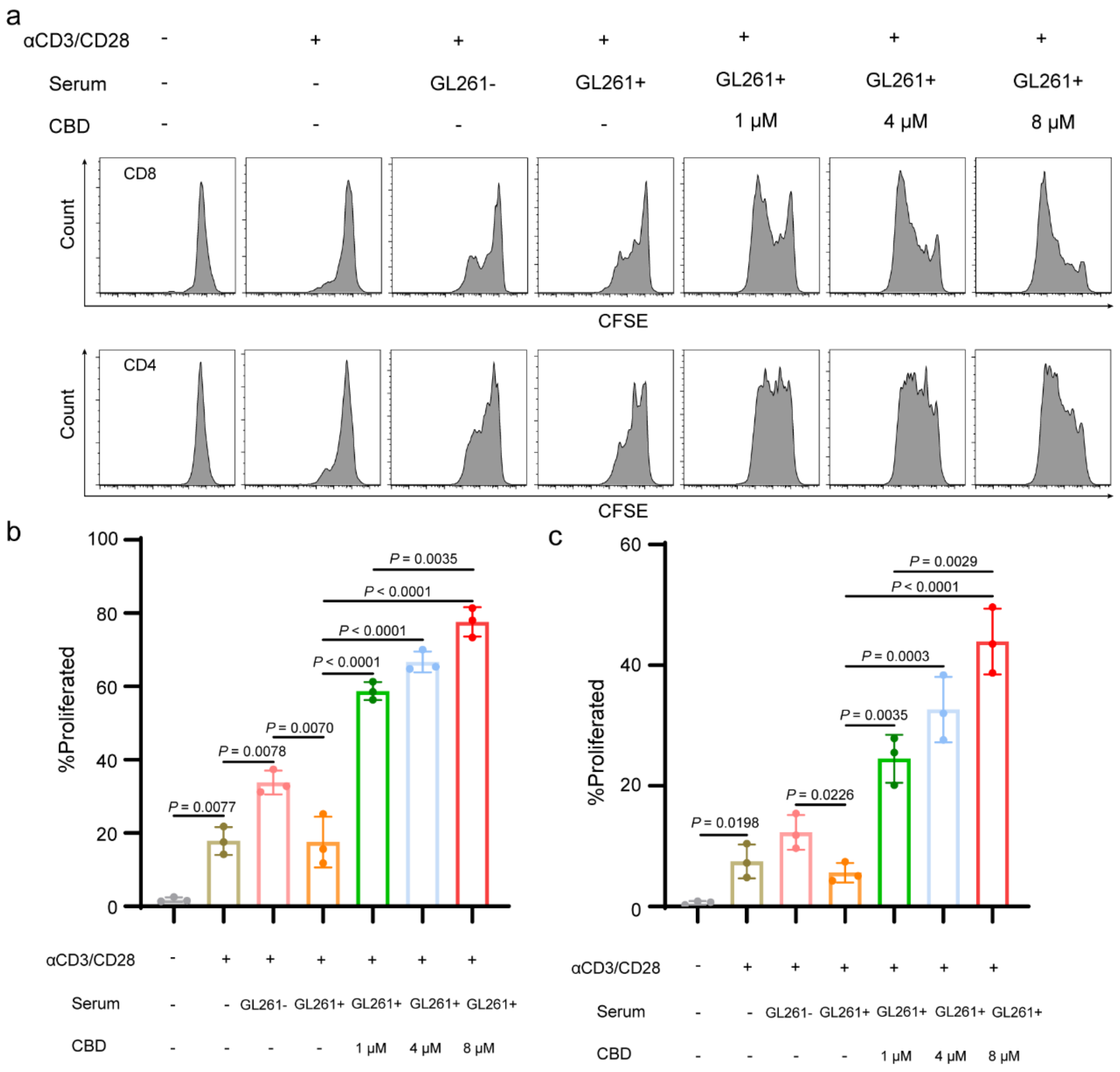

Supplementary Figure 18 CBD promoted T-cell proliferation *in vitro*. (a) Representative flow cytometry histograms depicted CD8^{+} (top) and CD4^{+} (bottom) T cells proliferation in the presence of anti-CD3/CD28 antibody (αCD3/CD28) and serum from tumor-free mice (GL261-) and GL261-bearing mice (GL261+), along with different concentrations of CBD. Sham operation mice were set as "GL261-" group and GL261-bearing mice were set as "GL261+" group. T cells were isolated from spleens of naïve C57BL/6 mice and labeled with CFSE. The percent of CFSE dilution in CD8^{+} (b) and CD4^{+} T cell (c) were quantified as a detection of proliferation under various conditions (n = 3 samples per group). Data were shown as mean ± SD. Error bars represent SD. Significant differences were evaluated in (b) and (c) using one-way ANOVA with Tukey multiple comparisons post-test. Source data are provided as a Source Data file.

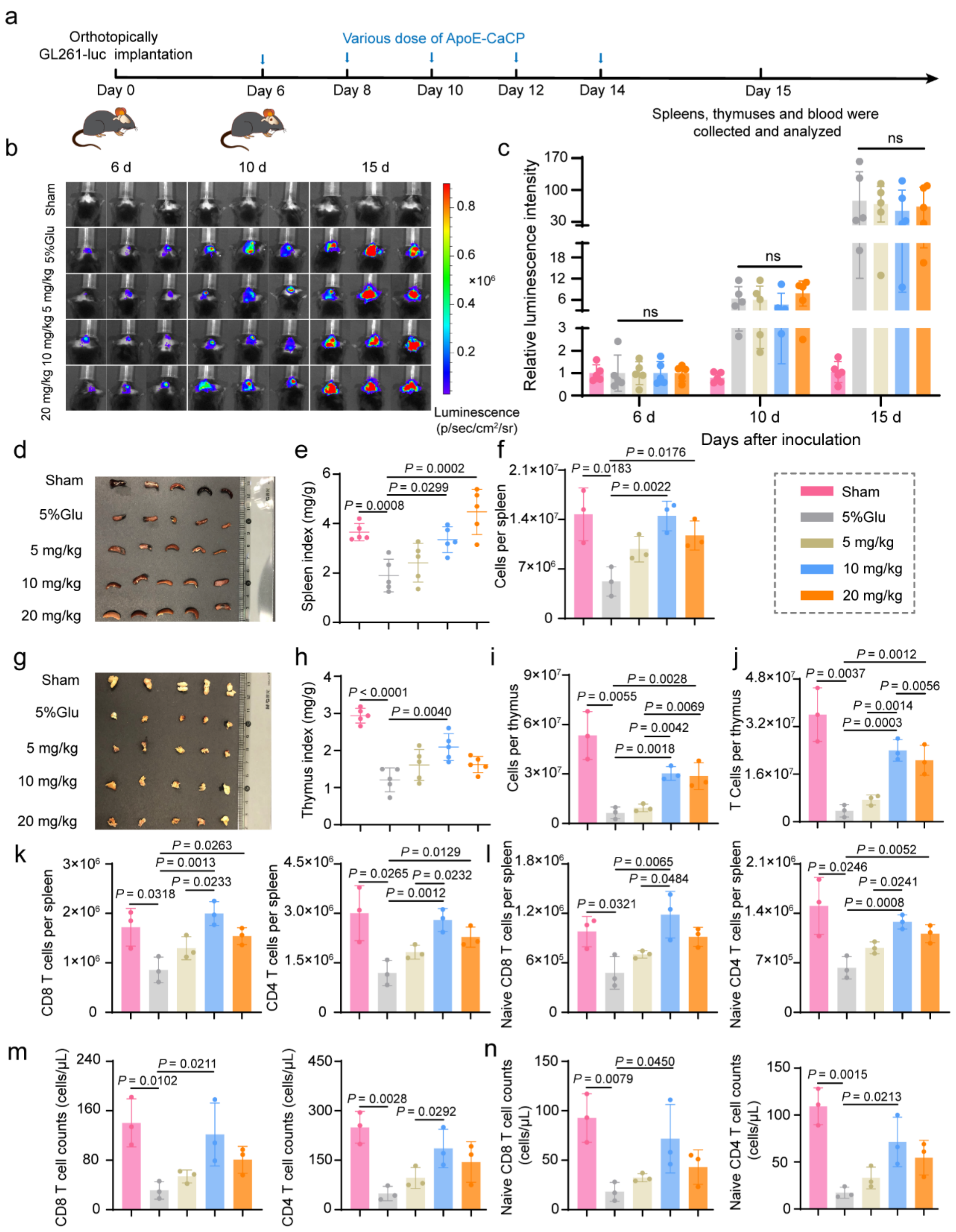

Supplementary Figure 19 ApoE-CaCP exhibited negligible anti-glioma efficacy but enhanced systemic immunity. (a) Orthotopic tumor implantation and treatment scheme for various dose of ApoE-CaCP therapy. (b) Representative *in vivo* bioluminescence imaging of mice bearing intracranial GL261-luc glioma after treatment with various dose of ApoE-CaCP on days 6, 10 and 15 post tumor inoculation. (c) Semi-quantitative results of GL261-luc burden by bioluminescence intensity (n = 5 mice). Images of the dissected spleens (d) and thymuses (g) in various groups on day 15. Spleen index (e) and thymus index (h) of GL261-luc-bearing mice receiving various dose of ApoE-CaCP (n = 5 mice). Cell counts of per spleen (f) and thymus (i). (j) T cell

counts in thymus collected from GL261-luc-bearing mice receiving various dose of ApoE-CaCP (n = 3 samples per group). Total counts of CD8, CD4 (k), naïve CD8 and naïve CD4 (l) T cells in spleens collected from GL261-luc-bearing mice receiving various dose of ApoE-CaCP (n = 3 samples per group). Blood CD8, CD4 (m), naïve CD8 and naïve CD4 (n) T cell counts in GL261-luc-bearing mice receiving various dose of ApoE-CaCP (n = 3 samples per group). Mice receiving PBS injection instead of GL261-luc cells in brains were set as the sham group. Data were shown as mean ± SD. Error bars represent SD. Significant differences were evaluated in (c), (e-f) and (h-n) using one-way ANOVA with Tukey multiple comparisons post-test. Ns, not significant. Source data are provided as a Source Data file.

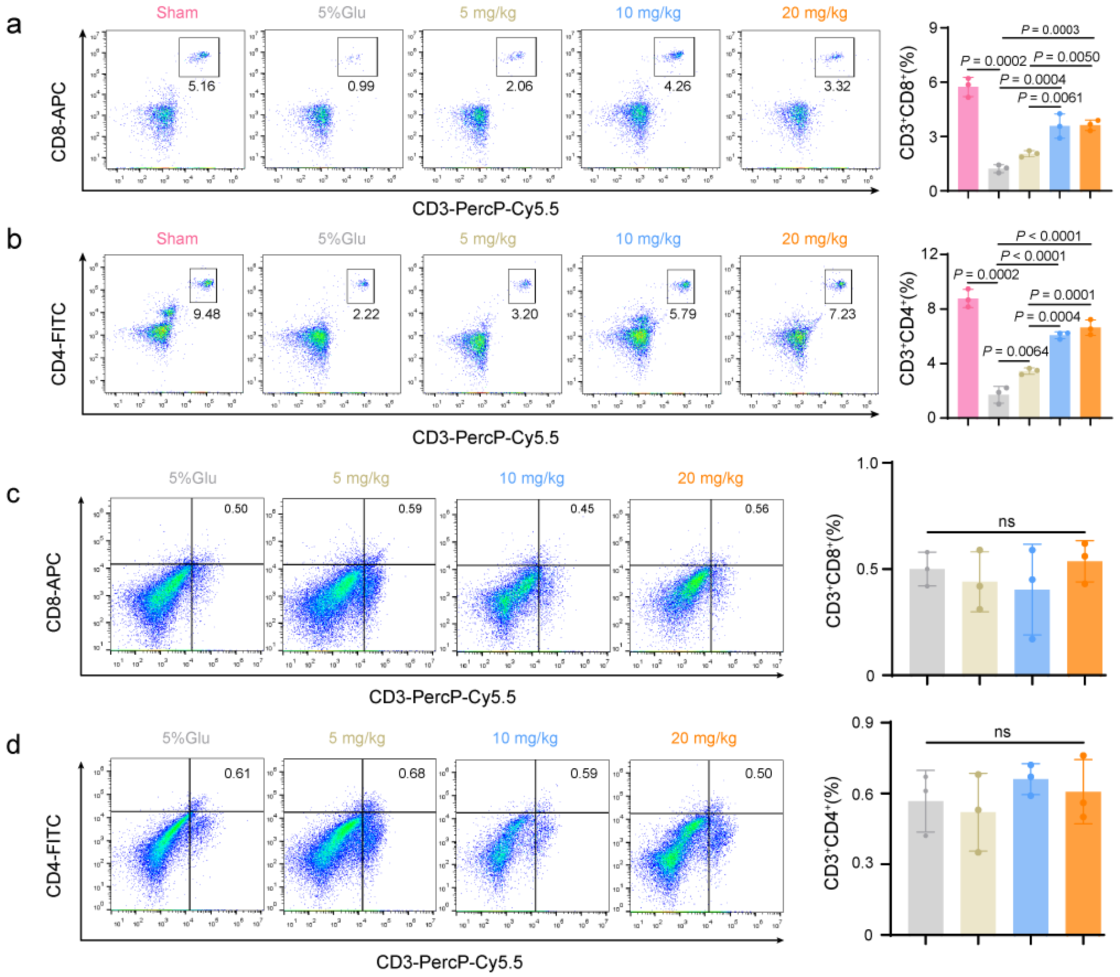

Supplementary Figure 20 ApoE-CaCP increased T cells frequency in peripheral blood but did not significantly changed T cells in brain tumors. Flow cytometry analysis of $CD3^+CD8^+$ (a) and $CD3^+CD4^+$ cells (b) in blood collected from different group mice. Mice receiving PBS injection instead of GL261-luc cells were set as the sham group (n = 3 mice). Flow cytometry analysis of (c) $CD3^+CD8^+$ and (d) $CD3^+CD4^+$ cells in brain tumors (n = 3 mice). Data were shown as mean ± SD. Error bars represent SD. Significant differences were evaluated in (a-d) using one-way ANOVA with Tukey multiple comparisons post-test. Ns, not significant. Source data are provided as a Source Data file.

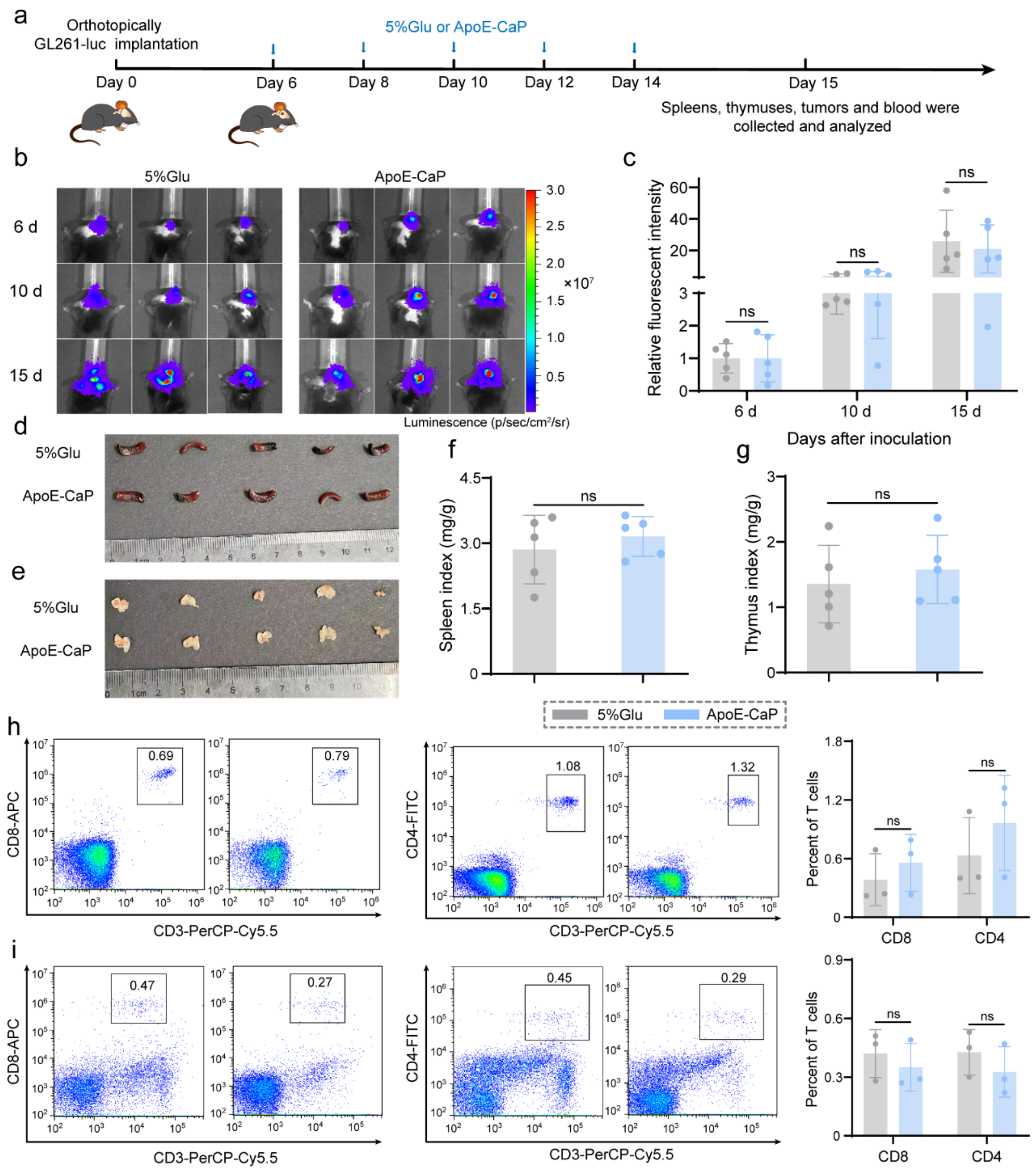

Supplementary Figure 21 ApoE-CaP had no significant antitumor activity and immune regulation effect. (a) Orthotopic tumor implantation and treatment scheme for 5% Glu and ApoE-CaP treatment. (b) Representative *in vivo* bioluminescence imaging of mice bearing intracranial GL261-luc glioma after treatment with 5%Glu and ApoE-CaP on days 6, 10 and 15 post tumor inoculation. (c) Semi-quantitative results of GL261-luc burden by bioluminescence intensity (n = 5 mice). Images of the dissected spleens (d) and thymuses (e) in various groups on day 15. Spleen index (f) and thymus index (g) of GL261-luc-bearing mice after receiving 5%Glu and ApoE-CaP (n = 5 mice). Flow cytometry analysis of $CD3^{+}CD8^{+}$ and $CD3^{+}CD4^{+}$ cells in peripheral blood (h) and brain tumors (i) (n = 3 mice). Data were shown as mean ± SD. Error bars represent SD. Significant differences were evaluated in (c) and (f-i) using two-tailed unpaired Student's t test. Ns, not significant. Source data are provided as a Source Data file.

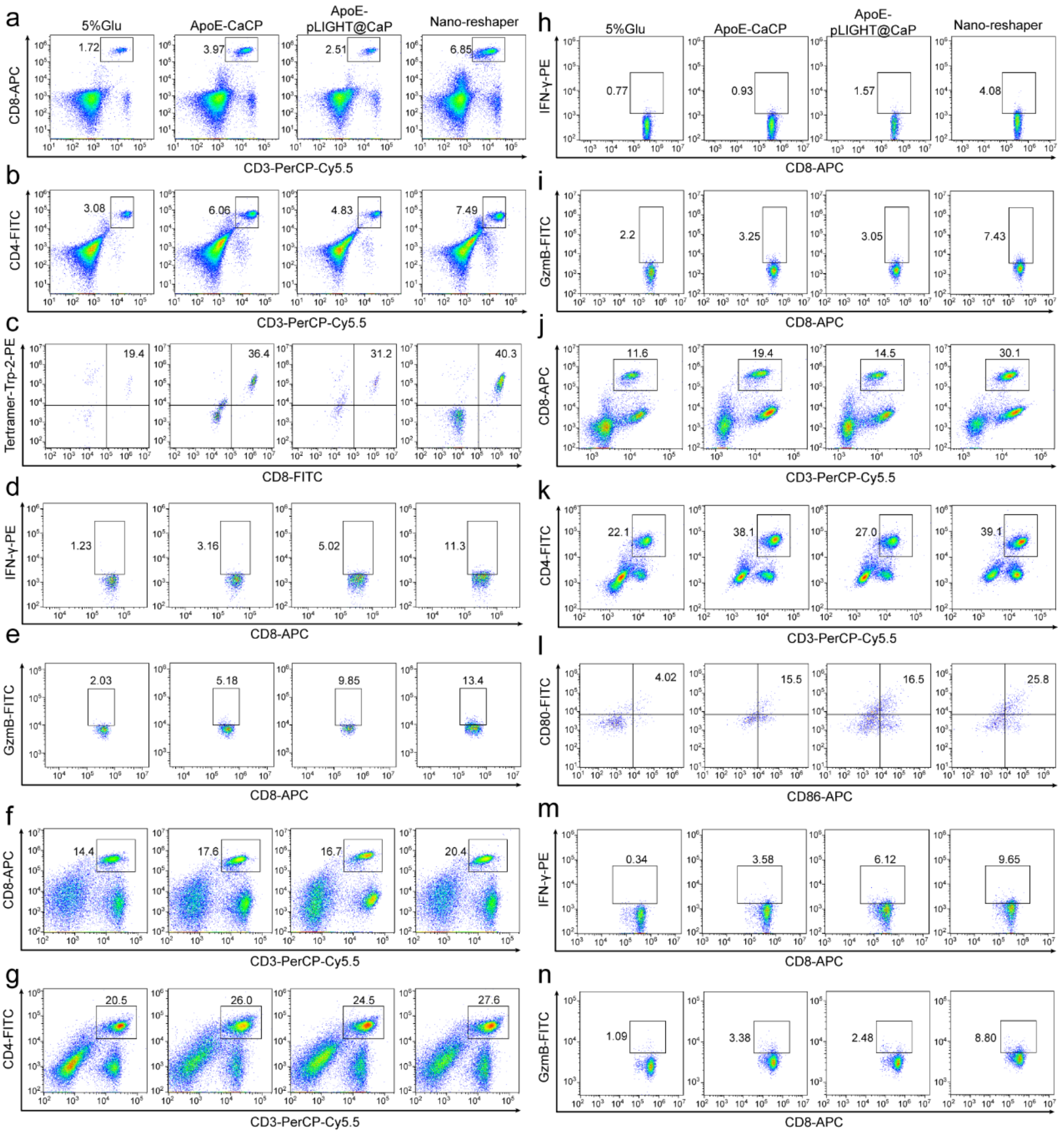

Supplementary Figure 22 Representative flow cytometry plots of peripheral blood, spleens and DLNs samples. Flow cytometry plots of $CD3^+CD8^+$ (a), $CD3^+CD4^+$ (b), $CD3^+CD8^+$Tetramer-Trp-2^+ (c), $CD3^+CD8^+$IFN-γ^+ (d) and $CD3^+CD8^+GzmB^+$ (e) cells in peripheral blood. Flow cytometry plots of $CD3^+CD8^+$ (f), $CD3^+CD4^+$ (g), $CD3^+CD8^+$IFN-γ^+ (h) and $CD3^+CD8^+GzmB^+$ (i) cells in spleens. Flow cytometry plots of $CD3^+CD8^+$ (j), $CD3^+CD4^+$ (k), $CD11c^+CD80^+CD86^+$ (l), $CD3^+CD8^+$IFN-γ^+ (m) and $CD3^+CD8^+GzmB^+$ (n) cells in DLNs.

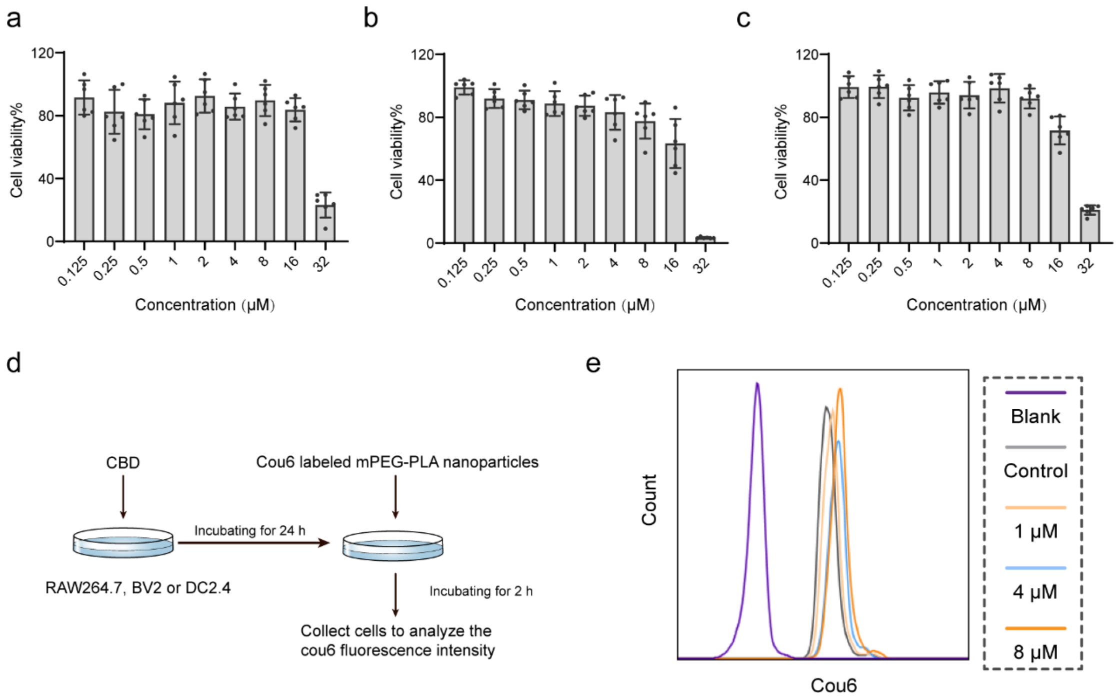

Supplementary Figure 23 The cytotoxicity and activation effects of CBD on APCs. MTT assays of (a) RAW264.7, (b) BV2 and (c) DC2.4 cells following 24 h incubation with CBD ($n = 6$ samples per group). (d) Scheme for illustration of treatment with CBD to investigate the activation effect on APCs. (e) Flow cytometry analysis of cou6 fluorescence intensity in DC2.4 cells after treatment with various concentrations of CBD. Data were shown as mean ± SD. Error bars represent SD. Source data are provided as a Source Data file.

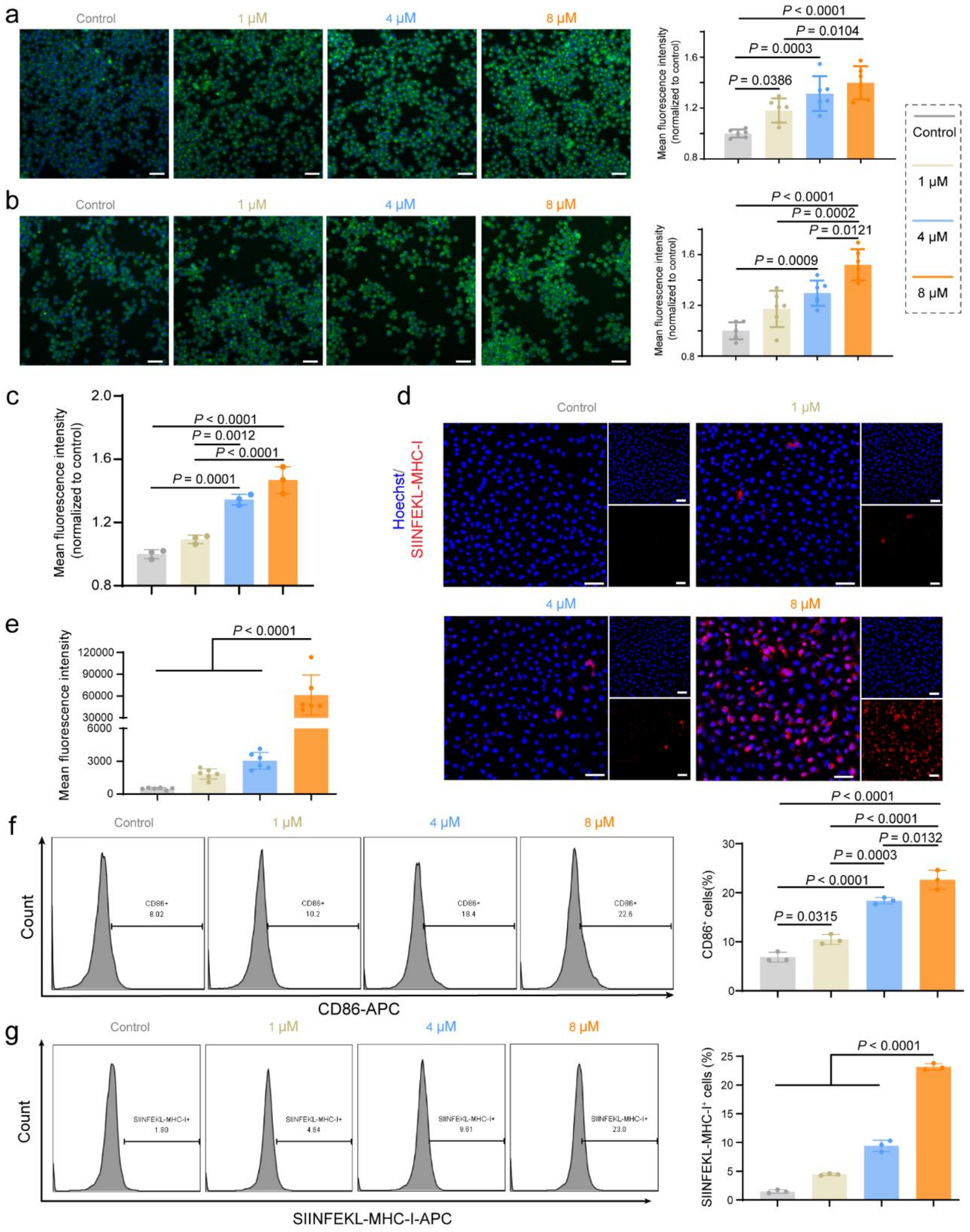

Supplementary Figure 24 CBD activated APCs and improved antigen cross-presentation. Representative images and quantitative cellular uptake of (a) BV2 and (b) RAW264.7 cells incubated with cou6-labeled mPEG-PLA nanoparticles after pre-treatment with various concentrations of CBD (*n* = 6 samples per group). Scale bar, 50 μm. The experiments were repeated three times independently. (c) Quantitative cellular uptake of cou6-labeled mPEG-PLA nanoparticles by DC2.4 cells (*n* = 3 samples per group). (d) Immunofluorescence staining of SIINFEKL-MHC-I on DC2.4 cells after pre-treatment with various concentration of CBD and OVA antigen. To do this assay, DC2.4 cells were plated at 10^5 cells/well in a 24-well plate and cultured at various

concentration of free CBD for 24 h. The medium was replaced with 100 μg/mL OVA and incubated for another 12 h. After that, the cells were washed, fixed with 4% paraformaldehyde and blocked with 5% goat serum albumin at room temperature for 1 h. Then the cells were incubated with anti-mouse SIINFEKL/H-2K^b monoclonal antibody 25-D1.16 overnight at 4°C. Finally, the cells were stained with Hoechst 33258 and observed using fluorescence microscope. Scale bar, 50 μm. The experiments were repeated three times independently. (e) Semiquantitative results of the expression of SIINFEKL-MHC-I on DC2.4 cells (n = 6 samples per group). (f) Flow cytometry analysis of CD86 expression in DC2.4 cells after treatment with various concentration of CBD (n = 3 samples per group). (g) Flow cytometry analysis of SIINFEKL-MHC-I expression in DC2.4 cells after pre-treatment with various concentration of CBD (n = 3 samples per group) for 24 h. DMEM medium containing 0.1% DMSO instead of CBD was set as the control group. Data were shown as mean ± SD. Error bars represent SD. Significant differences were evaluated in (a-c) and (e-g) using one-way ANOVA with Tukey multiple comparisons post-test. Source data are provided as a Source Data file.

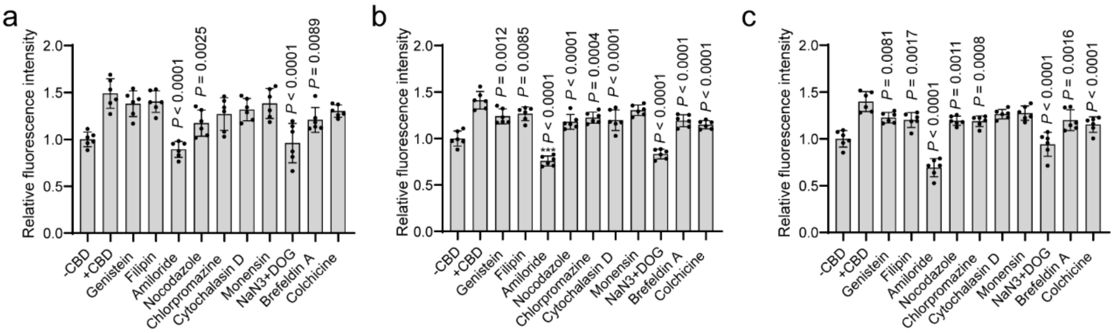

Supplementary Figure 25 Macropinocytosis pathway was mainly activated in various APCs after treatment with CBD. Cellular uptake of cou6 labeled mPEG-PLA nanoparticles by (a) RAW264.7, (b) BV2 and (c) DC2.4 cells in the presence of various endocytosis inhibitors (n = 6 samples per group). Fluorescence intensity of cou6 in cells pre-treated with DMEM medium containing 0.1% DMSO was set as "-CBD" group and cells pre-treated with 8 μM CBD without the following incubation of endocytosis inhibitors was set as "+CBD" group. Data were shown as mean ± SD. Error bars represent SD. Significant differences were evaluated in (a-c) using one-way ANOVA with Dunnett multiple comparisons post-test. Significance was compared with "+CBD" group. Source data are provided as a Source Data file.

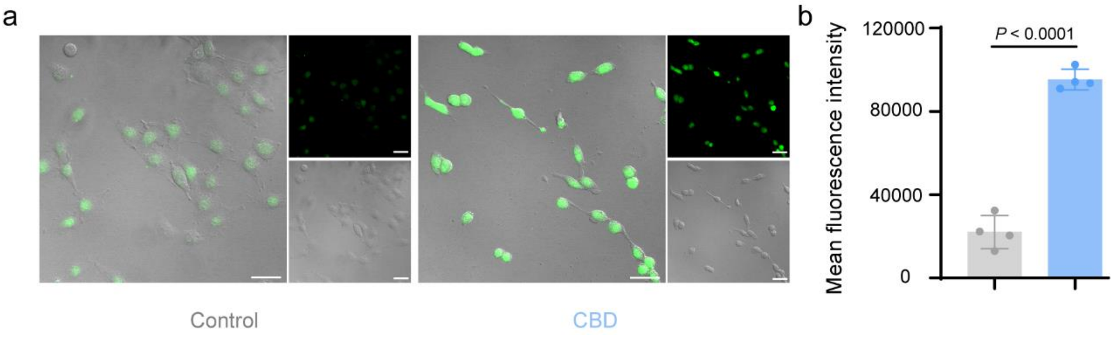

Supplementary Figure 26 CBD enhanced intracellular calcium level in DC2.4 cells.

(a) Representative images of DC2.4 cells after treatment with 8 μM CBD for 24 h and imaged by Fluo-3 AM. DMEM medium containing 0.1% DMSO was set as the control group. Scale bar, 50 μm. (b) Semi-quantitative results of calcium imaging fluorescence intensity ($n = 4$ samples per group). Data were shown as ± SD. Error bars represent SD. Significant difference was evaluated in (b) using two-tailed unpaired Student's t test. Source data are provided as a Source Data file. The experiments were repeated three times independently.

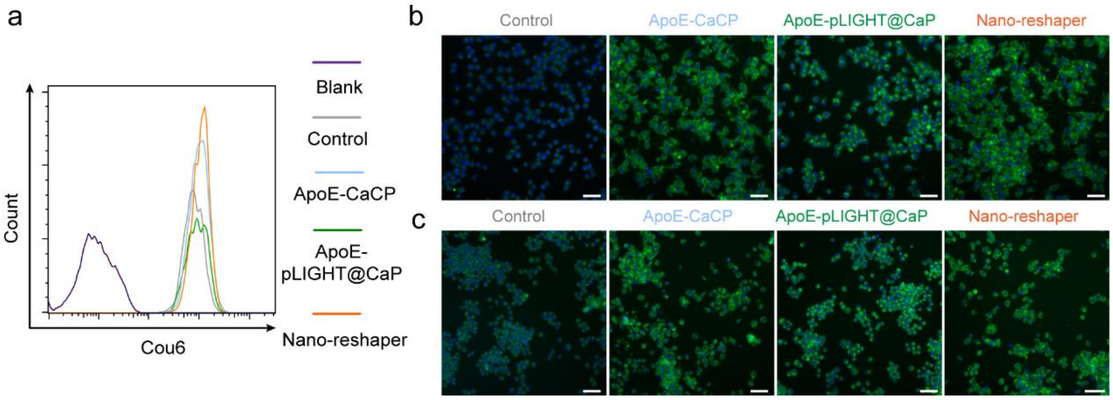

Supplementary Figure 27 The impact of various formulations on phagocytic capacity of APCs. (a) Flow cytometry analysis of cou6 fluorescence intensity in BMDCs. Representative images of (b) BV2 and (c) RAW264.7 cells incubated with cou6 labeled mPEG-PLA nanoparticles after treatment with various formulations. Scale bar, 50 μm. The experiments were repeated three times independently.

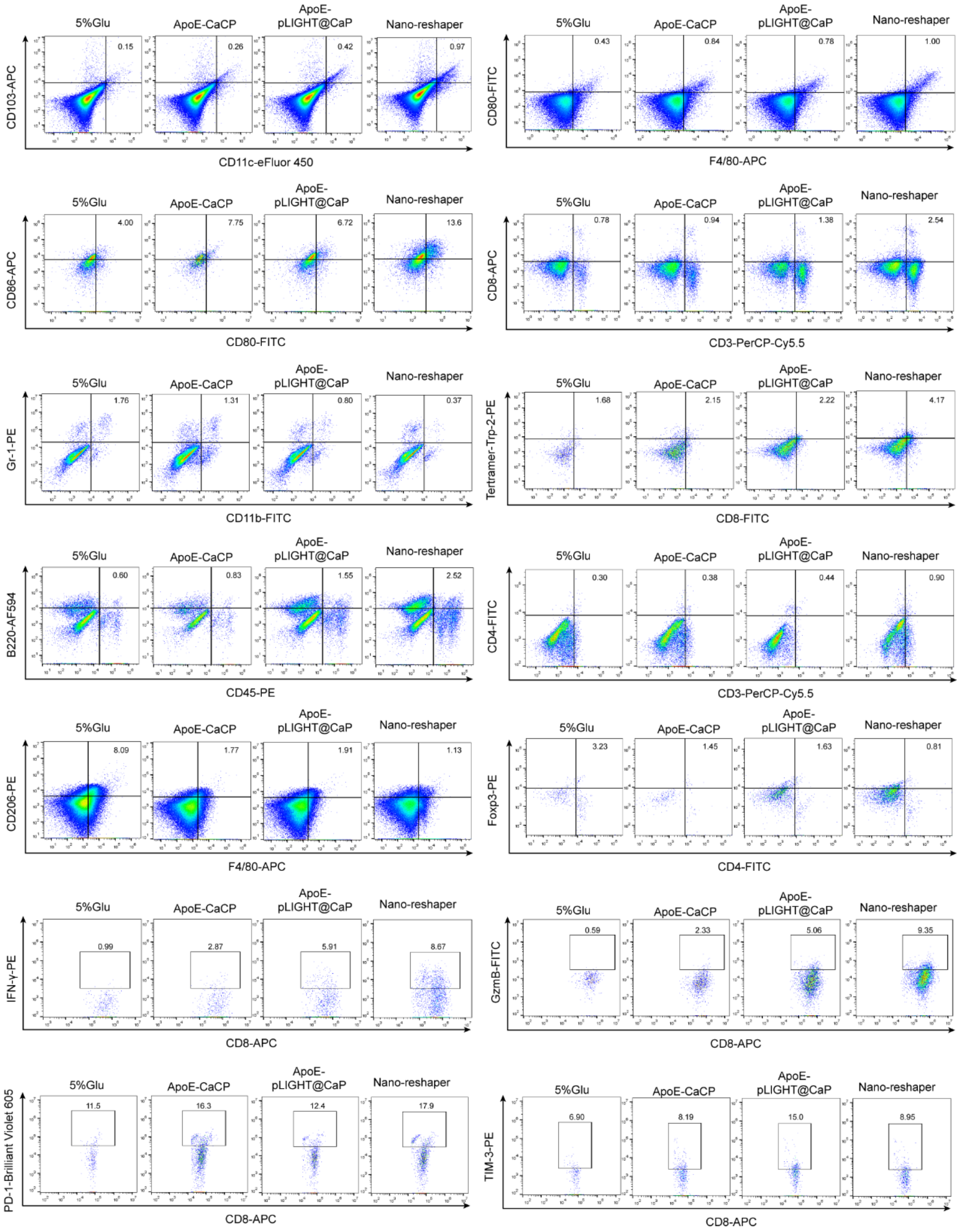

Supplementary Figure 28 Representative flow cytometry plots of brain tumor samples. Flow cytometry plots of CD11c$^+$CD103$^+$, CD11$^+$CD80$^+$CD86$^+$, CD11b$^+$Gr-1$^+$, CD45$^+$B220$^+$, F4/80$^+$CD80$^+$, F4/80$^+$CD206$^+$, CD3$^+$CD8$^+$, CD3$^+$CD8$^+$Tetramer-Trp-2$^+$, CD3$^+$CD4$^+$, CD3$^+$CD4$^+$Foxp3$^+$, CD3$^+$CD8$^+$IFN-γ$^+$, CD3$^+$CD8$^+$GzmB$^+$, CD3$^+$CD8$^+$PD-1$^+$ and CD3$^+$CD8$^+$TIM-3$^+$ cells in brain tumors.

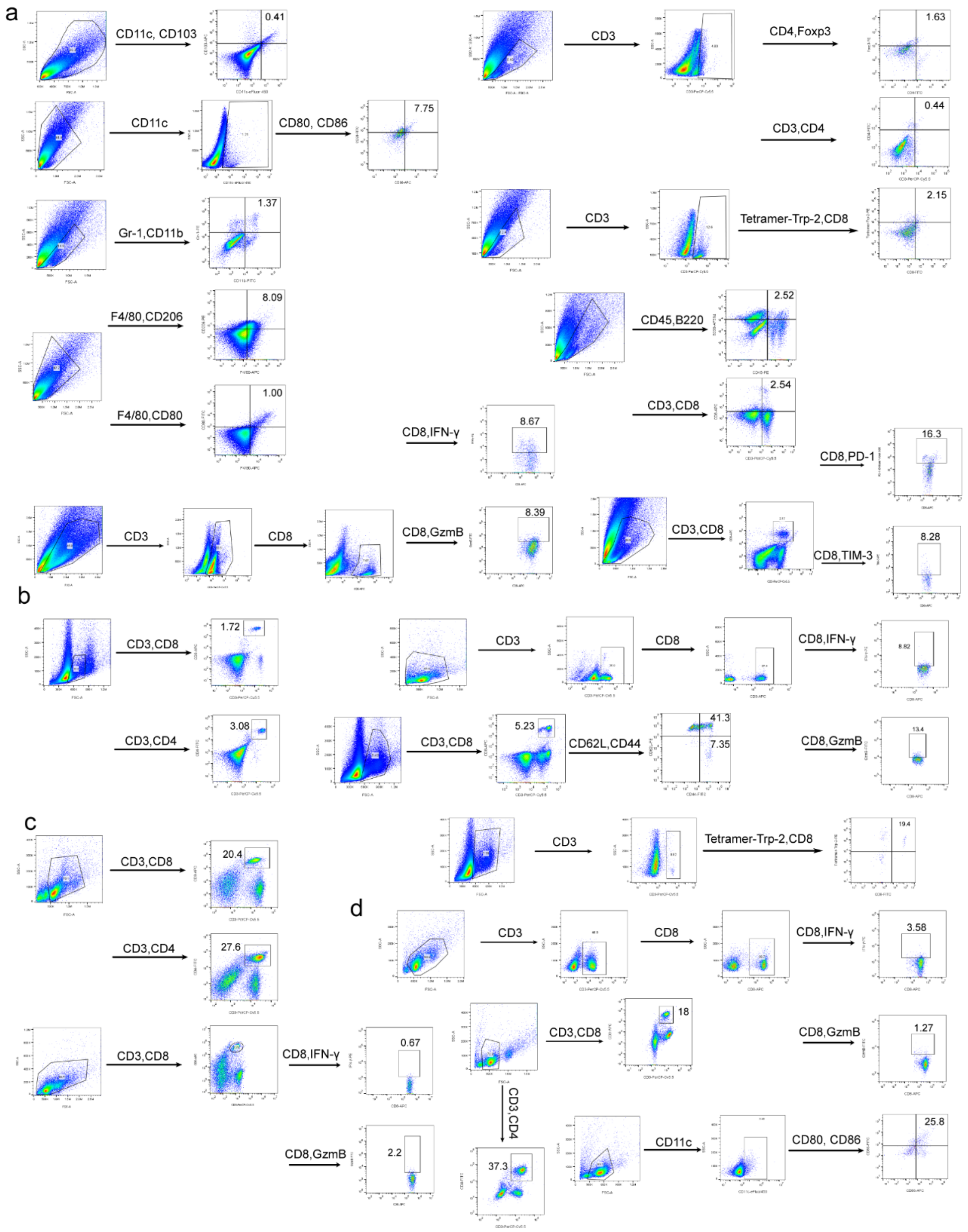

Supplementary Figure 29 Gating strategies used for flow cytometry analysis of immune cells collected from brain tumors, peripheral blood and immune organs. (a) Gating strategies to analyze CD11c$^+$CD103$^+$, CD11$^+$CD80$^+$CD86$^+$, CD11b$^+$Gr-1$^+$, CD45$^+$B220$^+$, F4/80$^+$CD80$^+$, F4/80$^+$CD206$^+$, CD3$^+$CD8$^+$, CD3$^+$CD8$^+$Tetramer-Trp-2$^+$, CD3$^+$CD4$^+$, CD3$^+$CD4$^+$Foxp3$^+$, CD3$^+$CD8$^+$IFN-γ$^+$, CD3$^+$CD8$^+$GzmB$^+$, CD3$^+$CD8$^+$PD-1$^+$ and CD3$^+$CD8$^+$TIM-3$^+$ cells in brain tumors presented in Fig. 6i. (b) Gating strategies to analyze CD3$^+$CD8$^+$, CD3$^+$CD4$^+$, CD3$^+$CD8$^+$IFN-γ$^+$, CD3$^+$CD8$^+$GzmB$^+$ and CD3$^+$CD8$^+$Tetramer-Trp-2$^+$ cells in peripheral blood presented in Fig. 4g-j. (c) Gating strategies to analyze CD3$^+$CD8$^+$, CD3$^+$CD4$^+$, CD3$^+$CD8$^+$IFN-

γ^+ and $CD3^+CD8^+GzmB^+$ cells in spleens presented in Fig. 4d-f. (d) Gating strategies to analyze $CD3^+CD8^+$, $CD3^+CD4^+$, $CD3^+CD8^+IFN\text{-}\gamma^+$, $CD3^+CD8^+GzmB^+$ and $CD11^+CD80^+CD86^+$ cell in DLNs presented in Fig. 4k-n.

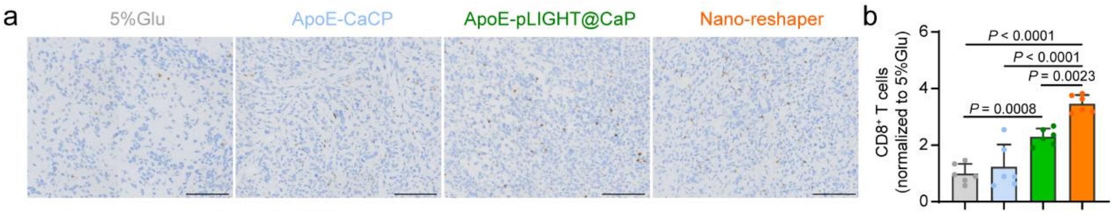

Supplementary Figure 30 $CD8^+$ T cells penetrated into GBM tumor bed. (a) Representative IHC images of $CD8^+$ T cells at the orthotopic GBM tumor sites after 5-time indicated treatment. Scale bar, 100 μm. (b) Positive cells were quantified by Image J software (n = 6 samples per group). Data were shown as mean ± SD. Error bars represent SD. Significant difference was evaluated in (b) using one-way ANOVA with Tukey multiple comparisons post-test. Source data are provided as a Source Data file. The experiments were repeated three times independently.

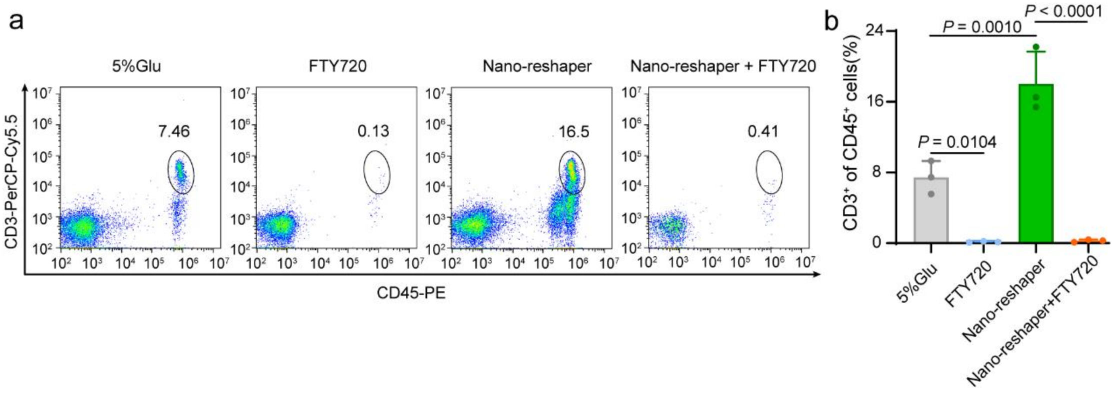

Supplementary Figure 31 FTY720 treatment downregulated T cell level in peripheral blood. (a) Representative flow cytometry plots of peripheral blood samples. (b) $CD45^+CD3^+$ cells in peripheral blood after receiving various formulations on day 12, analyzed by flow cytometry (n = 3 mice). Data were shown as mean ± SD. Error bars represent SD. Significant difference was evaluated in (b) using one-way ANOVA with Tukey multiple comparisons post-test. Source data are provided as a Source Data file.

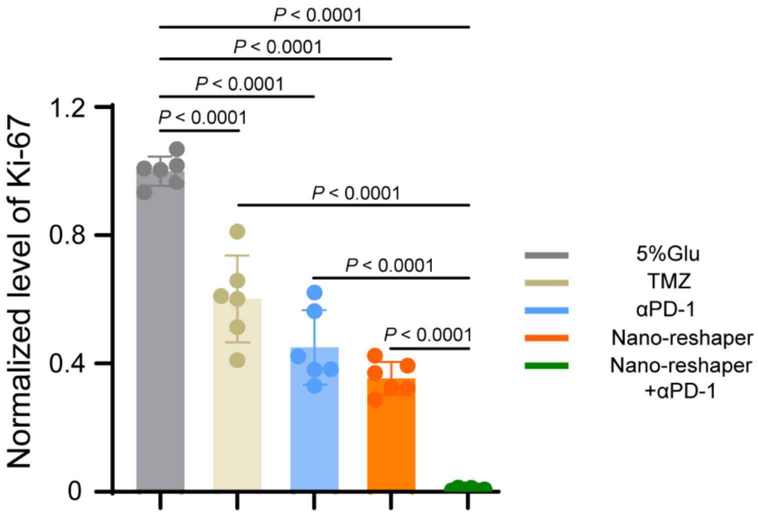

Supplementary Figure 32 Nano-reshaper synergized with αPD-1 to reduce the expression of Ki-67 in brain tumors. Semi-quantitative of Ki-67 expression in brain tumors ($n = 6$ samples per group). Data were shown as mean ± SD. Error bars represent SD. Significant differences were evaluated using one-way ANOVA with Tukey multiple comparisons post-test. Source data are provided as a Source Data file.

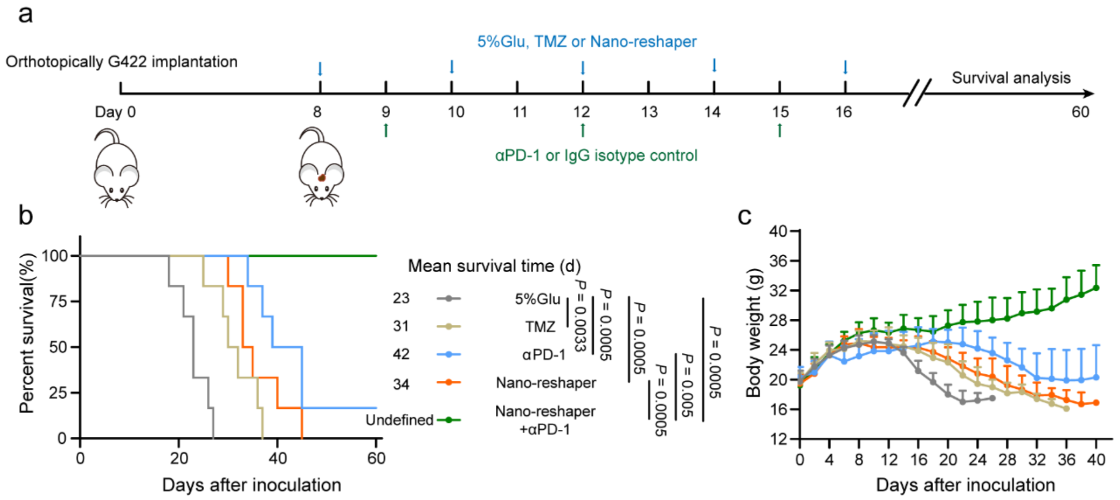

Supplementary Figure 33 Nano-reshaper synergized with αPD-1 to inhibit GBM growth on G422 orthotopic model. (a) A scheme to illustrate construction of intracranial G422 GBM and administration schedule of various formulations. (b) Kaplan-Meier survival curve of intracranial G422 GBM-bearing mice receiving various treatments ($n = 6$ mice). (c) Changes in the body weight of mice bearing intracranial G422 GBM during the therapeutic period ($n = 6$ mice). Data were shown as mean ± SD. Error bars represent SD. Significant differences were evaluated in (b) using Kaplan-Meier analysis with log-rank test. Source data are provided as a Source Data file.

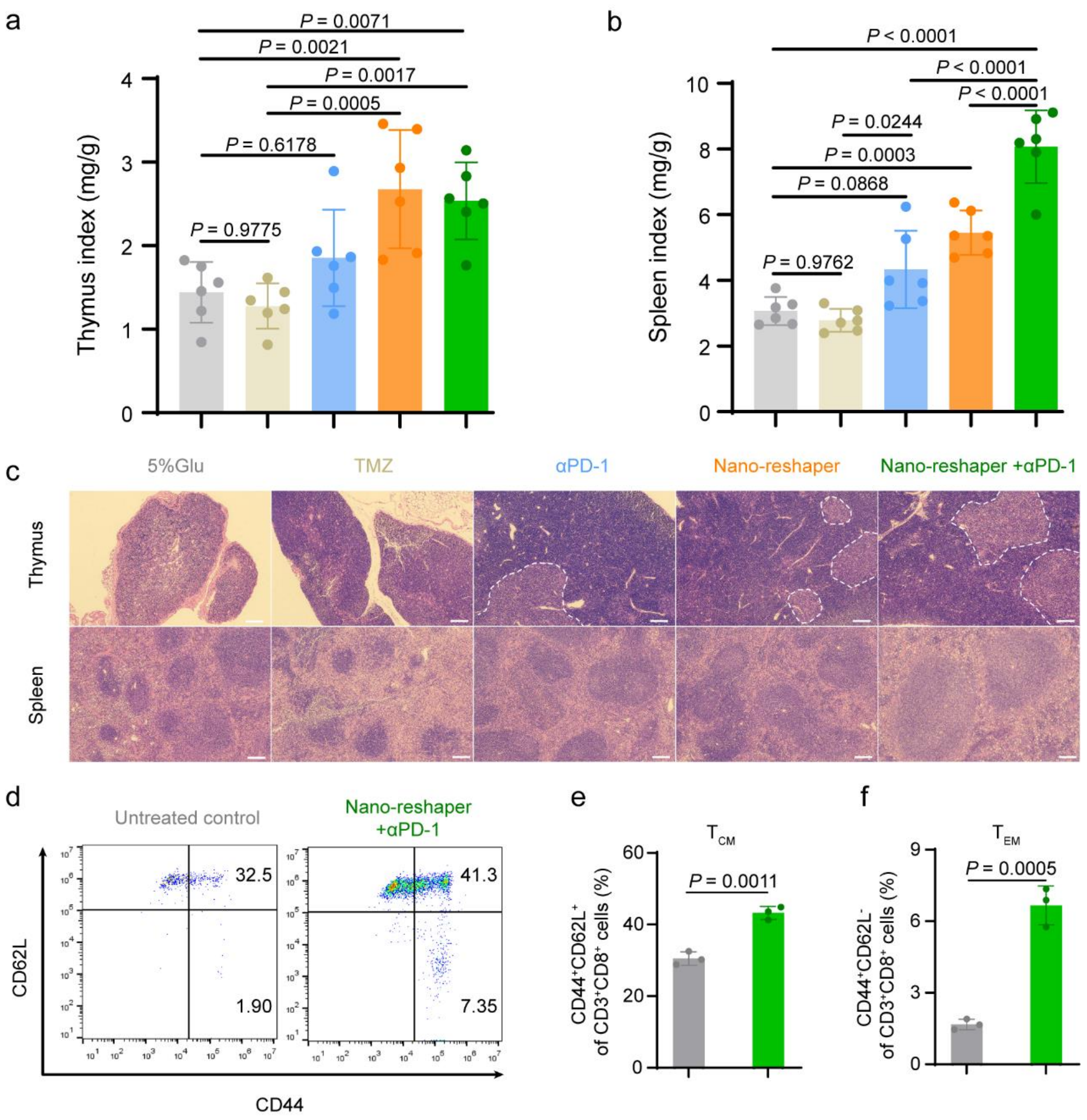

Supplementary Figure 34 Nano-reshaper synergized with αPD-1 to increase immune indexes and improve immunological memory responses. (a) Thymus index and (b) spleen index of mice treated with various formulations (*n* = 6 mice). (c) H&E staining histology to determine the impact of various formulations on thymus and spleen. Scale bar, 100 µm. The experiments were repeated three times independently. (d) Representative flow plots of T_{CM} cells ($CD3^+CD8^+CD44^+CD62L^+$) and T_{EM} cells ($CD3^+CD8^+CD44^+CD62L^-$) in the blood circulation of untreated control and Nano-reshaper + αPD-1 treated mice assessed at 16 day after GL261 glioma rechallenge. Quantitative of (e)T_{CM} and (f)T_{EM} in peripheral blood (*n* = 3 samples per group). Data were shown as mean ± SD. Error bars represent SD. Significant differences were evaluated in (a) and (b) using one-way ANOVA with Tukey multiple comparisons post-test, in (e) and (f) using two-tailed unpaired Student's *t* test. Source data are provided as a Source Data file.

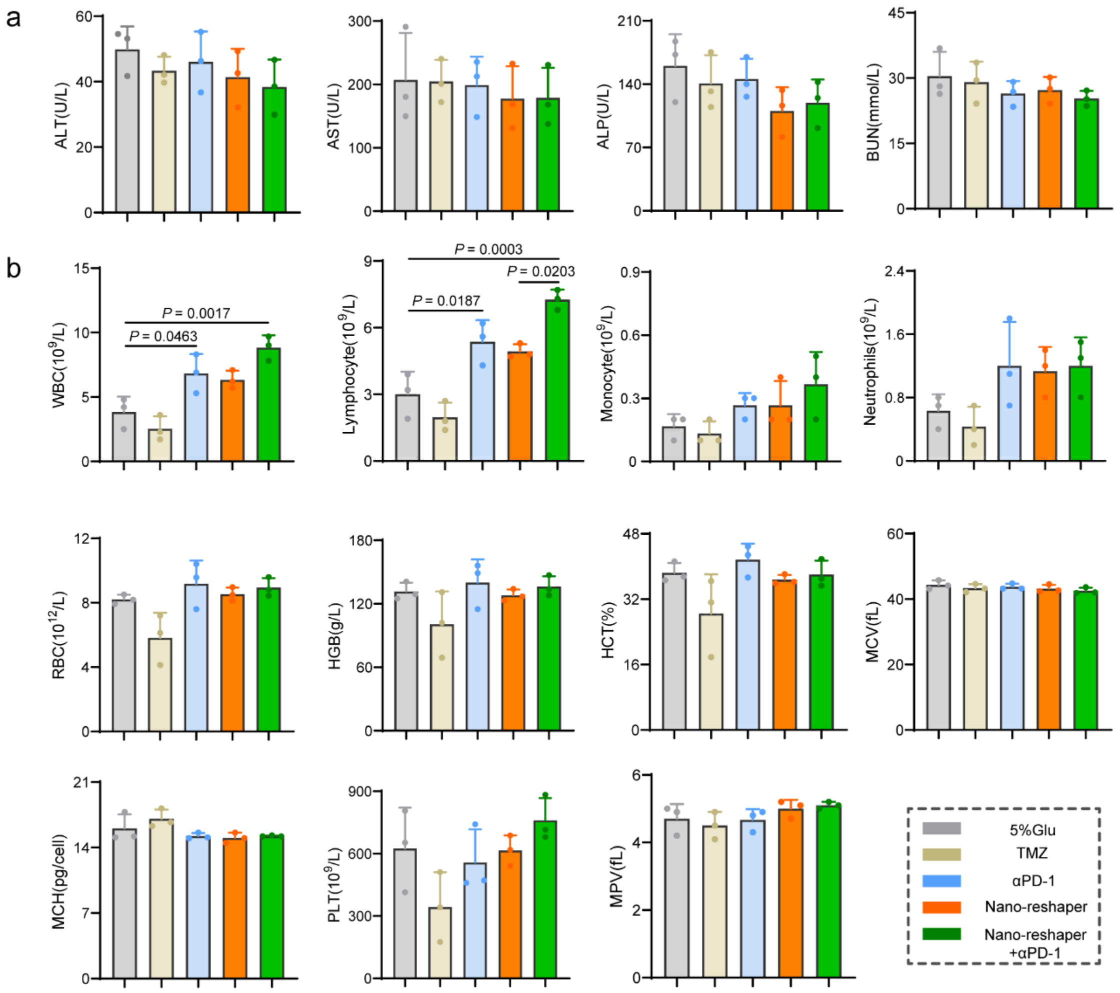

Supplementary Figure 35 Hematology analysis of healthy mice after treatment with 5%Glu, TMZ, αPD-1, Nano-reshaper and Nano-reshaper + αPD-1. (a) Blood biochemistry analysis (*n* = 3 mice). ALT: alanine aminotransferase, AST: aspartate aminotransferase, ALP: alkaline phosphatase, BUN: blood urine nitrogen. (b) Hematological indices of healthy mice at 1 d after the last treatment (*n* = 3 mice). WBC: white blood cell, RBC: red blood cell, HGB: hemoglobin, HCT: hematocrit, MCV: mean corpuscular volume, MCH: mean corpuscular hemoglobin, PLT: platelets, MPV: mean platelet volume. Data were shown as mean ± SD. Error bars represent SD. Significant differences were evaluated using one-way ANOVA with Tukey multiple comparisons post-test. Source data are provided as a Source Data file.

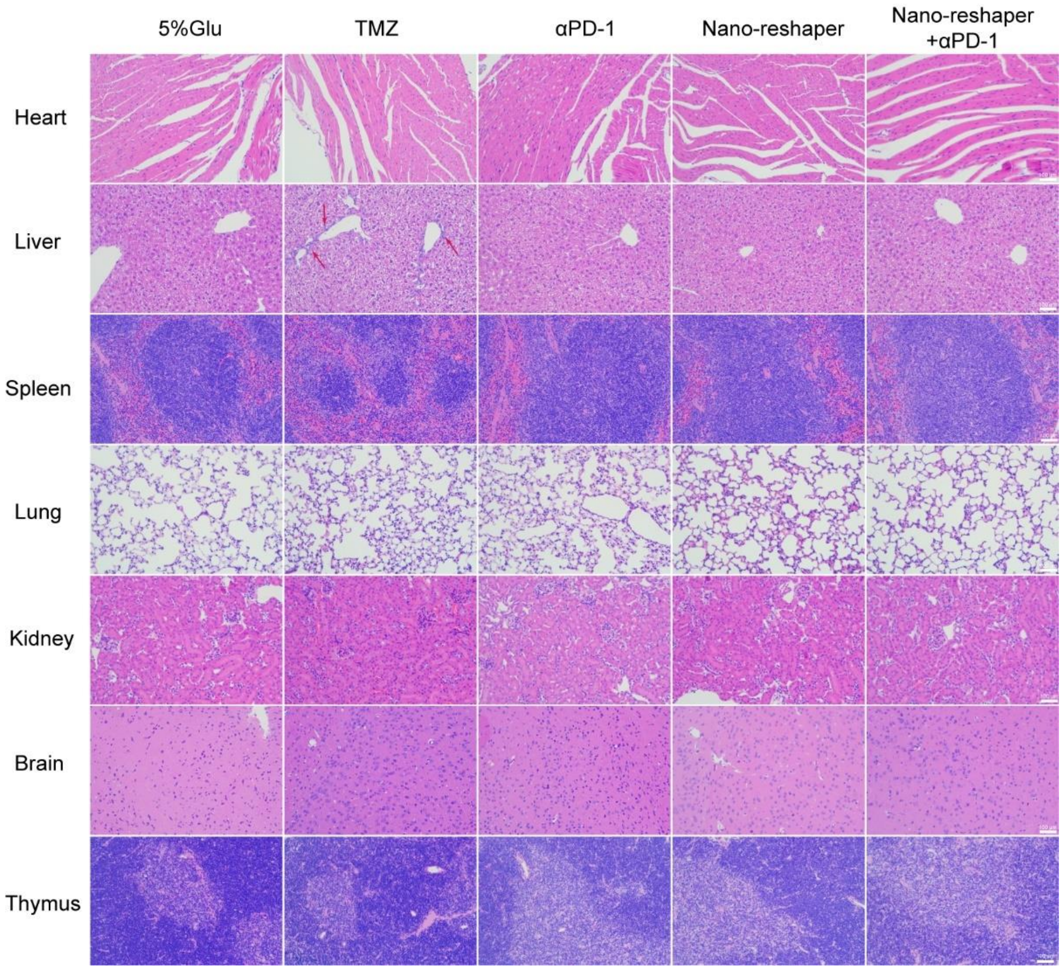

Supplementary Figure 36 H&E staining to assess the toxicity of various formulations on major organs. Infiltration of inflammation cells was observed in livers in mice receiving TMZ. Scale bar, 100 μm. The experiments were repeated three times independently.

Supplementary Table 1. Characterization of various nanoparticles.

Formulation	pLIGHT@CaCP	ApoE-CaCP	ApoE-pLIGHT@CaP	Nano-reshaper
Size (nm)	31.0 ± 4.9	34.2 ± 5.0	33.7 ± 3.1	35.9 ± 1.5
PDI	0.284 ± 0.010	0.262 ± 0.011	0.273 ± 0.018	0.250 ± 0.021
Zeta potential (mv)	-8.6 ± 1.8	15.8 ± 1.7	14.3 ± 2.0	13.1 ± 2.1
EE of pDNA (%)	49.9 ± 2.3	\	50.9 ± 4.4	51.7 ± 4.8
EE of CP (%)	52.2 ± 4.7	52.1 ± 3.6	\	53.4 ± 5.5
LE of pDNA (%)	0.21 ± 0.02	\	0.22 ± 0.02	0.21 ± 0.02
LE of CP (%)	1.49 ± 0.23	1.48 ± 0.21	\	1.45 ± 0.18

Data were shown as mean ± SD (n = 3 samples per group).

Supplementary Table 2. Antibodies used in this research.

Antibody	Company	Catalog	Application	Dilution
PE anti-mouse CD45	Biolegend	103106	FC	1:100
PerCP-Cy5.5 anti-mouse CD3ε	eBioscience	45-0031-82	FC, IHC-F	1:20
AF647 anti-mouse CD8α	Biolegend	100724	FC	1:200
AF488 anti-mouse CD4	Biolegend	100423	FC	1:500
PE/Cyanine7 anti-mouse CD4	Biolegend	100421	FC	1:100
AF594 anti-mouse B220	Biolegend	103254	FC	1:200
PE anti-mouse IFN-γ	eBioscience	12-7311-82	FC	1:100
FITC anti-mouse Granzyme B	Biolegend	515403	FC	1:40
PE anti-mouse CD366 (TIM-3)	Biolegend	134003	FC	1:100
Brilliant Violet 605™ anti-mouse CD279 (PD-1)	Biolegend	135220	FC	1:200
PE anti-mouse Foxp3	eBioscience	12-4771-82	FC	1:800
APC anti-mouse CD103	Biolegend	121413	FC	1:200
eFluor450 anti-mouse CD11c	eBioscience	48-0114-82	FC	1:200
APC anti-mouse CD11c	Biolegend	117309	IHC-F	1:100
APC anti-mouse CD86	eBioscience	17-0862-82	FC	1:400
FITC anti-mouse CD80	eBioscience	11-0801-82	FC	1:200
FITC anti-mouse CD11b	Biolegend	101206	FC	1:200
PE anti-mouse Ly-6G/Ly-6C (Gr-1)	Biolegend	108408	FC	1:100
PE anti-mouse CD206	eBioscience	12-2061-82	FC	1:200
FITC anti-mouse CD8	MBL	D271-4	FC	1:10
PE Tetramer-SVYDFFVWL (Trp-2)	MBL	TS-5004-1C	FC	1:20
FITC anti-mouse CD44	eBioscience	11-0441-82	FC	1:100
PE anti-mouse CD62L	eBioscience	12-0621-82	FC	1:200
APC anti-mouse H-2K^b bound to SIINFEKL	Biolegend	141606	FC, IHC-F	1:200
APC anti-mouse F4/80	Biolegend	123116	FC	1:100
Anti-CD31	Abcam	ab28364	IHC-F	1:50
PE Anti-mouse ICAM-1	eBioscience	12-0549-42	IHC-F	1:100
PE Anti-mouse VCAM-1	eBioscience	12-1069-42	IHC-F	1:100
Anti-MECA-79	Biolegend	120801	IHC-F	1:50
AF647 Goat Anti-Rabbit IgG	Abcam	ab150079	IHC-F	1:200
AF647 Goat Anti-Rat IgG	Abcam	ab150159	IHC-F	1:200
AF488 Goat Anti-Rabbit IgG	Abcam	ab150077	IHC-F	1:200
Anti-Trp-2	Abcam	ab74073	IHC-F	1:100
