# Supplementary material for: Reprogramming systemic and local immune function to empower immunotherapy against glioblastoma
Source: Nat Commun. 2023 Jan 26;14:435. doi: 10.1038/s41467-023-35957-8 (PMC9880004; doi:10.1038/s41467-023-35957-8)
Supplement: Supplementary file 2 — Reporting Summary [file 41467_2023_35957_MOESM2_ESM.pdf]

nature portfolio

Corresponding author(s): Jun Chen, Hongzhuan Chen, Xiaoling Gao

Last updated by author(s): Jan 10, 2023

Reporting Summary

Nature Portfolio wishes to improve the reproducibility of the work that we publish. This form provides structure for consistency and transparency in reporting. For further information on Nature Portfolio policies, see our Editorial Policies and the Editorial Policy Checklist.

Statistics

For all statistical analyses, confirm that the following items are present in the figure legend, table legend, main text, or Methods section.

n/a	Confirmed	
☐	☒	The exact sample size (n) for each experimental group/condition, given as a discrete number and unit of measurement
☐	☒	A statement on whether measurements were taken from distinct samples or whether the same sample was measured repeatedly
☐	☒	The statistical test(s) used AND whether they are one- or two-sided<br>*Only common tests should be described solely by name; describe more complex techniques in the Methods section.*
☒	☐	A description of all covariates tested
☐	☒	A description of any assumptions or corrections, such as tests of normality and adjustment for multiple comparisons
☐	☒	A full description of the statistical parameters including central tendency (e.g. means) or other basic estimates (e.g. regression coefficient) AND variation (e.g. standard deviation) or associated estimates of uncertainty (e.g. confidence intervals)
☐	☒	For null hypothesis testing, the test statistic (e.g. F, t, r) with confidence intervals, effect sizes, degrees of freedom and P value noted<br>*Give P values as exact values whenever suitable.*
☒	☐	For Bayesian analysis, information on the choice of priors and Markov chain Monte Carlo settings
☒	☐	For hierarchical and complex designs, identification of the appropriate level for tests and full reporting of outcomes
☒	☐	Estimates of effect sizes (e.g. Cohen's d, Pearson's r), indicating how they were calculated

Our web collection on statistics for biologists contains articles on many of the points above.

Software and code

Policy information about availability of computer code

Data collection	(1) IVIS small animal imaging system (Part number 124262) was used to collect and analyze fluorescence signals for nanoparticle accumulation. (2) DMI4000D Inverted fluorescence was used to collect IHC images. (3) Flow cytometry data were acquired with Beckman (CytoFlex S).
Data analysis	(1) GraphPad Prism v8.0, (2) FlowJo V10, (3) ImageJ 1.52a

For manuscripts utilizing custom algorithms or software that are central to the research but not yet described in published literature, software must be made available to editors and reviewers. We strongly encourage code deposition in a community repository (e.g. GitHub). See the Nature Portfolio guidelines for submitting code & software for further information.

Data

Policy information about availability of data

All manuscripts must include a data availability statement. This statement should provide the following information, where applicable:

- Accession codes, unique identifiers, or web links for publicly available datasets
- A description of any restrictions on data availability
- For clinical datasets or third party data, please ensure that the statement adheres to our policy

The Source Data underlying Figs. 2d, 2f-g, 3b, 3d, 3f, 3h, 4b-n, 5a-c, 5e-i, 6b, 6d, 6f, 6h-k, 7c-e, 7h, 7i, 7k, 7l, 8c-e, 8i, Supplementary Figs. 5a-b, 10a-b, 11a-c, 13, 12a-b, 15d, 16b, 17b-f, 18b-c, 19c, 19e, 19f, 19h-n, 20a-d, 21c, 21f-i, 23a-c, 24a-e, 24f-g, 25a-c, 26b, 30b, 31b, 32, 33b-c, 34a-b, 34e-f, 35a-b, Supplementary Table 1 are provided as a Source Data file. The remaining data are available within the Article, Supplementary Information or Source Data file. A reporting summary for this

article is provided as a Supplementary Information file. Source data are provided with this paper.

Human research participants

Policy information about studies involving human research participants and Sex and Gender in Research.

Reporting on sex and gender	N/A
Population characteristics	N/A
Recruitment	N/A
Ethics oversight	N/A

Note that full information on the approval of the study protocol must also be provided in the manuscript.

Field-specific reporting

Please select the one below that is the best fit for your research. If you are not sure, read the appropriate sections before making your selection.

[x] Life sciences [ ] Behavioural & social sciences [ ] Ecological, evolutionary & environmental sciences

For a reference copy of the document with all sections, see nature.com/documents/nr-reporting-summary-flat.pdf

Life sciences study design

All studies must disclose on these points even when the disclosure is negative.

Sample size	Sample size was investigated to achieve significant differences between groups based on means and standard deviations in preliminary studies. Based on the preliminary studies, reasonable sample sizes were chosen to ensure reliable results. These sample sizes also represent the standard practice for publication in this field and were described in figure legends. Each sample represents independent biological replicates. The sample sizes of animal experiments were approved by Institutional Animal Care and Use Committee (IACUC), School of Pharmacy, Fudan University (Shanghai, China).
Data exclusions	No exclusion criteria were incorporated in the design of the experiments for this study.
Replication	All experiments were repeated at least three times by the same individual operation. All the attempts at replication were successful.
Randomization	Groups were selected randomly in in vitro experiments. Mice were randomized blindly into different treatment groups.
Blinding	The investigators were blinded to the group allocation during these studies. And all analyses were performed by investigators blinded to the experimental conditions.

Reporting for specific materials, systems and methods

We require information from authors about some types of materials, experimental systems and methods used in many studies. Here, indicate whether each material, system or method listed is relevant to your study. If you are not sure if a list item applies to your research, read the appropriate section before selecting a response.

Materials & experimental systems

n/a	Involved in the study	
[ ]	[x]	Antibodies
[ ]	[x]	Eukaryotic cell lines
[x]	[ ]	Palaeontology and archaeology
[ ]	[x]	Animals and other organisms
[x]	[ ]	Clinical data
[x]	[ ]	Dual use research of concern

Methods

n/a	Involved in the study	
[x]	[ ]	ChIP-seq
[ ]	[x]	Flow cytometry
[x]	[ ]	MRI-based neuroimaging

Antibodies

Antibodies used	PE anti-mouse CD45, Biolegend, 103106, FC 1:100<br>PerCP-Cy5.5 anti-mouse CD3ε, eBioscience, 45-0031-82 FC, IHC-F 1:20

AF647 anti-mouse CD8α, Biolegend, 100724, FC, 1:200
AF488 anti-mouse CD4, Biolegend, 100423,FC,1:500
PE/Cyanine7 anti-mouse CD4, Biolegend, 100421, FC, 1:100
AF594 anti-mouse B220, Biolegend, 103254, FC, 1:200
PE anti-mouse IFN-γ, eBioscience, 12-7311-82, FC, 1:100
FITC anti-mouse Granzyme B, Biolegend, 515403, FC, 1:40
PE anti-mouse CD366 (TIM-3), Biolegend, 134003, FC, 1:100
Brilliant Violet 605TM anti-mouse CD279 (PD-1), Biolegend, 135220, FC, 1:200
PE anti-mouse Foxp3, eBioscience, 12-4771-82, FC, 1:800
APC anti-mouse CD103, Biolegend, 121413, FC, 1:200
eFluor450 anti-mouse CD11c, eBioscience, 48-0114-82, FC, 1:200
APC anti-mouse CD11c, Biolegend, 117309, IHC-F, 1:100
APC anti-mouse CD86, eBioscience, 17-0862-82, FC, 1:400
FITC anti-mouse CD80, eBioscience, 11-0801-82, FC, 1:200
FITC anti-mouse CD11b, Biolegend, 101206, FC, 1:200
PE anti-mouse Ly-6G/Ly-6C (Gr-1), Biolegend, 108408, FC, 1:100
PE anti-mouse CD206, eBioscience, 12-2061-82, FC, 1:200
FITC anti-mouse CD8, MBL, D271-4 ,FC, 1:10
PE Tetramer-SVYDFFVWL (Trp-2) ,MBL, TS-5004-1C, FC, 1:20
FITC anti-mouse CD44 ,eBioscience, 11-0441-82, FC, 1:100
PE anti-mouse CD62L ,eBioscience, 12-0621-82, FC, 1:200
APC anti-mouse H-2Kb bound to SIINFEKL ,Biolegend, 141606, FC, IHC-F, 1:200
APC anti-mouse F4/80, Biolegend, 123116, FC ,1:100
Anti-CD31, Abcam, ab28364, IHC-F, 1:50
PE Anti-mouse ICAM-1, eBioscience, 12-0549-42, IHC-F, 1:100
PE Anti-mouse VCAM-1, eBioscience, 12-1069-42, IHC-F, 1:100
Anti-MECA-79, Biolegend, 120801, IHC-F, 1:50
AF647 Goat Anti-Rabbit IgG Abcam, ab150079, IHC-F, 1:200
AF647 Goat Anti-Rat IgG Abcam, ab150159, IHC-F, 1:200
AF488 Goat Anti-Rabbit IgG Abcam, ab150077, IHC-F ,1:200
Anti-Trp-2 Abcam, ab74073, IHC-F, 1:100

Validation

All antibodies were verified by the supplier and each lot has been quality tested. All the antibodies used are from commercial sources and have been validated by the vendors. Validation data are available on the manufacturer's website.
1. PE anti-mouse CD45 has been validated to be used for flow cytometric analysis and mentioned species reactivity with mouse (https://www.biolegend.com/en-us/products/pe-anti-mouse-cd45-antibody-100).
2. PerCP-Cy5.5 anti-mouse CD3ε has been validated to be used for flow cytometric analysis and immunohistochemistry and mentioned species reactivity with mouse (https://www.thermofisher.cn/cn/zh/antibody/product/CD3e-Antibody-clone-145-2C11-Monoclonal/45-0031-82).
3. AF647 anti-mouse CD8α has been validated to be used for flow cytometric analysis and mentioned species reactivity with mouse (https://www.biolegend.com/en-us/products/alexa-fluor-647-anti-mouse-cd8a-antibody-2699).
4. AF488 anti-mouse CD4 has been validated to be used for flow cytometric analysis and mentioned species reactivity with mouse (https://www.biolegend.com/en-us/products/alexa-fluor-488-anti-mouse-cd4-antibody-2695).
5. PE/Cyanine7 anti-mouse CD4 has been validated to be used for flow cytometric analysis and mentioned species reactivity with mouse (https://www.biolegend.com/en-us/products/pe-cyanine7-anti-mouse-cd4-antibody-1919).
6. AF594 anti-mouse B220 has been validated to be used for flow cytometric analysis and mentioned species reactivity with mouse (https://www.biolegend.com/en-us/products/alexa-fluor-594-anti-mouse-human-cd45r-b220-antibody-9620).
7.PE anti-mouse IFN-γ has been validated to be used for flow cytometric analysis and mentioned species reactivity with mouse (https://www.thermofisher.cn/cn/zh/antibody/product/IFN-gamma-Antibody-clone-XMG1-2-Monoclonal/12-7311-82).
8.FITC anti-mouse Granzyme B has been validated to be used for flow cytometric analysis and mentioned species reactivity with mouse (https://www.biolegend.com/en-us/products/fitc-anti-human-mouse-granzyme-b-antibody-6066).
9.PE anti-mouse CD366 (TIM-3) has been validated to be used for flow cytometric analysis and mentioned species reactivity with mouse (https://www.biolegend.com/en-us/products/pe-anti-mouse-cd366-tim-3-antibody-5908).
10.Brilliant Violet 605TM anti-mouse CD279 (PD-1) has been validated to be used for flow cytometric analysis and mentioned species reactivity with mouse (https://www.biolegend.com/en-us/products/brilliant-violet-605-anti-mouse-cd279-pd-1-antibody-7648).
11.PE anti-mouse Foxp3 has been validated to be used for flow cytometric analysis and mentioned species reactivity with mouse (https://www.thermofisher.cn/cn/zh/antibody/product/FOXP3-Antibody-clone-NRRF-30-Monoclonal/12-4771-82).
12.APC anti-mouse CD103 has been validated to be used for flow cytometric analysis and mentioned species reactivity with mouse (https://www.biolegend.com/en-us/products/apc-anti-mouse-cd103-antibody-4914).
13.eFluor450 anti-mouse CD11c has been validated to be used for flow cytometric analysis and mentioned species reactivity with mouse (https://www.thermofisher.cn/cn/zh/antibody/product/CD11c-Antibody-clone-N418-Monoclonal/48-0114-82).
14.APC anti-mouse CD11c has been validated to be used for immunohistochemistry analysis and mentioned species reactivity with mouse (https://www.biolegend.com/en-us/products/apc-anti-mouse-cd11c-antibody-1813).
15.APC anti-mouse CD86 has been validated to be used for flow cytometric analysis and mentioned species reactivity with mouse (https://www.thermofisher.cn/cn/zh/antibody/product/CD86-B7-2-Antibody-clone-GL1-Monoclonal/17-0862-82).
16.FITC anti-mouse CD80 has been validated to be used for flow cytometric analysis and mentioned species reactivity with mouse (https://www.thermofisher.cn/cn/zh/antibody/product/CD80-B7-1-Antibody-clone-16-10A1-Monoclonal/11-0801-82).
17. FITC anti-mouse CD11b has been validated to be used for flow cytometric analysis and mentioned species reactivity with mouse (https://www.biolegend.com/en-us/products/fitc-anti-mouse-human-cd11b-antibody-347).

18.PE anti-mouse Ly-6G/Ly-6C (Gr-1) has been validated to be used for flow cytometric analysis and mentioned species reactivity with mouse (https://www.biolegend.com/en-us/products/pe-anti-mouse-ly-6g-ly-6c-gr-1-antibody-460).
19.PE anti-mouse CD206 has been validated to be used for flow cytometric analysis and mentioned species reactivity with mouse (https://www.thermofisher.cn/cn/zh/antibody/product/CD206-MMR-Antibody-clone-MR6F3-Monoclonal/12-2061-82).
20.FITC anti-mouse CD8 has been validated to be used for flow cytometric analysis and mentioned species reactivity with mouse (https://ruo.mbl.co.jp/).
21.PE Tetramer-SVYDFFVWL (Trp-2) has been validated to be used for flow cytometric analysis and mentioned species reactivity with mouse (https://ruo.mbl.co.jp/).
22.FITC anti-mouse CD44 has been validated to be used for flow cytometric analysis and mentioned species reactivity with mouse (https://www.thermofisher.cn/cn/zh/antibody/product/CD44-Antibody-clone-IM7-Monoclonal/11-0441-82).
23.PE anti-mouse CD62L has been validated to be used for flow cytometric analysis and mentioned species reactivity with mouse (https://www.thermofisher.cn/cn/zh/antibody/product/CD62L-L-Selectin-Antibody-clone-MEL-14-Monoclonal/12-0621-82).
24.APC anti-mouse H-2Kb bound to SIINFEKL has been validated to be used for flow cytometric analysis and mentioned species reactivity with mouse (https://www.biolegend.com/en-us/products/apc-anti-mouse-h-2kb-bound-to-siinfekl-antibody-7882).
25.APC anti-mouse F4/80 has been validated to be used for flow cytometric analysis and immunohistochemistry and mentioned species reactivity with mouse (https://www.biolegend.com/en-us/products/apc-anti-mouse-f4-80-antibody-4071).
26.Anti-CD31 has been validated to be used for immunohistochemistry analysis and mentioned species reactivity with mouse (https://www.abcam.cn/cd31-antibody-ab28364.html).
27.PE Anti-mouse ICAM-1 has been validated to be used for immunohistochemistry analysis and mentioned species reactivity with mouse (https://www.thermofisher.cn/cn/zh/antibody/product/CD54-ICAM-1-Antibody-clone-HA58-Monoclonal/12-0549-42).
28.PE Anti-mouse VCAM-1 has been validated to be used for immunohistochemistry analysis and mentioned species reactivity with mouse (https://www.thermofisher.cn/cn/zh/antibody/product/CD106-VCAM-1-Antibody-clone-STA-Monoclonal/12-1069-42).
29.Anti-MECA-79 has been validated to be used for immunohistochemistry analysis and mentioned species reactivity with mouse (https://www.biolegend.com/en-us/products/purified-anti-mouse-human-pnad-antibody-2975).
30.AF647 Goat Anti-Rabbit IgG has been validated to be used for immunohistochemistry analysis and mentioned species reactivity with rabbit (https://www.abcam.cn/goat-rabbit-igg-hl-alexa-fluor-647-ab150079.html).
31. AF647 Goat Anti-Rat IgG has been validated to be used for immunohistochemistry analysis and mentioned species reactivity with rat (https://www.abcam.cn/goat-rat-igg-hl-alexa-fluor-647-ab150159.html).
32.AF488 Goat Anti-rabbit IgG has been validated to be used for immunohistochemistry analysis and mentioned species reactivity with rabbit (https://www.abcam.cn/goat-rabbit-igg-hl-alexa-fluor-488-ab150077.html).
33.Anti-Trp-2 has been validated to be used for immunohistochemistry analysis and mentioned species reactivity with mouse (https://www.abcam.cn/trp2dct-antibody-ab74073.html).

Eukaryotic cell lines

Policy information about cell lines and Sex and Gender in Research

Cell line source(s)	GL261 cell line, provided by Dr. Jianhai Jiang from School of Medicine, Fudan University (Shanghai, China);<br>G422 cell line, provided by Dr. changyou Zhan from School of Medicine, Fudan University (Shanghai, China);<br>RAW264.7, BV2, DC2.4 and bEnd.3 cells were purchased from Chinese Academy of Science Cell Bank (Shanghai, China);<br>GL261 cell line stably expressing firefly luciferase (luc) or green fluorescent protein (GFP) were obtained through transducing GL261 cells with lentivirus vector (Hanyinbt, Shanghai) having luc and puromycin resistance gene.
Authentication	Stable luciferase expression GL261 cell line (GL261-luc) was authenticated by in vivo imaging system to evaluate the luminescence intensity. GL261 cell line stably expressing GFP was authenticated by fluorescence microscope. The G422, RAW264.7, BV2, DC2.4 and bEnd.3 cell lines were not validated, cell morphology and behavior were consistent with expectations.
Mycoplasma contamination	Cells were tested monthly and found to be negative for mycoplasma contamination.
Commonly misidentified lines<br>(See ICLAC register)	No cell lines used are listed in the database of commonly misidentified cell lines.

Animals and other research organisms

Policy information about studies involving animals; ARRIVE guidelines recommended for reporting animal research, and Sex and Gender in Research

Laboratory animals	Male C57BL/6 mice (6~10 weeks old, 18~22 g) or Kunming mice (3~4 weeks old, 18~22 g) were purchased from SLAC Animal Ltd. (Shanghai, China) and raised in a pathogen-free facility with a 12 h light/dark cycle at 18-23℃and 40-60% humidity and had free access to food and water.
Wild animals	This study did not involve wild animals.
Reporting on sex	Male mice are less likely to die during the establishment of orthotopic GBM models based on our historical experience. This can reduce accidental death and help to ensure the objectivity of the studies.
Field-collected samples	This study did not involve samples collected from field.

Ethics oversight	All the animal experiments were performed in accordance with the guidelines evaluated and approved by Institutional Animal Care and Use Committee (IACUC), Fudan University School of Pharmacy (Ethical approval number :2018-03-YJ-CJ-01).

Note that full information on the approval of the study protocol must also be provided in the manuscript.

Flow Cytometry

Plots

Confirm that:

- [x] The axis labels state the marker and fluorochrome used (e.g. CD4-FITC).
- [x] The axis scales are clearly visible. Include numbers along axes only for bottom left plot of group (a 'group' is an analysis of identical markers).
- [x] All plots are contour plots with outliers or pseudocolor plots.
- [x] A numerical value for number of cells or percentage (with statistics) is provided.

Methodology

Sample preparation	i: For the in vitro cellular uptake analysis, BMDCs were collected after treatment and washed with PBS, then analyze the fluorescence signal.<br>ii: For the evaluation of antigen presentation, DC2.4 cells or BMDCs were collected after treatment and washed with PBS, then stained with antibodies.<br>iii: For in vitro transfection study, were collected after treatment and washed with PBS to detect EGFP fluorescence signals.<br>iv: For detection of immune cells in blood, peripheral blood was collected and incubated with red blood cell lysis buffer to remove blood cells, then the cells were washed with PBS and stained with the addition of fluorescently labeled antibodies.<br>v: For immune cells in tissues, tissues were collected and filtrated through 70-μm single cell strainers. As for spleens, the cells were incubated with red blood cell lysis buffer to remove blood cells. Then the cells were washed with PBS and stained with the addition of fluorescently labeled antibodies for flow cytometry analysis.
Instrument	Beckman, coulter
Software	FlowJo V10
Cell population abundance	Flow cytometry was used for quantification purposes only (i.e. no postsorting fractions were collected.
Gating strategy	For all experiments FSC-A/ SSC-A gates of the starting cell population were used to discriminate between viable cells and cell debris. Isotype control stained cells were used to distinguish between background staining and specific antibody staining.

- [x] Tick this box to confirm that a figure exemplifying the gating strategy is provided in the Supplementary Information.
